# Genetic diversity analysis of French goat populations reveals selective sweeps involved in their differentiation

**DOI:** 10.1111/age.12752

**Published:** 2018-12-13

**Authors:** C. Oget, B. Servin, I. Palhière

**Affiliations:** ^1^ GenPhySE INRA Université de Toulouse INPT, ENVT 31326 Castanet Tolosan France

**Keywords:** caprine, *Capra hircus*, diversity, evolutionary history, selection signature, SNP

## Abstract

After domestication 11 000 years ago in Asia Minor, the goat followed human migration to Europe and Asia. It was then introduced in Africa and is now raised all over the world. In this study, we exploited a dataset composed of 54 000 SNPs (Illumina goat DNA chip) to analyze the genetic diversity of 223 individuals belonging to eight French breeds (Alpine, Angora, Corse, Fossés, Poitevine, Provençale, Pyrénées and Saanen). Analyses carried out included individual‐based approaches (principal component analysis and population structure) and population‐based approaches (phylogenetic tree constructions). The results of the genetic diversity analyses revealed that French breeds are clearly differentiated, in particular, the Angora breed that originates from south west Asia. The Provençale breed shows a very original genetic pattern that could be the result of ancient admixture. Then, selection signatures were detected by identifying regions of outlying genetic differentiation between populations. Five genomic regions were detected under selection on chromosomes 5, 6, 11, 13 and 20, revealing mainly soft selective sweeps and a few hard selective sweeps and highlighting candidate genes that had been selected for during the evolutionary history of these breeds. Among them, two coat coloration genes (*ADAMTS20* and *ASIP*) and one gene related to milk composition (*CSN1S1*) were involved.

## Introduction

The goat is one of the first grazing animals to be domesticated by humans in the Fertile Crescent due to its manageable size and the ability to adapt to difficult environmental conditions. According to archeological evidence (Zeder 2008) and genetic data (Zeder 2008; Colli *et al*. 2015), the goat was domesticated some 11 000 years ago in southeast Anatolia (Turkey) and in the Zagros Mountains (Iran). Since domestication, goats have been introduced in Europe, Asia and Africa and today are raised all over the world.

In metropolitan France, goats are traditionally bred for milk used for cheese production. Among dairy livestock species (with sheep and cattle), goat was the least productive one, and its livestock therefore was restricted mostly to regions with harsh environmental conditions (e.g. drylands and mountains). For this reason, historical records on French goat livestock are scarce. In particular, the establishment of goat herdbooks and official breeds started only in 1930, a century later than sheep and cattle, with the creation of the Alpine breed. Today, the advent of molecular genetic data offers the opportunity to inform this history through the signatures it left on the genetic diversity of contemporary populations.

There are traditionally three regions for goat breeding in metropolitan France: western, southeastern and central France (Spindler 1988; Le Jaouen 2002). Fourteen goat breeds are officially recognized in France today, 11 located in metropolitan France and three in French overseas regions. In metropolitan France, three types of breeds can be distinguished. Ninety percent of the French goats belong to the Alpine or the Saanen breeds. These two industrial dairy breeds are managed within efficient breeding programs (Danchin‐Burge *et al*. 2012) and originate from eastern France and Switzerland. Most of the remaining stock is composed of local dairy breeds of moderate size (about 1000 individuals): the Corse, Poitevine and Pyrénées breeds. Finally, two local breeds with few individuals (fewer than 1000) are managed within conservation programs: the Provençale and the Fossés breeds. In addition to these dairy breeds a small population of Angora individuals exists that is reared for Mohair production. This breed originates from Turkey and is managed in an organized breeding program (Danchin‐Burge *et al*. 2012).

Few studies exist on the genetic diversity of European goat populations and, in particular, French populations. A study based on pedigree records focused on the three breeds that have a breeding program: Alpine, Saanen and Angora (Danchin‐Burge *et al*. 2012). Subsequent studies have started to exploit genetic markers to better characterize the genetic diversity and migration routes of European goats (Canon *et al*. 2006; Lenstra *et al*. 2016). These two studies were based on genotyping 30 and 27 microsatellites respectively, and both included three local populations—Rove, Corse and Pyrénées—with the addition of the Alpine breed by Canon *et al*. (2006). These studies started to highlight the genetic relationships between European goat populations, but their resolution remained limited by the high mutation rate of microsatellites and the relatively small number of markers used. In particular, specific signals of adaptation could not be pinpointed.

The recent availability of a medium density single nucleotide polymorphism (SNP) array for goats (Tosser‐Klopp *et al*. 2014) offers the opportunity to improve the characterization of genetic differentiation between goat populations and scan the genome for signatures of population‐specific adaptations (http://www.goatadaptmap.org/). In our study, we used this tool to genotype eight French goat breeds (Alpine, Angora, Corse, Fossés, Poitevine, Provençale, Pyrénées and Saanen) to characterize their genetic diversity and relatedness. In addition, the marker density of this tool allows for the detection of selection signatures specific to French breeds to improve the knowledge about their evolutionary history.

## Materials and methods

### Biological samples

Based on pedigree records, 269 *a priori* minimally related individuals from eight French caprine breeds were selected and genotyped using the GoatSNP50 BeadChip (Illumina, Inc.) developed by the International Goat Consortium (Tosser‐Klopp *et al*. 2014). Original marker positions were remapped on the recent goat reference sequence ARS1 (Bickhart *et al*. 2017).

Quality control of the dataset was performed using plink v1.90 beta (Purcell *et al*. 2007; Yang *et al*. 2011; Chang *et al*. 2015). SNPs with a call rate greater than 0.95 and a minor allele frequency (MAF) greater than 0.05 were kept. Within each breed, we identified a set of unrelated individuals by pruning pairs of individuals that had a genomic kinship coefficient greater than 0.2 (Rochus *et al*. 2018). After quality control, 46 065 SNPs and 223 animals from eight French caprine breeds were kept. The name, abbreviation and sample size of each breed are given in Table 1. Additional genotypes at markers of the GoatSNP50 Bead‐Chip of two outgroup populations—Bezoar ibex (*Capra aegagrus; n *=* *7) and Iranian Goat (*n *=* *9)—were obtained from Alberto *et al*. (2018).

**Table 1:** Name of the breeds, size of the breeds, breed acronyms, sample size, mean for each breed of Wright's inbreeding coefficient (*F*_IS_) and proportion of runs of homozygosity (*F*_ROH_)

Breed name	Breed size	Breed code	Sample size	*F*_IS_	*F*_ROH_
Alpine	450 000	ALP	45	−0.011	0.053
Angora	4000	ANG	29	0.020	0.142
Corse	29 000	CRS	29	0.020	0.030
Fossés	1040	FSS	19	0.020	0.070
Poitevine	3173	PTV	27	0.014	0.111
Provençale	999	PVC	19	0.004	0.053
Pyrénées	3297	PYR	17	0.050	0.108
Saanen	350 000	SAA	38	−0.015	0.053

### Genetic diversity analysis

For each population, plink software was used to calculate two estimators of the inbreeding coefficient of each individual: *F*_IS_ using the ‐‐het small‐sample option and *F*_ROH_ using the ‐‐homozyg command. plink was also used to perform a principal component analysis (PCA) using the ‐‐pca command.

A model‐based population structure analysis was performed using admixture v1.23 (Alexander *et al*. 2009). The optimal partitioning was evaluated by cross‐validation with the number of hypothetical populations *K* ranging from 1 to 9 (Fig. S1).

Reynolds’ distances (Reynolds *et al*. 1983) were computed between all pairs of populations. The population tree was constructed by applying neighbor‐joining (Saitou & Nei 1987) on the Reynolds distances as described by Bonhomme *et al*. (2010). These analyses were performed using hapflk v.1.3.0 (Fariello *et al*. 2013). We also computed Weir and Cockerham *F*_ST_ values (Weir & Cockerham 1984) between all pairs of populations using the hierfstat R package (Goudet 2005).

To evaluate possible admixture events between populations, a maximum likelihood tree was estimated using treemix v.1.12 (Pickrell & Pritchard 2012). The optimal number of admixture events inferred by the software was evaluated by measuring model fit as explained by Pickrell & Pritchard (2012). The fraction of the variance in relatedness between populations that is accounted for by the models, *f*, was calculated for models with an added migration event number ranging from 0 to 10 (Fig. S2).

### Selection signatures analysis

Six breeds were used for the selection signatures analysis: Alpine, Corse, Fossés, Poitevine, Pyrénées and Saanen. The Provençale and Angora breeds were not included in the analysis for reasons explained in the Results section. We considered selection signatures leading to excess differentiation in allele or haplotype frequencies between populations. Specifically, the FLK (single SNP approach) and hapFLK (haplotypic approach) tests were performed using hapflk v.1.3.0. The number of haplotype clusters used for the hapFLK genome scan was set at 40, as determined by the fastphase cross‐validation procedure (Scheet & Stephens 2006). The *P*‐values and significance of each statistic at each SNP were re‐estimated following Fariello *et al*. (2014) and as indicated in the software documentation. Tests were considered significant at the 10% false discovery rate level, estimated using the R bioconductor q‐value package (Storey *et al*. 2015).

For each significant region, local trees were computed from the FLK and hapFLK statistics (Fariello *et al*. 2013) to highlight which population had most likely been selected on, using scripts provided on the hapflk web page. In parallel, allele frequencies of each population at each SNP were investigated using plink for each significant region.

Candidate genes corresponding to protein‐coding genes were extracted from the feature table of the reference caprine genome (ftp://ftp.ncbi.nlm.nih.gov/genomes/refseq/vertebrate_mammalian/Capra_hircus/latest_assembly_versions/GCF_001704415.1_ARS1) for each significant region. Some of these genes were considered selection candidates based on their physical proximity to the most significant SNP and on their referenced biological effects in the literature.

## Results

### Genetic diversity

Two kinds of analyses were performed: intra‐breed analyses, which consider each animal individually to represent genetic variability within each breed (*F*_IS_, *F*_ROH_, PCA), and structure analyses of the global sampled population, which represent the relationship between breeds (model‐based clustering, Reynolds genetic distances, *F*_ST_, phylogenetic trees).

The inbreeding coefficients ranged from –0.015 (Saanen) to 0.050 (Pyrénées) for *F*_IS_ and from 0.030 (Corse) to 0.142 (Angora) for *F*_ROH_ (Table 1). As expected, the two coefficients were not entirely in agreement, but both revealed higher inbreeding (*F*_ROH_ > 0.1) in the Angora, Poitevine and Pyrénées breeds than in the Alpine, Corse, Provençale and Saanen breeds. A runs of homozygosity (ROH) analysis (Fig. S3) revealed a few individuals exhibiting large ROH in the Pyrénées, Provençale, Fossés and Corse populations, indicating recent consanguineous matings in these small or subdivided populations.

**Figure 1:**
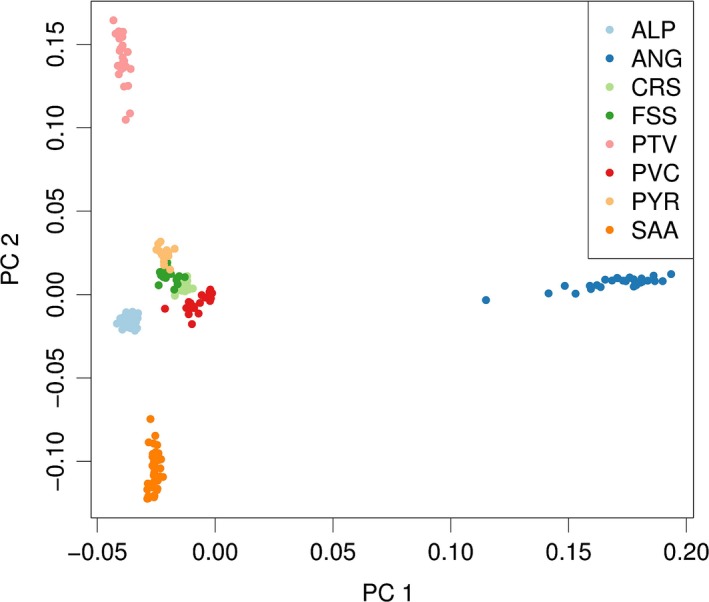
Representation of the first and second components of the principal component analysis. Breed abbreviations are given in Table 1.

A representation of the first and second principal components of the PCA is provided in Fig. 1. The first component explained 36.6% of the genetic variance in the dataset, corresponding to the divergence between Angora and the indigenous breeds (Alpine, Corse, Fossés, Poitevine, Provençale, Pyrénées and Saanen). The second component explained 13.9% of the genetic variance and separated the Poitevine and Saanen. The other components (Fig. S4) explained less than 13% of the genetic variance and corresponded to the successive separations of the other breeds, which revealed a clear genetic structure in the dataset.

**Figure 2:**
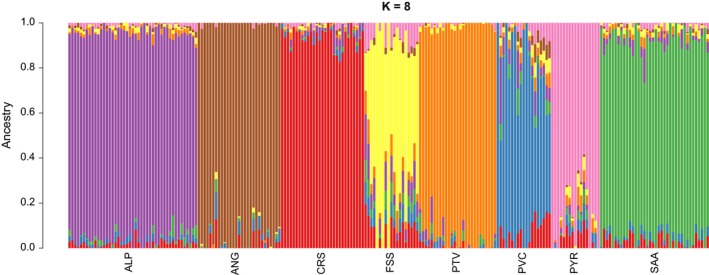
Bayesian clustering performed with admixture software on genotyping data of the selected animals. Assignment of each individual (vertical bars) to different clusters (colors) when *K *=* *8 hypothetical populations are assumed. Breed abbreviations are given in Table 1.

The cross‐validation procedure of admixture software (Fig. S1) gave an optimal number of eight hypothetical populations, *K*, corresponding to the lowest cross‐validation error (0.6418). The clustering procedure implemented in admixture software at *K *=* *8 (Fig. 2) highlights the differentiation of each breed into one clearly recognizable cluster. Among these breeds, five (Alpine, Angora, Corse, Poitevine and Saanen) formed homogeneous clusters with a very high Q score value for each individual, whereas clusters corresponding to the three other breeds (Fossés, Provençale and Pyrénées) were more heterogeneous, some individuals appearing potentially slightly admixed. This was also seen to some extent in the PCA analysis, in which individuals from these three populations exhibited larger dispersion of their loadings on PC5, PC6 and PC7 (Fig. S4).

Both computed genetic distances between each pair of populations (Fig. S5) were very well correlated (*r* ^2^ = 0.993). Reynold genetic distances and *F*_ST_ values ranged from 0.068 (the distance between Alpine and Corse) and 0.049 (the distance between Provençale and Corse) to 0.212 and 0.198 (the distance between Angora and Poitevine), with averages of 0.110 and 0.092 respectively. The Angora population was the most differentiated population with average distances of 0.185 and 0.169 for Reynolds distance and *F*_ST_ respectively, whereas the other breeds had lower average values (0.100 and 0.081 for Reynolds distance and *F*_ST_ respectively).

**Figure 3:**
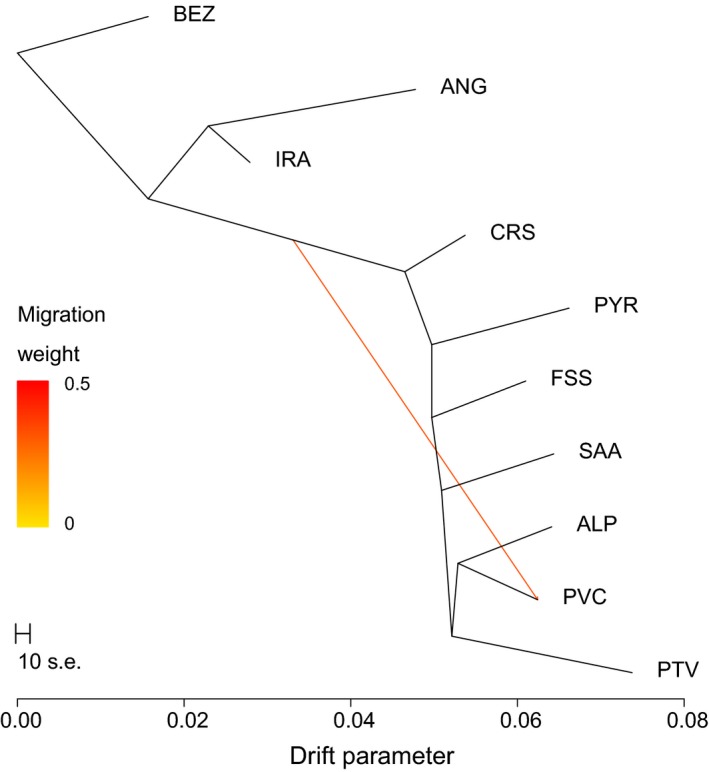
Maximum likelihood tree of the selected animals with Iranian goat (IRA) and Bezoar ibex (BEZ) populations and one unknown admixture event inferred. Breed abbreviations are given in Table 1.

To evaluate the possibility of admixture between breeds, a phylogenetic tree between populations was estimated using treemix, including Iranian goat individuals from a domestic population and from a wild population of *Capra aegagrus* (Bezoar ibex; Fig. 3). The tree showed that the wild population forms an outgroup, consistent with these individuals being descendants of the ancestral population independent of *Capra hircus* populations, similar to what was reported in sheep (Alberto *et al*. 2018; Rochus *et al*. 2018). The Angora individuals and Iranian domestics formed a sub‐clade in the tree, whereas breeds originating from France and Switzerland (hereafter called Western populations for simplicity) formed a mostly unstructured sub‐tree. Testing for possible admixture led to the inclusion of only one admixture event, after which more than 99% of the between‐population genetic variance was accounted for (Fig. S2). This admixture event linked the Provençale breed to the internal branch basal to all Western populations. Based on the shape of the curve shown in Fig. S2, we decided to add the results from treemix up to four estimated migration events (Fig. S6). These revealed links between the Saanen breed with the internal branch basal to all Western populations, the Fossés breed with the Angora branch and the Pyrénées breed with the Angora breed for the second, third and fourth migration events respectively.

### Detection of selection signatures

**Figure 4:**
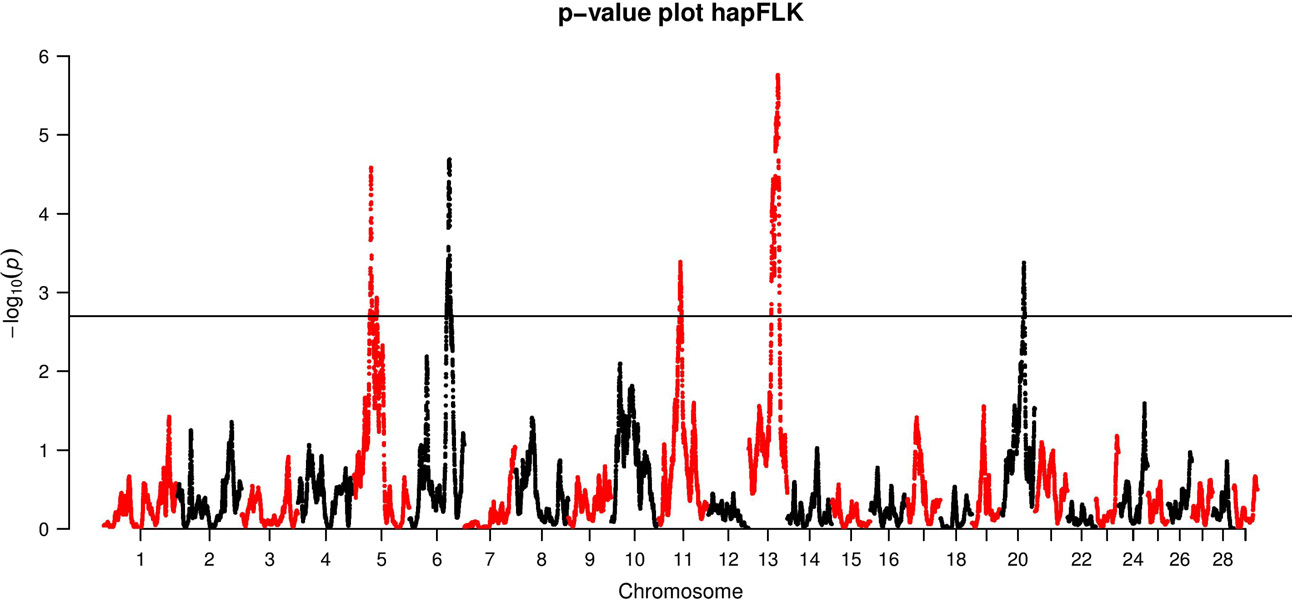
Genome scan for selection signature in six French breeds genotyped. Color change represents transition from one chromosome to the next.

Based on the genetic structure analysis, we did not consider the Angora population for the selection signature analysis, as it was too distantly related to the other populations. We also excluded the Provençale, as it was possibly admixed, a factor that is not well accounted for with the statistics used. The single‐SNP approach (FLK) revealed only two significant SNPs on chromosome 5 at positions 37.1 and 37.2 Mb, whereas the LD‐based approach (hapFLK) detected five significant regions across the six studied breeds (Fig. 4). A detailed description of these regions is provided in Table 2.

**Table 2:** Selection signatures in six French breeds (breed abbreviations are given in Table 1)

Chr	Begin (bp)	End (bp)	Diff. pop.	No of protein coding genes	Strong candidate gene	Position (bp)	Rank of candidate gene
5	34 657 160	49 621 986	FSS PYR	74	*ADAMTS20*	36 409 817–36 611 393	1
6	79 080 590	90 233 727	ALP CRS PYR	72	*CSN1S1*	85 978 463–85 995 270	7
11	44 937 429	49 314 966	FSS	59			
13	48 951 107	66 542 955	FSS PTV SAA	294	*ASIP*	63 228 709–63 249 542	17
20	46 495 170	50 063 724	PYR	2	*CDH9*	46 551 084–46 689 563	2

The first region was very large (about 15 Mb) and was located between positions 34.7 and 49.6 Mb on chromosome 5. The breeds that were the most differentiated were Pyrénées and Fossés (Fig. S7). Seventy‐four candidate genes were found, and one of them was a strong positional and functional candidate gene—*ADAMTS20*—related to coat color.

The second significant region was a bit smaller, spanning about 10 Mb and containing 72 candidate genes. It was located between positions 79.1 and 90.2 Mb on chromosome 6. Three breeds appeared to be differentiated at this location: Alpine, Corse and Pyrénées (Fig. S8). This region contained a strong candidate gene—*CSN1S1*—related to the secretion of the alpha‐s1 casein, responsible for milk coagulation.

The third significant region was less than 5 Mb long and was located between positions 44.9 and 49.3 Mb on chromosome 11. Only one breed was differentiated: Fossés (Fig. S9). The protein‐coding genes closest to the signal were *PAX8*,* PSD4* and a cluster of genes coding for interleukins (*IL1RN*,* IL1F10*,* IL36RN* and *IL36B*). However, we could not retain any particular functional candidate gene among the 59 candidate genes present in the region.

The fourth region was the largest region, spanning about 18 Mb. The signal was located between positions 49.0 and 66.5 Mb on chromosome 13. Three breeds were differentiated: Fossés, Poitevine and Saanen (Fig. S10). This large region contained 294 genes, and among them a strong functional candidate gene was present: the *ASIP* gene, related to coat color.

The last region was the smallest one (less than 4 Mb), located between positions 46.5 and 50.1 Mb on chromosome 20. Only one breed was differentiated: Pyrénées (Fig. S11). There were two genes in this region including only one gene known in the literature, the *CDH9* gene.

## Discussion

In this study, we conducted an analysis of the genetic relationship among goat populations raised in France and identified five genomic regions that appear as selection signatures. For these two objectives, our results must be interpreted with caution for two main reasons.

First, the individuals sampled were selected to ensure the representativeness of each breed, and we minimized the relationship between animals in the data by eliminating close relatives. However, the number of individuals within each breed remained rather small (about 28 individuals on average), although that is typical for such studies. In particular, due to the lack of knowledge of the pedigree for three breeds (Fossés, Provençale and Pyrénées), we eliminated several closely related individuals, leading to a reduced sample size (fewer than 20 animals). This confers to each individual a high weight in the results.

Second, the results obtained depended on the ascertainment bias of SNPs selected to be included on the caprine BeadChip (Tosser‐Klopp *et al*. 2014). These SNPs have been chosen in the context of genetic breeding to have a high MAF (mostly >0.25) across many breeds so that the SNP chip can be useful for many breeds around the world. This selection is not representative of the global caprine genome, for which a vast majority of SNPs has a very low MAF (<0.05; Benjelloun *et al*. 2015; Alberto *et al*. 2018). Thus, we might have worried about the BeadChip slightly overestimating the within‐breed genetic parameters estimated. However, most of our results were based on the genetic differentiation between populations, which is less affected by SNP ascertainment. However, this phenomenon could explain that single‐SNP FLK tests were less powerful than the hapFLK tests, which combine many SNPs to form possibly rare haplotypes (Fariello *et al*. 2013).

### Genetic diversity

The results from all genetic diversity analyses are consistent with the fact that the eight populations studied here are clearly genetically differentiated and that the Western populations are quite distantly related to the Angora population. Indeed, the genetic distances between each pair of populations are quite moderate (on average 0.110 and 0.092 for Reynolds distance and *F*_ST_ respectively), even when setting aside the Angora population (on average 0.086 and 0.066 for Reynolds distance and *F*_ST_ respectively). However, we noticed a low sample size effect in our study with the overestimation of genetic distances using the Reynolds approach in comparison with the Weir and Cockerham approach, which is not sensitive to sample size. PCA analysis confirmed the difference of the Angora population, as the first component explains 36.6% of the genetic variance and separates the Angora population from the others. Including the Iranian domestic population reveals its proximity to the Angora, consistent with its Turkish origin, and shows that these two populations separated a long time ago from the Western breeds considered here, possibly not long after domestication. Although the Angora breed in France is the result of a second wave of imports during the 1980s from several regions of the world (Canada, Texas, South Africa, Australia and New Zealand; Visser *et al*. 2016), it forms a homogeneous cluster of individuals. This is most likely due to the absence of later imports and its management in an efficient breeding program, which generally leads to homogenize within‐breed diversity.

The seven Western populations form clearly distinct genetic clusters, which can be linked to their evolution over centuries. Some breeds—Alpine, Saanen, Corse and Poitevine—appear to form a homogeneous cluster of individuals. These populations form the core of the French goat livestock for milk production and are involved in national genetic management programs with exchanges between breeders. In particular, the genetic variability of the Alpine and Saanen populations has been purposely managed in conjunction with their intensive breeding program over the last 30 years (Danchin‐Burge *et al*. 2012). The three remaining breeds, namely the Fossés, the Provençale and the Pyrénées, are in conservation programs, implemented more recently. Consequently, the genetic management and the exchange of reproducers are less intense and less organized, leading to possible between‐flock heterogeneity. For the Pyrénées breed in particular, the narrow valleys of the Pyrénées Mountains historically have limited genetic exchange between flocks. This heterogeneity comes together with higher levels of inbreeding, which would be consistent with some level of within‐breed substructure rather than actual admixture.

On the contrary, the Provençale exhibits a rather low level of inbreeding and its within‐breed heterogeneity could be better explained by a potential admixture event affecting individuals unequally. The treemix analysis indeed identified a potential source of gene flow from genetic material that appears to come from a population ancestral to Western populations. We found only one anecdotal reference dating from 1947 in which a French shepherdess who owned some Provençale goats refers to her goats as ‘Syriennes’, which means goats originating from Syria (Mauron 1947). This partially oriental origin of the breed is also mentioned by Babo (2000) but possibly from the same source. Therefore, we can make the hypothesis that the Provençale breed is a cross of some individuals already present in France with some other individuals subsequently imported from a country close to the location of original goat domestication. In the larger study by Colli *et al*. (2018), including the global Adaptmap panel (144 goat populations), the Provençale breed was always clustered with the European populations, evidence that they share a common genetic heritage. Nevertheless, the Provençale breed appeared (i) closer to the populations of central or southern Italy than to the French ones and (ii) closer to the Pakistan and Iranian breeds than to the French ones, which is consistent with our results.

Interestingly, a similar flow of ancestral genes for the Saanen and Fossés breeds was found when adding further migration edges in the treemix analysis (Fig. S6). This could indicate a common event affecting multiple breeds, but further elucidation of this was not feasible in the context of our study for two main reasons. First, identifying the potential source of admixture would require genotyping more populations. This is being carried out within the ADAPTmap initiative (Colli *et al*. 2018). The second limitation comes from the genotyping tool used. Although the SNP array used for this study is a great improvement over previous tools, it has been designed to include SNPs with high MAF in many different breeds (Tosser‐Klopp *et al*. 2014), which can introduce a bias in the population statistics calculated (*F*_IS_ and *F*_ST_). It also limits the identification of the origins of admixture, information for which could come from SNPs that would be specific to a few numbers of populations but that are not on the chip by design. Further work based on sequencing data will most likely be needed to investigate this further.

### Detection of selection signatures

We detected five significant regions along the caprine genome in our dataset. The selection signatures detected in this study thus could have resulted from natural selection or from an individual selection of breeders over the centuries. The latter concerns mainly production traits for economic reasons and morphologic traits for other reasons such as coat color, morphology, hardiness, etc. In each significant region, therefore, we looked at genes that could be related to one of these reasons more specifically.

In our detection of selection signatures, we were looking for either ‘hard sweeps’, which means a rapid fixation of an initially rare variant in a population, or ‘soft sweeps’, which means a selection on standing variation. Indeed, the hapFLK differentiation‐based approach can detect these two selective signals.

Among the 74 protein‐coding genes in the significant region on chromosome 5, the *ADAMTS20* gene (coding for a disintegrin‐like and metalloprotease with thrombospondin type‐1 motifs) was considered a good functional candidate, as it has been shown to modify coat color patterns in mice (Rao *et al*. 2003; Baxter *et al*. 2004; Silver *et al*. 2008), which match some breeds in our dataset. In addition, this gene was closest to the most significant SNP in the haplotype analysis and quite close to the two significant SNPs identified with the SNP approach, making it also a good positional candidate. In mice, mutations in the *ADAMTS20* gene cause the emergence of a belted phenotype (white spotting of the dorsal and ventral torso), which results from a defect in melanocyte development. Local examination of the allele and haplotype differentiation patterns in the signature (Fig. S7) reveals that the Pyrénées population has most likely been selected for a mutation in the region. The allele frequencies in each population at each SNP in the significant regions are provided in Table S1. One of the traditional breed standards is a belted phenotype closely matching the mice mutant. We suspect that the Pyrénées population harbors a high‐frequency variant (the phenotype is not completely homogeneous in the breed) at the *ADAMTS20* locus that created a hard selective sweep in the neighboring region. In addition to the Pyrénées, we found a possible signature of selection at the same locus in the Fossés breed, but not associated with extreme loss of diversity in the region (a soft selective sweep). This would be consistent with the phenotypic diversity of the Fossés population, within which only some animals exhibit a belted phenotype.

On chromosome 6, the *CSN1S1* gene (rank 7/72 in terms of position) was considered the best functional candidate for the selection signature. This gene codes for casein alpha s1, a protein largely responsible for milk coagulation, a fundamental step in the process of deriving cheese from raw milk. Indeed, many studies have focused on this very polymorphic locus because it is of great interest for the French dairy goat industry (Grosclaude *et al*. 1987; Barbieri *et al*. 1995; Selvaggi *et al*. 2014). The *CSN1S1* gene was characterized for the caprine species at the end of the 1980s (Brignon *et al*. 1989, 1990), which resulted in the discovery of eight alleles associated with four levels of protein synthesis. This locus might have been indirectly selected by breeders over time through the conservation of individuals with great milk coagulation potential. More recently, the *CSN1S1* genotypes have been used for selection purposes in the breeding programs of the Alpine and Saanen breeds (Manfredi & Adnøy 2012). In our study, the two populations that appear to have been selected on this gene are the Alpine and Pyrénées breeds (Fig. S8). However, they are not fixed for a single haplotype in the region, which would be consistent with multiple alleles being selected on at this locus, driving a soft selective sweep.

In the significant region on chromosome 13, the *ASIP* gene (*agouti signaling protein*), a copy number variation gene related to coat coloration, was found quite close to the most significant SNP (rank 17/294). First characterized in mice in the early 1990s (Bultman *et al*. 1992), this gene has been very well studied in the caprine species because it is responsible for the emergence of white color, to a greater or lesser degree according to its copy number in the genome (Dong *et al*. 2015). This signature of selection was recently detected in the Appenzell goat and Chamois‐colored breeds from Switzerland (Burren *et al*. 2016) as well as in the Saanen breed from Canada (Brito *et al*. 2017). Additionally, in another recent study, Martin *et al*. (2016) revealed its association with undesired coat color phenotypes in French Saanen goats. All three breeds involved in the detected signal in our study—Fossés, Poitevine and Saanen—have a white coat coloration but with different patterns: Saanen is entirely white; Poitevine has white color on the belly, legs and head; and Fossés does not have a fixed color phenotype, but there is frequently a white spot on the coat. We note however that the selection signature is quite large, and it is possible that multiple selection events, possibly affecting different genes, are responsible for the observed signal.

The selection signature on chromosome 20 lies in a gene‐poor region, not only in goat but also in cattle and human. Hence, despite this region's relatively large size, only a single gene is annotated: the *CDH9* gene coding for the cadherin 9 protein. This gene has been associated with autism in humans, consistent with an almost exclusive brain transcription. Cadherins mediate cell–cell adhesion but also are involved in intracellular signaling pathways associated with neuropsychiatric disease (Wang *et al*. 2009; Redies *et al*. 2012). In other species, this locus was found to be a selection signature in dogs (Akey *et al*. 2010) and was interpreted as being related to behavior but with no functional support. It is also possible that other genes, possibly non‐protein coding ones, are present in the region and affect some other trait.

In the current study, we confirmed three selection signatures detected by Bertolini *et al*. (2018) and found two new selection signatures never detected before. The very well‐known genes involved in coat color determination, *ADAMTS20* on chromosome 5 and *ASIP* on chromosome 13, had already been found in several groups of breeds: for the *ADAMTS20* gene, the southwestern European group (which includes the Pyrénées population; the Argentata dell'Etna breed from the southeastern European group; and the Sahel, Peuhl and Targi breeds from northwestern Africa) and for the *ASIP* gene, the Kacchan population from the Pakistani breeds and the Alpine, Poitevine and Valdostana breeds from the Alpine group. The cluster of casein genes on chromosome 6 was also detected in the group of Alpine populations and in the eastern African populations. In contrast, regions on chromosomes 13 and 20 are specific to French breeds.

## Conclusion

In our study, we characterized within‐ and between‐breed genetic diversity of eight French goat breeds with a medium density SNP chip. The analyses showed that the Angora breed is strongly different from the other French breeds due to its southwestern Asian origins. The other seven French goat breeds are genetically more closely related but still clearly differentiated. We also discovered that the Provençale breed shows a particular pattern of admixture, which will likely require sequencing data for interpretation. We detected five significant genomic regions differentially selected between breeds and three candidate genes potentially involved (*ADAMTS20*,* CSN1S1* and *ASIP*). Two of these selection signatures were reported for the first time. All the results obtained in this study can be useful for future breed management and pave the way to further genetic studies on the evolutionary history of goat populations.

## Supporting information


**Figure S1** Admixture cross‐validation procedure.

**Figure S2** Measuring models fit in treemix.

**Figure S3** Boxplots of average runs of homozygosity (ROH) lengths and number of ROH for each population.

**Figure S4** Representation of the first seven principal components of the principal component analysis.

**Figure S5** Weir & Cockerham *F*_ST_ values and Reynold's genetic distances between each pair of populations.

**Figure S6** Maximum likelihood trees of the selected animals with Iranian goat and Bezoar ibex populations and (a) two, (b) three and (c) four unknown admixture events inferred.

**Figure S7** Local population trees computed using the FLK statistic and the hapFLK statistic on the significant region of chromosome 5.

**Figure S8** Local population trees computed using the FLK statistic and the hapFLK statistic on the significant region of chromosome 6.

**Figure S9** Local population trees computed using the FLK statistic and the hapFLK statistic on the significant region of chromosome 11.

**Figure S10** Local population trees computed using the FLK statistic and the hapFLK statistic on the significant region of chromosome 13.

**Figure S11** Local population trees computed using the FLK statistic and the hapFLK statistic on the significant region of chromosome 20.

**Table S1** Allele frequencies in each population at each SNP in the significant regions.

## References

[age12752-bib-0001] Akey J.M. , Ruhe A.L. , Akey D.T. , Wong A.K. , Connelly C.F. , Madeoy J. , Nicholas T.J. & Neff M.W. (2010) Tracking footprints of artificial selection in the dog genome. Proceedings of the National Academy of Sciences of the United States of America 107, 1160–5. 20080661 10.1073/pnas.0909918107 PMC2824266

[age12752-bib-0002] Alberto F.J. , Boyer F. , Orozco‐terWengel P. *et al* (2018) Convergent genomic signatures of domestication in sheep and goats. Nature Communications 9, 813. 10.1038/s41467-018-03206-y PMC5840369 29511174

[age12752-bib-0003] Alexander D.H. , Novembre J. & Lange K. (2009) Fast model‐based estimation of ancestry in unrelated individuals. Genome Research 19, 1655–64. 19648217 10.1101/gr.094052.109 PMC2752134

[age12752-bib-0004] Babo D. (2000) Races Ovines et Caprines Françaises. France Agricole, Paris.

[age12752-bib-0005] Barbieri M.E. , Manfredi E. , Elsen J.M. , Ricordeau G. , Bouillon J. , Grosclaude F. , Mahé M.F. & Bibé B. (1995) Effects of the *α*s1‐casein locus on dairy performances and genetic parameters of Alpine goats. Genetics Selection Evolution 27, 437–50.

[age12752-bib-0006] Baxter L.L. , Hou L. , Loftus S.K. & Pavan W.J. (2004) Spotlight on spotted mice: a review of white spotting mouse mutants and associated human pigmentation disorders. Pigment Cell Research 17, 215–24. 15140066 10.1111/j.1600-0749.2004.00147.x

[age12752-bib-0007] Benjelloun B. , Alberto F.J. , Streeter I. *et al* (2015) Characterizing neutral genomic diversity and selection signatures in indigenous populations of Moroccan goats (*Capra hircus*) using WGS data. Frontiers in Genetics 6, 107. 25904931 10.3389/fgene.2015.00107 PMC4387958

[age12752-bib-0008] Bertolini F. , Servin B. , Talenti A. *et al* (2018) Signatures of selection and environmental adaptation across the goat genome post‐domestication. Genetics Selection Evolution. 10.1186/s12711-018-0421-y PMC6240954 30449276

[age12752-bib-0009] Bickhart D.M. , Rosen B.D. , Koren S. *et al* (2017) Single‐molecule sequencing and chromatin conformation capture enable *de novo* reference assembly of the domestic goat genome. Nature Genetics 49, 643–50. 28263316 10.1038/ng.3802 PMC5909822

[age12752-bib-0010] Bonhomme M. , Chevalet C. , Servin B. , Boitard S. , Abdallah J. , Blott S. & SanCristobal M. (2010) Detecting selection in population trees: the Lewontin and Krakauer test extended. Genetics 186, 241–62. 20855576 10.1534/genetics.110.117275 PMC2940290

[age12752-bib-0011] Brignon G. , Mahé M. , Grosclaude F. & Ribadeau‐Dumas B. (1989) Sequence of caprine alpha s1‐casein and characterization of those of its genetic variants which are synthesized at a high level, alpha s1‐CnA, B and C. Protein Sequences and Data Analysis 2, 181–8. 2755948

[age12752-bib-0012] Brignon G. , Mahé M.‐F. , Ribadeau‐Dumas B. , Mercier J.‐C. & Grosclaude F. (1990) Two of the three genetic variants of goat *α*s1‐casein which are synthesized at a reduced level have an internal deletion possibly due to altered RNA splicing. European Journal of Biochemistry 193, 237–41. 2226443 10.1111/j.1432-1033.1990.tb19328.x

[age12752-bib-0013] Brito L.F. , Kijas J.W. , Ventura R.V. , Sargolzaei M. , Porto‐Neto L.R. , Cánovas A. , Feng Z. , Jafarikia M. & Schenkel F.S. (2017) Genetic diversity and signatures of selection in various goat breeds revealed by genome‐wide SNP markers. BMC Genomics 18, 229. 28288562 10.1186/s12864-017-3610-0 PMC5348779

[age12752-bib-0014] Bultman S.J. , Michaud E.J. & Woychik R.P. (1992) Molecular characterization of the mouse agouti locus. Cell 71, 1195– 204. 1473152 10.1016/s0092-8674(05)80067-4

[age12752-bib-0015] Burren A. , Neuditschko M. , Signer‐Hasler H. , Frischknecht M. , Reber I. , Menzi F. , Drögemüller C. & Flury C. (2016) Genetic diversity analyses reveal first insights into breed‐specific selection signatures within Swiss goat breeds. Animal Genetics 47, 727–39. 27436146 10.1111/age.12476

[age12752-bib-0016] Canon J. , Garcia D. , Garcia‐Atance M.A. , Obexer‐Ruff G. , Lenstra J.A. , Ajmone‐Marsan P. , Dunner S. & The Econogene Consortium (2006) Geographical partitioning of goat diversity in Europe and the Middle East. Animal Genetics 37, 327–34. 16879341 10.1111/j.1365-2052.2006.01461.x

[age12752-bib-0017] Chang C.C. , Chow C.C. , Tellier L.C. , Vattikuti S. , Purcell S.M. & Lee J.J. (2015) Second‐generation plink: rising to the challenge of larger and richer datasets. GigaScience 4, 7. 25722852 10.1186/s13742-015-0047-8 PMC4342193

[age12752-bib-0019] Colli L. , Lancioni H. , Cardinali I. *et al* (2015) Whole mitochondrial genomes unveil the impact of domestication on goat matrilineal variability. BMC Genomics 16, 1115. 26714643 10.1186/s12864-015-2342-2 PMC4696231

[age12752-bib-0020] Colli L. , Milanesi M. , Talenti A. *et al* (2018) Drawing up worldwide goat diversity and post‐domestication history. Genetics Selection Evolution. 10.1186/s12711-018-0422-x PMC6240949 30449284

[age12752-bib-0021] Danchin‐Burge C. , Allain D. , Clément V. , Piacère A. , Martin P. & Palhière I. (2012) Genetic variability and French breeding programs of three goat breeds under selection. Small Ruminant Research 108, 36–44.

[age12752-bib-0022] Dong Y. , Zhang X. , Xie M. *et al* (2015) Reference genome of wild goat (*Capra aegagrus*) and sequencing of goat breeds provide insight into genic basis of goat domestication. BMC Genomics 16, 31. 26044654 10.1186/s12864-015-1606-1 PMC4455334

[age12752-bib-0023] Fariello M.‐I. , Boitard S. , Naya H. , San Cristobal M. & Servin B. (2013) Detecting signatures of selection through haplotype differentiation among hierarchically structured populations. Genetics 193, 929–41. 23307896 10.1534/genetics.112.147231 PMC3584007

[age12752-bib-0024] Fariello M.‐I. , Servin B. , Tosser‐Klopp G. , Rupp R. , Moreno C. , Consortium I.S.G. , SanCristobal M. & Boitard S. (2014) Selection signatures in worldwide sheep populations. PLoS ONE 9, e103813. 25126940 10.1371/journal.pone.0103813 PMC4134316

[age12752-bib-0025] Goudet J. (2005) hierfstat, a package for R to compute and test hierarchical *F*‐statistics. Molecular Ecology Notes 5, 184–6.

[age12752-bib-0026] Grosclaude F. , Mahé M.F. , Brignon G. , Di Stasio L. & Jeunet R. (1987) A Mendelian polymorphism underlying quantitative variations of goat *α*(s1)‐casein. Genetics Selection Evolution 19, 399–412. 10.1186/1297-9686-19-4-399 PMC2713294 22879295

[age12752-bib-0027] Le Jaouen J.C.L. (2002) Les grandes étapes de la mutation de l’élevage caprin en France au XXème siècle In: Ethnozootechnie, La Chèvre: Son Rôle dans la Société au XXème Siècle 70 (Ed. by Société d'Ethnozootechnie ), pp. 3–10. Société d'Ethnozootechnie, Clermont‐Ferrand, France.

[age12752-bib-0028] Lenstra J.A. , Tigchelaar J. , Biebach I. *et al* (2016) Microsatellite diversity of the Nordic type of goats in relation to breed conservation: how relevant is pure ancestry?. Journal of Animal Breeding and Genetics 134, 78–84. 27339108 10.1111/jbg.12226

[age12752-bib-0029] Manfredi E. & Adnøy T. (2012) Génétique des caprins laitiers. INRA Productions Animales 25, 233–44.

[age12752-bib-0030] Martin P.M. , Palhière I. , Ricard A. , Tosser‐Klopp G. & Rupp R. (2016) Genome wide association study identifies new loci associated with undesired coat color phenotypes in Saanen goats. PLoS ONE 11, e0152426. 27030980 10.1371/journal.pone.0152426 PMC4816504

[age12752-bib-0031] Mauron M. (1947) Scènes de la Vie des Bêtes: La Chèvre, ce Caprice Vivant. Albin Michel, Paris.

[age12752-bib-0032] Pickrell J.K. & Pritchard J.K. (2012) Inference of population splits and mixtures from genome‐wide allele frequency data. PLoS Genetics 8, e1002967. 23166502 10.1371/journal.pgen.1002967 PMC3499260

[age12752-bib-0033] Purcell S. , Neale B. , Todd‐Brown K. *et al* (2007) plink: a tool set for whole‐genome association and population‐based linkage analyses. American Journal of Human Genetics 81, 559–75. 17701901 10.1086/519795 PMC1950838

[age12752-bib-0034] Rao C. , Foernzler D. , Loftus S.K. , Liu S. , McPherson J.D. , Jungers K.A. , Apte S.S. , Pavan W.J. & Beier D.R. (2003) A defect in a novel ADAMTS family member is the cause of the belted white‐spotting mutation. Development 130, 4665–72. 12925592 10.1242/dev.00668

[age12752-bib-0035] Redies C. , Hertel N. & Hübner C.A. (2012) Cadherins and neuropsychiatric disorders. Brain Research 1470, 130–44. 22765916 10.1016/j.brainres.2012.06.020

[age12752-bib-0036] Reynolds J. , Weir B.S. & Cockerham C.C. (1983) Estimation of the coancestry coefficient: basis for a short‐term genetic distance. Genetics 105, 767–79. 17246175 10.1093/genetics/105.3.767 PMC1202185

[age12752-bib-0037] Rochus C.M. , Tortereau F. , Plisson‐Petit F. , Restoux G. , Moreno‐Romieux C. , Tosser‐Klopp G. & Servin B. (2018) Revealing the selection history of adaptive loci using genome‐wide scans for selection: an example from domestic sheep. BMC Genomics 19, 71. 29357834 10.1186/s12864-018-4447-x PMC5778797

[age12752-bib-0038] Saitou N. & Nei M. (1987) The neighbor‐joining method: a new method for reconstructing phylogenetic trees. Molecular Biology and Evolution 4, 406–25. 3447015 10.1093/oxfordjournals.molbev.a040454

[age12752-bib-0501] Scheet, P. & Stephens, M. (2006) A fast and flexible statistical model for large‐scale population genotype data: applications to inferring missing genotypes and haplotypic phase, American Journal of Human Genetics, 78, 629–44. 16532393 10.1086/502802 PMC1424677

[age12752-bib-0039] Selvaggi M. , Laudadio V. , Dario C. & Tufarelli V. (2014) Major proteins in goat milk: an updated overview on genetic variability. Molecular Biology Reports 41, 1035–48. 24381104 10.1007/s11033-013-2949-9

[age12752-bib-0040] Silver D.L. , Hou L. , Somerville R. , Young M.E. , Apte S.S. & Pavan W.J. (2008) The secreted metalloprotease ADAMTS20 is required for melanoblast survival. PLoS Genetics 4, 1000003. 10.1371/journal.pgen.1000003 PMC2265537 18454205

[age12752-bib-0041] Spindler F. (1988) L’évolution du cheptel caprin en France. Ethnozootechnie, La Chèvre 41, 113–9.

[age12752-bib-0042] Storey J.D. , Bass A.J. , Dabney A. & Robinson D. (2015) qvalue: Q‐Value Estimation for False Discovery Rate Control. R package version 2.12.0. http://github.com/jdstorey/qvalue.

[age12752-bib-0043] Tosser‐Klopp G. , Bardou P. , Bouchez O. *et al* (2014) Design and characterization of a 52K SNP chip for goats. PLoS ONE 9, e86227. 24465974 10.1371/journal.pone.0086227 PMC3899236

[age12752-bib-0044] Visser C. , Lashmar S.F. , Marle‐Köster E.V. , Poli M.A. & Allain D. (2016) Genetic diversity and population structure in South African, French and Argentinian Angora goats from genome‐wide SNP data. PLoS ONE 11, e0154353. 27171175 10.1371/journal.pone.0154353 PMC4865245

[age12752-bib-0045] Wang K. , Zhang H. , Ma D. *et al* (2009) Common genetic variants on 5p14.1 associate with autism spectrum disorders. Nature 459, 528–33. 19404256 10.1038/nature07999 PMC2943511

[age12752-bib-0046] Weir B.S. & Cockerham C.C. (1984) Estimating *F*‐statistics for the analysis of population structure. Evolution 38, 1358–70. 28563791 10.1111/j.1558-5646.1984.tb05657.x

[age12752-bib-0047] Yang J. , Lee S.H. , Goddard M.E. & Visscher P.M. (2011) gcta: a tool for genome‐wide complex trait analysis. American Journal of Human Genetics 88, 76–82. 21167468 10.1016/j.ajhg.2010.11.011 PMC3014363

[age12752-bib-0048] Zeder M.A. (2008) Domestication and early agriculture in the Mediterranean Basin: origins, diffusion, and impact. Proceedings of the National Academy of Sciences of the United States of America 105, 11597–604. 18697943 10.1073/pnas.0801317105 PMC2575338

