# Supplementary material for: Genetic diversity analysis of French goat populations reveals selective sweeps involved in their differentiation
Source: Anim Genet. 2018 Dec 13;50(1):54–63. doi: 10.1111/age.12752 (PMC6590323; doi:10.1111/age.12752)

Figure S1 Admixture cross-validation procedure. *K* is the number of hypothetical populations evaluated.

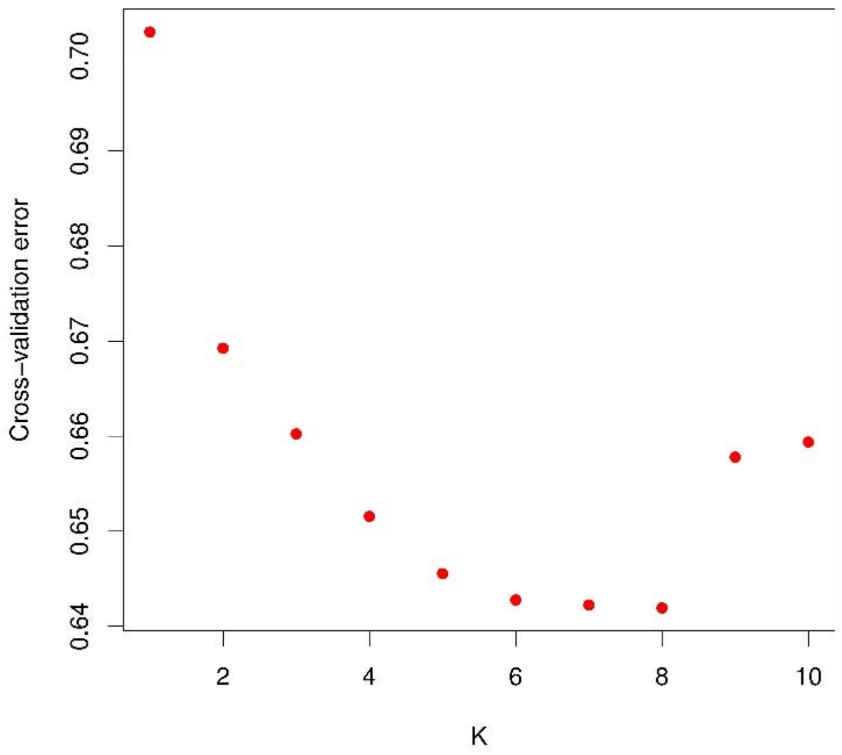

Supplement: Supplementary file 1 — Figure S1 Admixture cross‐validation procedure. [file AGE-50-54-s001.pdf]
