# Supplementary material for: Genetic diversity analysis of French goat populations reveals selective sweeps involved in their differentiation
Source: Anim Genet. 2018 Dec 13;50(1):54–63. doi: 10.1111/age.12752 (PMC6590323; doi:10.1111/age.12752)

Figure S2 Measuring models fit in TREEMIX. m is the number of added migration events, f the fraction of the variance in relatedness between populations that is accounted for by the models.

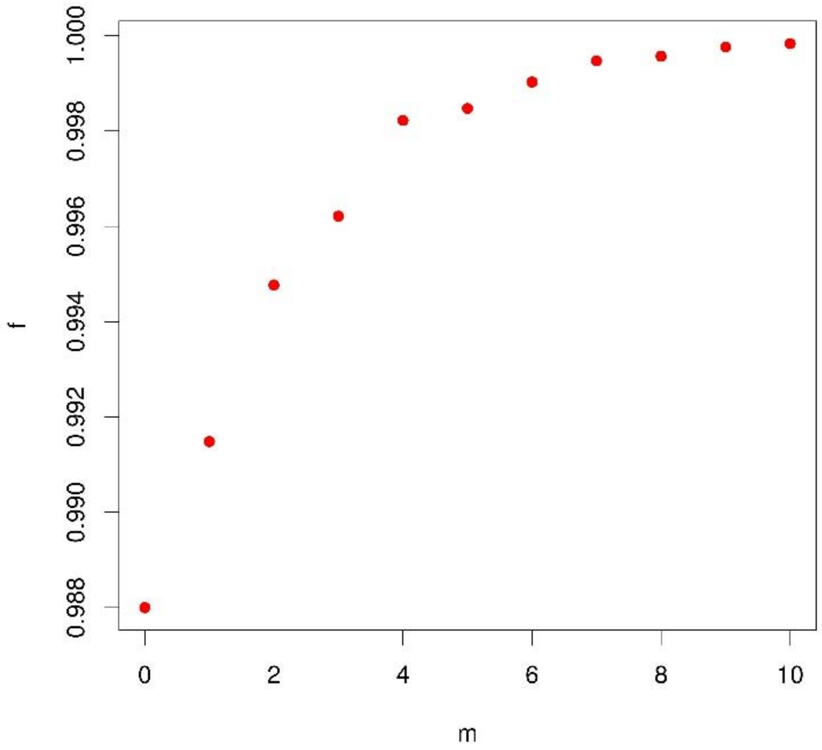

Supplement: Supplementary file 2 — Figure S2 Measuring models fit in treemix. [file AGE-50-54-s002.pdf]
