# Supplementary material for: Genetic diversity analysis of French goat populations reveals selective sweeps involved in their differentiation
Source: Anim Genet. 2018 Dec 13;50(1):54–63. doi: 10.1111/age.12752 (PMC6590323; doi:10.1111/age.12752)

Figure S3 Boxplots of average runs of homozygosity (ROH) lengths (top) and number of ROH for each population.

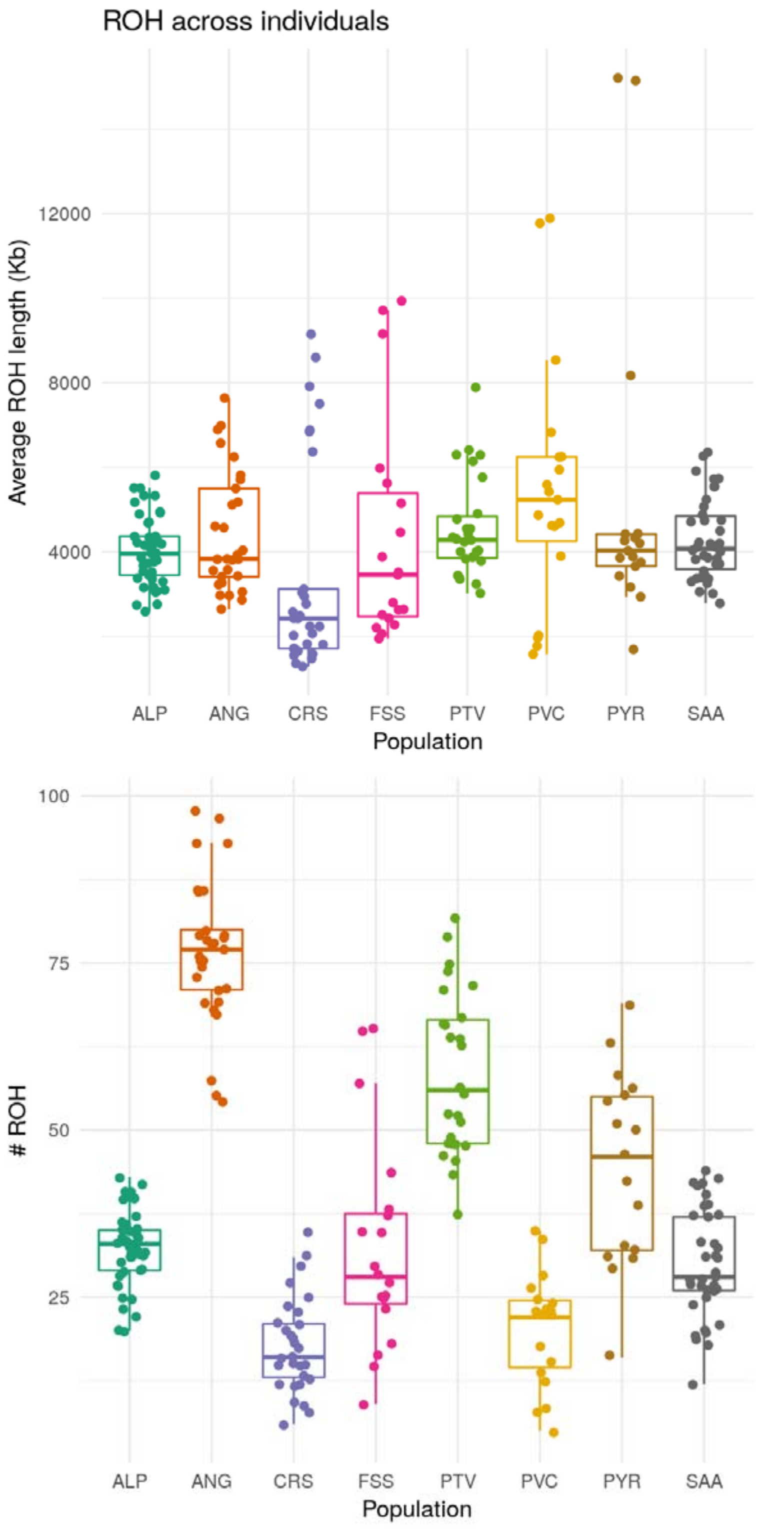

Supplement: Supplementary file 3 — Figure S3 Boxplots of average runs of homozygosity (ROH) lengths and number of ROH for each population. [file AGE-50-54-s003.pdf]
