# Supplementary material for: Genetic diversity analysis of French goat populations reveals selective sweeps involved in their differentiation
Source: Anim Genet. 2018 Dec 13;50(1):54–63. doi: 10.1111/age.12752 (PMC6590323; doi:10.1111/age.12752)

Figure S4 Representation of the first seven principal components of the principal component analysis.

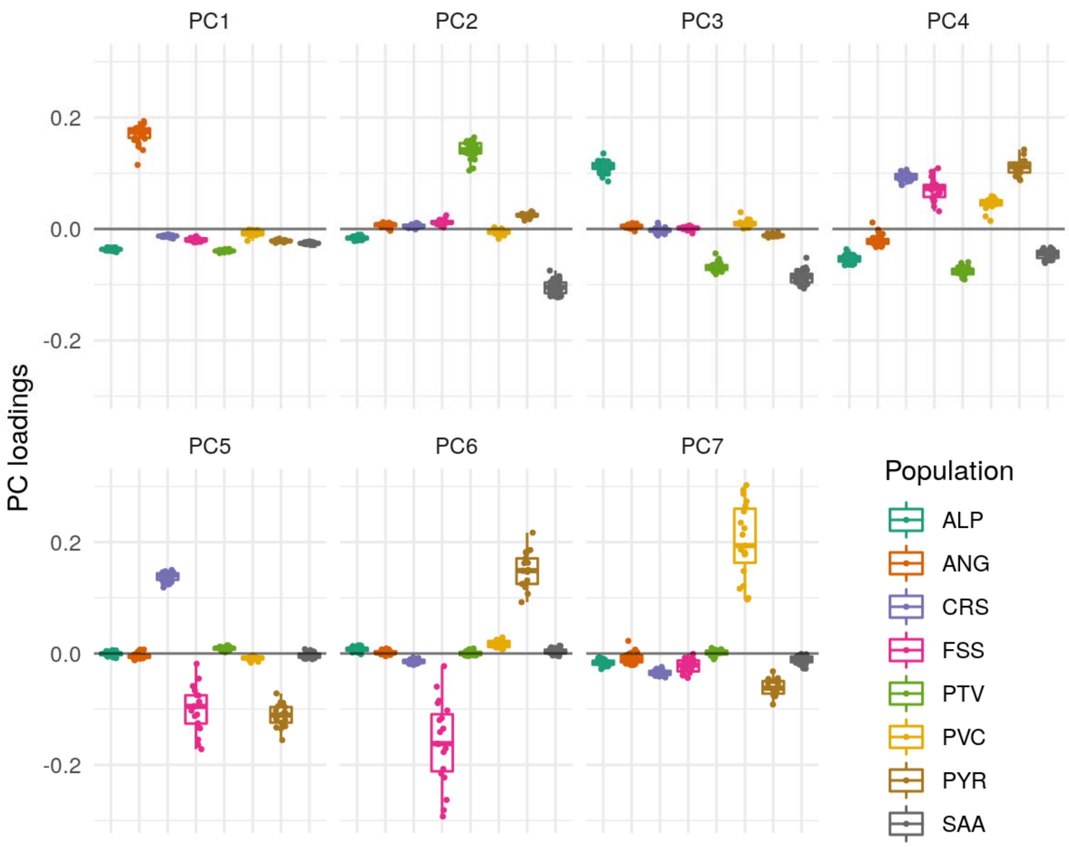

Supplement: Supplementary file 4 — Figure S4 Representation of the first seven principal components of the principal component analysis. [file AGE-50-54-s004.pdf]
