# Supplementary material for: Genetic diversity analysis of French goat populations reveals selective sweeps involved in their differentiation
Source: Anim Genet. 2018 Dec 13;50(1):54–63. doi: 10.1111/age.12752 (PMC6590323; doi:10.1111/age.12752)

Figure S5 Weir & Cockerham F_{ST} values (top, below the diagonal in the table) and Reynold's genetic distances (top, above the diagonal in the table) between each pair of populations. The Reynold's genetic distances were extracted from the population tree (bottom) estimated by the HAPFLK software.

		REYNOLDS							
		**PYR**	**FSS**	**PTV**	**ALP**	**SAA**	**PVC**	**CRS**	**ANG**
**WEIR & COCKERHAM**	**PYR**		0.092	0.111	0.091	0.096	0.091	0.085	0.198
	**FSS**	0.065		0.101	0.076	0.083	0.081	0.075	0.185
	**PTV**	0.089	0.080		0.090	0.100	0.101	0.094	0.212
	**ALP**	0.071	0.058	0.076		0.070	0.070	0.068	0.185
	**SAA**	0.076	0.064	0.085	0.059		0.077	0.073	0.181
	**PVC**	0.064	0.056	0.081	0.053	0.059		0.070	0.168
	**CRS**	0.062	0.054	0.077	0.054	0.059	0.049		0.169
	**ANG**	0.179	0.167	0.198	0.171	0.167	0.151	0.154	

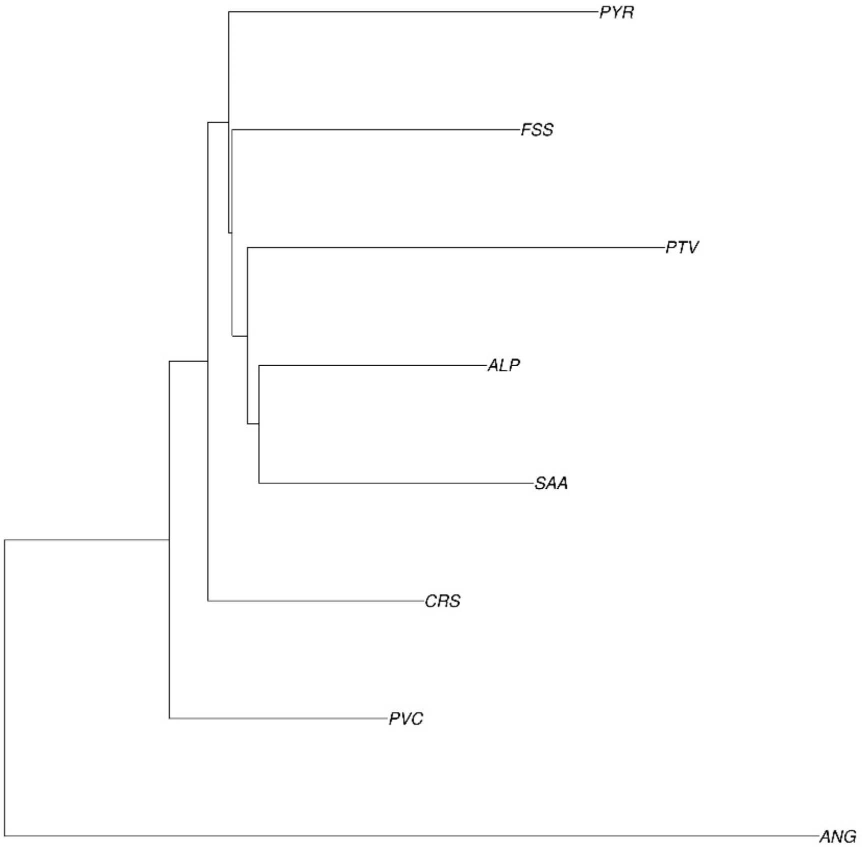

Supplement: Supplementary file 5 — Figure S5 Weir & Cockerham F ST values and Reynold's genetic distances between each pair of populations. [file AGE-50-54-s005.pdf]
