# Supplementary material for: Genetic diversity analysis of French goat populations reveals selective sweeps involved in their differentiation
Source: Anim Genet. 2018 Dec 13;50(1):54–63. doi: 10.1111/age.12752 (PMC6590323; doi:10.1111/age.12752)

Figure S6 Maximum likelihood trees of the selected animals with Iranian goat and Bezoar ibex populations and (a) two, (b) three and (c) four unknown admixture events inferred.

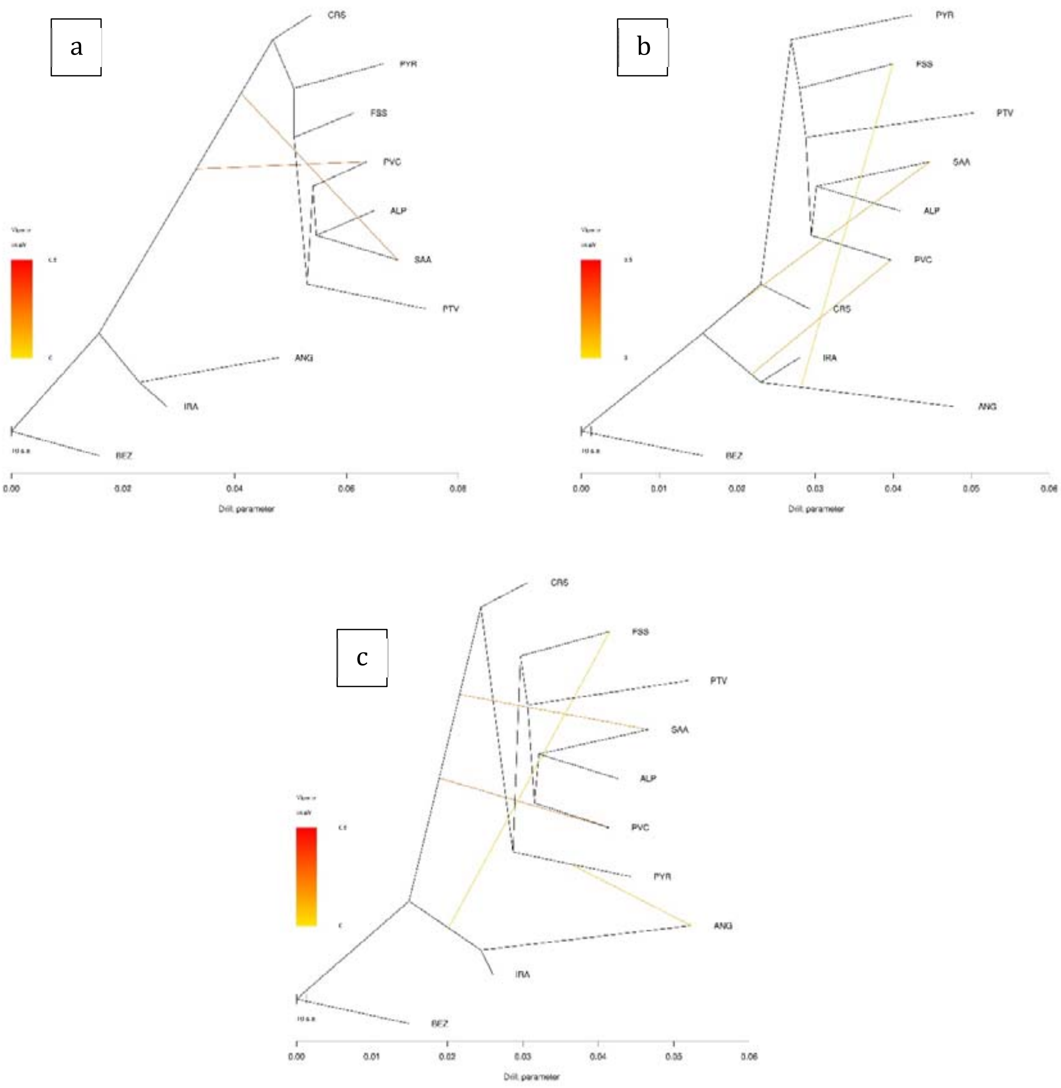

Supplement: Supplementary file 6 — Figure S6 Maximum likelihood trees of the selected animals with Iranian goat and Bezoar ibex populations and (a) two, (b) three and (c) four unknown admixture events inferred. [file AGE-50-54-s006.pdf]
