# Supplementary material for: Genetic diversity analysis of French goat populations reveals selective sweeps involved in their differentiation
Source: Anim Genet. 2018 Dec 13;50(1):54–63. doi: 10.1111/age.12752 (PMC6590323; doi:10.1111/age.12752)

Figure S11 Local population trees (top) computed using the FLK statistic (left) and the hapFLK statistic (right) on the significant region of chromosome 20.The darker a branch color is, the bigger the elongation is compared to the global population tree (computed on the whole genome), which means that a population with a dark branch is differentiated on the local region. The p-values of these statistic tests are provided in the table (bottom left). At the bottom right are represented allele frequencies in each population at each SNP. The two black vertical lines delimit the significant region.

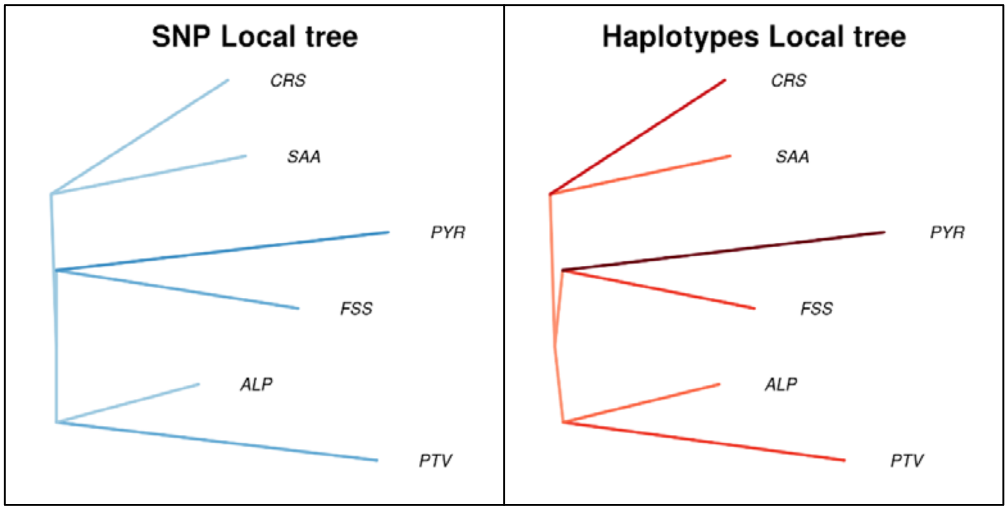

Breed	p-values SNP	p-values Haplotypes
CRS	1.1×10^{-01}	1.9×10^{-04}
SAA	1.5×10^{-01}	6.5×10^{-02}
PYR	1.7×10^{-03}	2.0×10^{-07}
FSS	1.3×10^{-02}	6.4×10^{-03}
ALP	7.7×10^{-01}	6.4×10^{-02}
PTV	1.3×10^{-02}	2.2×10^{-03}

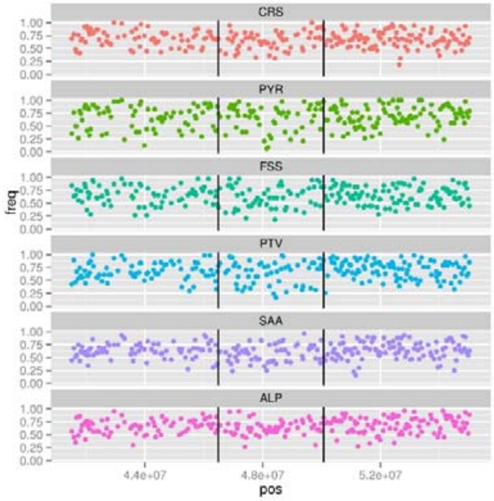

Supplement: Supplementary file 11 — Figure S11 Local population trees computed using the FLK statistic and the hapFLK statistic on the significant region of chromosome 20. [file AGE-50-54-s011.pdf]
