# Supplementary material for: Genetic diversity analysis of French goat populations reveals selective sweeps involved in their differentiation
Source: Anim Genet. 2018 Dec 13;50(1):54–63. doi: 10.1111/age.12752 (PMC6590323; doi:10.1111/age.12752)
Supplement: Supplementary file 12 — Table S1 Allele frequencies in each population at each SNP in the significant regions. [file AGE-50-54-s012.pdf]

**Table S1** Allele frequencies in each population at each SNP in the significant regions.

| rs | chr | pos | all_ref | all_alt | CRS | PYR | FSS | PTV | SAA | ALP |
|---|---|---|---|---|---|---|---|---|---|---|
| snp47327-scaffold665-1059656 | 5 | 34657160 | G | A | 0.57 | 0.38 | 0.58 | 0.46 | 0.43 | 0.49 |
| snp47328-scaffold665-1092095 | 5 | 34684354 | A | G | 0.76 | 0.68 | 0.66 | 0.56 | 0.55 | 0.58 |
| snp47329-scaffold665-1139943 | 5 | 34732611 | A | G | 0.62 | 0.94 | 0.42 | 0.43 | 0.47 | 0.70 |
| snp47330-scaffold665-1180467 | 5 | 34773134 | G | A | 0.66 | 0.53 | 0.89 | 0.89 | 0.66 | 0.90 |
| snp47331-scaffold665-1212741 | 5 | 34805339 | A | C | 0.79 | 0.85 | 0.95 | 0.59 | 0.78 | 0.91 |
| snp47332-scaffold665-1270692 | 5 | 34863212 | A | G | 0.67 | 0.97 | 0.71 | 0.72 | 0.79 | 0.82 |
| snp47334-scaffold665-1367124 | 5 | 34958779 | C | A | 0.59 | 0.65 | 0.55 | 0.72 | 0.83 | 0.64 |
| snp47335-scaffold665-1421492 | 5 | 35013574 | A | G | 0.83 | 0.21 | 0.58 | 0.54 | 0.61 | 0.66 |
| snp47336-scaffold665-1477228 | 5 | 35068920 | A | G | 0.52 | 0.68 | 0.26 | 0.80 | 0.59 | 0.74 |
| snp47337-scaffold665-1515009 | 5 | 35107027 | C | A | 0.45 | 0.65 | 0.58 | 0.59 | 0.72 | 0.60 |
| snp47338-scaffold665-1549358 | 5 | 35141299 | A | G | 0.78 | 0.94 | 0.71 | 0.46 | 0.71 | 0.71 |
| snp47339-scaffold665-1615171 | 5 | 35207630 | A | G | 0.60 | 0.50 | 0.39 | 0.67 | 0.76 | 0.76 |
| snp47340-scaffold665-1648247 | 5 | 35241180 | G | A | 0.97 | 0.47 | 0.84 | 0.78 | 0.64 | 0.83 |
| snp47341-scaffold665-1692641 | 5 | 35285608 | G | A | 0.98 | 0.38 | 0.71 | 0.54 | 0.74 | 0.87 |
| snp47343-scaffold665-1779570 | 5 | 35372348 | A | G | 0.52 | 0.38 | 0.97 | 0.56 | 0.50 | 0.69 |
| snp47344-scaffold665-1878696 | 5 | 35472955 | A | G | 0.71 | 0.32 | 0.66 | 0.76 | 0.59 | 0.89 |
| snp47345-scaffold665-1927526 | 5 | 35522167 | A | G | 0.64 | 0.68 | 0.61 | 0.93 | 0.80 | 0.61 |
| snp47346-scaffold665-1983225 | 5 | 35578154 | A | G | 0.83 | 0.82 | 0.53 | 0.74 | 0.68 | 0.67 |
| snp16394-scaffold1728-163445 | 5 | 35649822 | A | G | 0.62 | 0.94 | 0.66 | 0.76 | 0.75 | 0.59 |
| snp16393-scaffold1728-130761 | 5 | 35682230 | G | A | 0.90 | 1.00 | 0.66 | 0.61 | 0.78 | 0.48 |
| snp16392-scaffold1728-92818 | 5 | 35721015 | A | G | 0.81 | 0.29 | 0.74 | 0.61 | 0.78 | 0.61 |
| snp16391-scaffold1728-62184 | 5 | 35752670 | G | A | 0.52 | 0.82 | 0.58 | 0.33 | 0.33 | 0.56 |
| snp16390-scaffold1728-6620 | 5 | 35806889 | C | A | 0.47 | 0.32 | 0.71 | 0.33 | 0.80 | 0.86 |
| snp9039-scaffold1329-19185 | 5 | 35833383 | G | A | 0.53 | 0.15 | 0.63 | 0.50 | 0.75 | 0.66 |
| snp9040-scaffold1329-60932 | 5 | 35875521 | A | G | 0.72 | 0.97 | 0.87 | 0.85 | 0.92 | 0.94 |
| snp9041-scaffold1329-104354 | 5 | 35918660 | A | G | 0.67 | 0.32 | 0.47 | 0.41 | 0.55 | 0.60 |
| snp9042-scaffold1329-135025 | 5 | 35949345 | A | G | 0.52 | 0.15 | 0.42 | 0.67 | 0.80 | 0.47 |
| snp9043-scaffold1329-183561 | 5 | 35998090 | A | G | 0.97 | 1.00 | 0.95 | 0.89 | 0.78 | 0.83 |
| snp9044-scaffold1329-212363 | 5 | 36027951 | G | A | 0.83 | 0.97 | 0.66 | 0.69 | 0.68 | 0.83 |
| snp9045-scaffold1329-244020 | 5 | 36059565 | G | A | 0.29 | 0.15 | 0.66 | 0.59 | 0.66 | 0.47 |
| snp9046-scaffold1329-282079 | 5 | 36097812 | A | G | 0.50 | 0.24 | 0.50 | 0.81 | 0.67 | 0.46 |
| snp9047-scaffold1329-313566 | 5 | 36127811 | A | G | 0.64 | 0.56 | 0.53 | 0.78 | 0.64 | 0.76 |
| snp9050-scaffold1329-418579 | 5 | 36233025 | A | G | 0.69 | 1.00 | 0.76 | 0.67 | 0.61 | 0.72 |
| snp9051-scaffold1329-454399 | 5 | 36268885 | G | A | 0.53 | 0.97 | 0.37 | 0.67 | 0.42 | 0.49 |
| snp9052-scaffold1329-510083 | 5 | 36325701 | A | G | 0.60 | 0.97 | 0.74 | 0.44 | 0.57 | 0.74 |
| snp9053-scaffold1329-559581 | 5 | 36375339 | G | A | 0.72 | 0.18 | 0.55 | 0.80 | 0.49 | 0.68 |
| snp9056-scaffold1329-675643 | 5 | 36494259 | G | A | 0.76 | 0.94 | 0.84 | 0.50 | 0.63 | 0.80 |
| snp9057-scaffold1329-713250 | 5 | 36529333 | G | A | 0.76 | 0.09 | 0.47 | 0.15 | 0.70 | 0.56 |
| snp9062-scaffold1329-936768 | 5 | 36750654 | A | G | 0.66 | 0.94 | 0.53 | 0.69 | 0.34 | 0.62 |
| snp9063-scaffold1329-976103 | 5 | 36790914 | G | A | 0.69 | 0.18 | 0.18 | 0.72 | 0.55 | 0.64 |

| | | | | | | | | | | |
|---|---|---|---|---|---|---|---|---|---|---|
| snp9064-scaffold1329-1026514 | 5 | 36842273 | A | G | 0.53 | 0.97 | 0.53 | 0.43 | 0.50 | 0.49 |
| snp9065-scaffold1329-1085677 | 5 | 36896097 | A | C | 0.64 | 0.97 | 0.45 | 0.67 | 0.82 | 0.69 |
| snp9066-scaffold1329-1128684 | 5 | 36939084 | A | G | 0.90 | 0.18 | 0.68 | 0.74 | 0.96 | 0.97 |
| snp9067-scaffold1329-1169662 | 5 | 36980654 | G | A | 0.98 | 0.97 | 0.82 | 0.91 | 0.83 | 0.84 |
| snp26896-scaffold2844-2833 | 5 | 37031246 | A | G | 0.59 | 0.91 | 0.71 | 0.52 | 0.46 | 0.62 |
| snp12728-scaffold149-35530 | 5 | 37074733 | A | G | 0.91 | 0.12 | 0.76 | 0.92 | 0.97 | 0.81 |
| snp12729-scaffold149-70593 | 5 | 37109878 | G | A | 0.79 | 0.91 | 0.66 | 0.43 | 0.55 | 0.51 |
| snp12730-scaffold149-99353 | 5 | 37138597 | A | G | 0.60 | 0.94 | 0.64 | 0.69 | 0.78 | 0.71 |
| snp12731-scaffold149-139094 | 5 | 37179334 | A | G | 0.79 | 1.00 | 0.61 | 0.98 | 0.84 | 0.76 |
| snp12732-scaffold149-177542 | 5 | 37217859 | C | A | 0.95 | 0.94 | 1.00 | 1.00 | 0.82 | 0.86 |
| snp12733-scaffold149-215097 | 5 | 37256366 | A | G | 0.72 | 0.15 | 0.97 | 1.00 | 0.89 | 0.83 |
| snp12734-scaffold149-262013 | 5 | 37304425 | G | A | 0.59 | 0.82 | 0.47 | 0.35 | 0.42 | 0.39 |
| snp12735-scaffold149-294409 | 5 | 37340395 | A | G | 0.78 | 0.97 | 0.95 | 1.00 | 0.84 | 0.97 |
| snp12736-scaffold149-347281 | 5 | 37394523 | G | A | 0.48 | 0.21 | 0.68 | 0.52 | 0.89 | 0.58 |
| snp12738-scaffold149-435094 | 5 | 37482107 | C | A | 0.72 | 0.24 | 0.61 | 0.87 | 0.68 | 0.83 |
| snp12740-scaffold149-513479 | 5 | 37559183 | G | A | 0.22 | 0.21 | 0.55 | 0.80 | 0.46 | 0.86 |
| snp12741-scaffold149-563468 | 5 | 37609191 | G | A | 0.90 | 0.21 | 0.87 | 0.67 | 0.72 | 0.82 |
| snp12742-scaffold149-604458 | 5 | 37650200 | A | G | 0.64 | 0.32 | 0.89 | 0.72 | 0.72 | 0.58 |
| snp12743-scaffold149-640801 | 5 | 37687065 | G | A | 0.66 | 0.41 | 0.76 | 0.61 | 0.53 | 0.66 |
| snp12744-scaffold149-712399 | 5 | 37759311 | A | G | 0.74 | 0.79 | 0.84 | 0.85 | 0.92 | 0.84 |
| snp12745-scaffold149-803866 | 5 | 37850749 | A | C | 0.59 | 0.79 | 0.66 | 0.65 | 0.51 | 0.58 |
| snp12746-scaffold149-854352 | 5 | 37900985 | G | A | 0.69 | 0.26 | 0.53 | 0.72 | 0.58 | 0.36 |
| snp12747-scaffold149-909589 | 5 | 37957580 | A | G | 0.55 | 0.74 | 0.61 | 0.61 | 0.70 | 0.50 |
| snp12748-scaffold149-942439 | 5 | 37990479 | G | A | 0.71 | 0.26 | 0.58 | 0.76 | 0.78 | 0.62 |
| snp12749-scaffold149-976393 | 5 | 38024648 | G | A | 0.81 | 1.00 | 0.92 | 0.69 | 0.86 | 0.99 |
| snp12750-scaffold149-1005378 | 5 | 38053632 | G | A | 0.60 | 0.85 | 0.76 | 0.61 | 0.70 | 0.66 |
| snp12753-scaffold149-1131695 | 5 | 38181505 | A | G | 0.69 | 0.85 | 0.79 | 0.72 | 0.68 | 0.78 |
| snp12754-scaffold149-1186113 | 5 | 38237038 | A | G | 0.52 | 0.44 | 0.87 | 0.39 | 0.66 | 0.62 |
| snp12755-scaffold149-1232069 | 5 | 38282676 | G | A | 0.71 | 0.32 | 0.84 | 0.77 | 0.55 | 0.69 |
| snp12756-scaffold149-1263688 | 5 | 38314749 | A | C | 0.43 | 0.32 | 0.63 | 0.50 | 0.66 | 0.47 |
| snp12757-scaffold149-1353154 | 5 | 38405635 | G | A | 0.71 | 0.62 | 0.47 | 0.57 | 0.50 | 0.63 |
| snp12758-scaffold149-1420103 | 5 | 38473200 | G | A | 0.91 | 0.71 | 0.63 | 0.74 | 0.89 | 0.71 |
| snp12759-scaffold149-1454645 | 5 | 38507659 | A | G | 0.74 | 0.41 | 0.58 | 0.70 | 0.86 | 0.60 |
| snp12760-scaffold149-1501840 | 5 | 38555334 | G | A | 0.48 | 0.44 | 0.71 | 0.59 | 0.53 | 0.77 |
| snp12761-scaffold149-1559579 | 5 | 38613742 | A | G | 0.84 | 0.50 | 0.82 | 0.80 | 0.66 | 0.70 |
| snp12762-scaffold149-1606790 | 5 | 38661449 | G | A | 0.91 | 0.35 | 0.47 | 0.63 | 0.55 | 0.86 |
| snp12763-scaffold149-1642005 | 5 | 38697559 | G | A | 0.43 | 0.76 | 0.42 | 0.56 | 0.36 | 0.74 |
| snp12764-scaffold149-1689060 | 5 | 38745455 | G | A | 0.41 | 0.76 | 0.68 | 0.59 | 0.74 | 0.38 |
| snp12765-scaffold149-1733785 | 5 | 38790386 | G | A | 0.84 | 0.88 | 0.53 | 0.56 | 0.43 | 0.64 |
| snp12766-scaffold149-1769401 | 5 | 38826423 | G | A | 0.36 | 0.32 | 0.34 | 0.78 | 0.74 | 0.58 |
| snp12767-scaffold149-1802042 | 5 | 38859095 | G | A | 0.64 | 0.85 | 0.42 | 0.48 | 0.50 | 0.68 |
| snp12768-scaffold149-1830916 | 5 | 38887930 | A | G | 0.78 | 0.76 | 0.79 | 0.78 | 0.78 | 0.71 |
| snp12769-scaffold149-1861621 | 5 | 38918969 | A | G | 0.79 | 0.73 | 0.84 | 0.87 | 0.57 | 0.82 |

| | | | | | | | | | | |
|---|---|---|---|---|---|---|---|---|---|---|
| snp12770-scaffold149-1899629 | 5 | 38957154 | C | A | 0.48 | 0.35 | 0.18 | 0.59 | 0.54 | 0.73 |
| snp12771-scaffold149-1929435 | 5 | 38986925 | G | A | 0.72 | 0.74 | 0.76 | 0.83 | 0.72 | 0.86 |
| snp12772-scaffold149-1973260 | 5 | 39030999 | G | A | 0.71 | 0.68 | 0.87 | 0.72 | 0.71 | 0.81 |
| snp12773-scaffold149-2005010 | 5 | 39063039 | G | A | 0.79 | 0.82 | 0.71 | 0.87 | 0.82 | 0.96 |
| snp12775-scaffold149-2083221 | 5 | 39142499 | G | A | 0.40 | 0.50 | 0.66 | 0.61 | 0.42 | 0.56 |
| snp12777-scaffold149-2157495 | 5 | 39216961 | G | A | 0.72 | 0.35 | 0.71 | 0.74 | 0.84 | 0.68 |
| snp12778-scaffold149-2202933 | 5 | 39262766 | A | G | 0.67 | 0.68 | 0.68 | 0.59 | 0.62 | 0.78 |
| snp12779-scaffold149-2247518 | 5 | 39306963 | C | A | 0.69 | 0.91 | 0.50 | 0.52 | 0.53 | 0.72 |
| snp12780-scaffold149-2299489 | 5 | 39359453 | G | A | 0.52 | 0.32 | 0.76 | 0.65 | 0.49 | 0.66 |
| snp12782-scaffold149-2393437 | 5 | 39453455 | G | A | 0.57 | 0.68 | 0.76 | 0.69 | 0.57 | 0.22 |
| snp12783-scaffold149-2431241 | 5 | 39492036 | G | A | 0.78 | 0.91 | 0.66 | 0.87 | 0.41 | 0.70 |
| snp12785-scaffold149-2564265 | 5 | 39626746 | A | G | 0.59 | 0.50 | 0.89 | 0.70 | 0.54 | 0.51 |
| snp12786-scaffold149-2596451 | 5 | 39658953 | A | G | 0.72 | 0.47 | 0.50 | 0.80 | 0.36 | 0.79 |
| snp12787-scaffold149-2640186 | 5 | 39697174 | G | A | 0.62 | 0.15 | 0.45 | 0.70 | 0.64 | 0.56 |
| snp12788-scaffold149-2676466 | 5 | 39733491 | A | G | 0.74 | 0.41 | 0.82 | 0.94 | 0.78 | 0.58 |
| snp12789-scaffold149-2742417 | 5 | 39800450 | G | A | 0.45 | 0.35 | 0.68 | 0.70 | 0.50 | 0.66 |
| snp5577-scaffold1195-1813787 | 5 | 40229126 | C | A | 0.55 | 0.62 | 0.61 | 0.80 | 0.71 | 0.74 |
| snp5572-scaffold1195-1636091 | 5 | 40409677 | A | G | 0.36 | 0.50 | 0.79 | 0.72 | 0.68 | 0.60 |
| snp5571-scaffold1195-1603944 | 5 | 40440965 | G | A | 0.43 | 0.21 | 0.39 | 0.59 | 0.62 | 0.78 |
| snp5561-scaffold1195-1172626 | 5 | 40871355 | A | G | 0.59 | 0.82 | 0.24 | 0.81 | 0.74 | 0.93 |
| snp5560-scaffold1195-1131719 | 5 | 40912958 | A | C | 0.53 | 0.79 | 0.24 | 0.81 | 0.74 | 0.76 |
| snp5555-scaffold1195-841188 | 5 | 41204720 | G | A | 0.59 | 0.68 | 0.55 | 0.33 | 0.47 | 0.46 |
| snp5548-scaffold1195-532765 | 5 | 41513932 | A | G | 0.50 | 0.65 | 0.32 | 0.41 | 0.37 | 0.68 |
| snp5546-scaffold1195-428889 | 5 | 41619168 | G | A | 0.53 | 0.26 | 0.68 | 0.50 | 0.37 | 0.58 |
| snp5544-scaffold1195-354858 | 5 | 41692510 | A | C | 0.66 | 0.74 | 0.55 | 0.74 | 0.50 | 0.62 |
| snp5540-scaffold1195-169549 | 5 | 41881428 | G | A | 0.66 | 0.44 | 0.61 | 0.85 | 0.88 | 0.92 |
| snp5539-scaffold1195-140210 | 5 | 41910887 | A | G | 0.78 | 0.65 | 0.45 | 0.35 | 0.43 | 0.47 |
| snp5538-scaffold1195-78873 | 5 | 41972975 | A | G | 0.34 | 0.21 | 0.55 | 0.54 | 0.51 | 0.70 |
| snp5537-scaffold1195-37628 | 5 | 42019896 | G | A | 0.48 | 0.50 | 0.79 | 0.41 | 0.62 | 0.29 |
| snp5536-scaffold1195-7510 | 5 | 42050604 | G | A | 0.72 | 0.56 | 0.53 | 0.78 | 0.57 | 0.74 |
| snp57564-scaffold920-14682 | 5 | 42073453 | A | G | 0.71 | 0.56 | 1.00 | 0.94 | 0.84 | 0.90 |
| snp57565-scaffold920-49844 | 5 | 42108666 | A | G | 0.52 | 0.85 | 0.24 | 0.76 | 0.79 | 0.83 |
| snp57567-scaffold920-124704 | 5 | 42183801 | A | G | 0.64 | 0.82 | 0.61 | 0.80 | 0.70 | 0.68 |
| snp57568-scaffold920-170273 | 5 | 42229207 | G | A | 0.84 | 0.91 | 0.63 | 0.83 | 0.80 | 0.74 |
| snp57569-scaffold920-203637 | 5 | 42262450 | A | G | 0.62 | 0.59 | 0.63 | 0.57 | 0.70 | 0.67 |
| snp57570-scaffold920-233027 | 5 | 42292004 | C | A | 0.52 | 0.38 | 0.63 | 0.63 | 0.78 | 0.57 |
| snp57571-scaffold920-266583 | 5 | 42325487 | A | G | 0.62 | 0.24 | 0.68 | 0.74 | 0.87 | 0.68 |
| snp57572-scaffold920-314791 | 5 | 42374355 | A | G | 0.62 | 0.88 | 0.82 | 0.41 | 0.55 | 0.66 |
| snp57573-scaffold920-345825 | 5 | 42405631 | G | A | 0.76 | 0.85 | 0.92 | 0.74 | 0.86 | 0.70 |
| snp57574-scaffold920-386944 | 5 | 42446710 | A | G | 0.52 | 0.71 | 0.84 | 0.48 | 0.63 | 0.68 |
| snp57575-scaffold920-434644 | 5 | 42494355 | G | A | 0.72 | 0.94 | 0.55 | 0.76 | 0.41 | 0.51 |
| snp57576-scaffold920-486415 | 5 | 42546039 | A | G | 0.43 | 0.38 | 0.26 | 0.76 | 0.68 | 0.61 |
| snp33525-scaffold394-26343 | 5 | 42592303 | A | G | 0.55 | 0.59 | 0.29 | 0.46 | 0.50 | 0.57 |

| | | | | | | | | | | |
|---|---|---|---|---|---|---|---|---|---|---|
| snp33526-scaffold394-54908 | 5 | 42621052 | A | G | 0.62 | 0.68 | 0.29 | 0.39 | 0.57 | 0.33 |
| snp33528-scaffold394-148632 | 5 | 42715735 | A | G | 0.45 | 0.65 | 0.84 | 0.78 | 0.62 | 0.61 |
| snp33530-scaffold394-244108 | 5 | 42811949 | A | G | 0.67 | 0.56 | 0.84 | 0.57 | 0.70 | 0.70 |
| snp33531-scaffold394-285901 | 5 | 42854051 | G | A | 0.90 | 0.85 | 1.00 | 0.98 | 0.92 | 0.97 |
| snp33532-scaffold394-314548 | 5 | 42882728 | A | G | 0.66 | 0.56 | 0.87 | 0.72 | 0.46 | 0.60 |
| snp33533-scaffold394-368389 | 5 | 42936139 | G | A | 0.81 | 0.71 | 0.47 | 0.65 | 0.66 | 0.76 |
| snp33534-scaffold394-434919 | 5 | 42994673 | A | G | 0.63 | 0.71 | 0.61 | 0.56 | 0.79 | 0.77 |
| snp33535-scaffold394-475059 | 5 | 43034728 | A | G | 0.19 | 0.74 | 0.68 | 0.46 | 0.55 | 0.53 |
| snp33537-scaffold394-564974 | 5 | 43125506 | A | G | 0.62 | 0.59 | 0.63 | 0.72 | 0.62 | 0.57 |
| snp33538-scaffold394-599027 | 5 | 43159828 | G | A | 0.72 | 0.88 | 0.63 | 0.93 | 0.79 | 0.70 |
| snp33539-scaffold394-641847 | 5 | 43205205 | G | A | 0.43 | 0.71 | 0.50 | 0.63 | 0.49 | 0.33 |
| snp33540-scaffold394-688646 | 5 | 43252102 | A | C | 0.64 | 0.71 | 0.82 | 0.63 | 0.41 | 0.48 |
| snp33541-scaffold394-725913 | 5 | 43289651 | A | G | 0.67 | 0.94 | 0.71 | 0.65 | 0.64 | 0.43 |
| snp33542-scaffold394-770559 | 5 | 43334604 | A | G | 0.57 | 0.35 | 0.47 | 0.52 | 0.82 | 0.33 |
| snp33543-scaffold394-802791 | 5 | 43366871 | G | A | 0.62 | 0.79 | 0.55 | 0.72 | 0.87 | 0.73 |
| snp33546-scaffold394-896533 | 5 | 43460424 | G | A | 0.67 | 0.94 | 0.74 | 0.91 | 0.68 | 0.76 |
| snp33547-scaffold394-931713 | 5 | 43495960 | G | A | 0.38 | 0.68 | 0.68 | 0.91 | 0.62 | 0.38 |
| snp33548-scaffold394-966334 | 5 | 43530475 | G | A | 0.76 | 0.94 | 0.66 | 0.52 | 0.87 | 0.83 |
| snp33549-scaffold394-1020771 | 5 | 43586176 | G | A | 0.67 | 0.76 | 0.71 | 0.83 | 0.57 | 0.61 |
| snp33550-scaffold394-1082301 | 5 | 43649515 | A | G | 0.72 | 0.74 | 0.97 | 0.57 | 0.86 | 0.90 |
| snp33551-scaffold394-1139564 | 5 | 43706864 | C | A | 0.47 | 0.32 | 0.84 | 0.52 | 0.88 | 0.63 |
| snp33552-scaffold394-1173557 | 5 | 43741812 | G | A | 0.55 | 0.56 | 0.53 | 0.57 | 0.72 | 0.83 |
| snp33553-scaffold394-1206836 | 5 | 43775395 | A | C | 0.74 | 0.76 | 0.68 | 0.67 | 0.37 | 0.66 |
| snp33554-scaffold394-1268017 | 5 | 43836970 | A | C | 0.72 | 0.62 | 0.42 | 0.50 | 0.42 | 0.66 |
| snp33555-scaffold394-1335362 | 5 | 43904817 | A | G | 0.66 | 0.82 | 0.74 | 0.46 | 0.83 | 0.58 |
| snp33556-scaffold394-1391886 | 5 | 43961620 | G | A | 0.66 | 0.88 | 0.55 | 0.57 | 0.34 | 0.61 |
| snp33557-scaffold394-1421320 | 5 | 43991190 | A | G | 0.48 | 0.71 | 0.82 | 0.65 | 0.71 | 0.78 |
| snp33558-scaffold394-1477333 | 5 | 44047930 | A | G | 0.74 | 0.56 | 0.66 | 0.72 | 0.68 | 0.57 |
| snp33559-scaffold394-1535623 | 5 | 44105912 | G | A | 0.93 | 0.88 | 0.50 | 0.67 | 0.75 | 0.71 |
| snp33560-scaffold394-1567427 | 5 | 44136024 | G | A | 0.79 | 0.53 | 0.58 | 0.59 | 0.75 | 0.59 |
| snp33561-scaffold394-1598541 | 5 | 44167423 | A | G | 0.43 | 0.50 | 0.42 | 0.69 | 0.36 | 0.42 |
| snp33562-scaffold394-1629149 | 5 | 44198081 | A | C | 0.72 | 0.71 | 0.74 | 0.59 | 0.53 | 0.61 |
| snp33563-scaffold394-1671628 | 5 | 44240563 | A | G | 0.81 | 0.88 | 0.74 | 0.54 | 0.66 | 0.97 |
| snp33564-scaffold394-1726859 | 5 | 44296293 | A | C | 0.71 | 0.97 | 0.76 | 0.67 | 0.75 | 0.54 |
| snp33565-scaffold394-1761309 | 5 | 44330727 | A | G | 0.72 | 0.68 | 0.42 | 0.56 | 0.34 | 0.43 |
| snp33566-scaffold394-1793546 | 5 | 44362966 | A | G | 0.29 | 0.59 | 0.50 | 0.50 | 0.66 | 0.57 |
| snp33567-scaffold394-1843275 | 5 | 44412991 | G | A | 0.90 | 0.47 | 0.82 | 0.65 | 0.66 | 0.68 |
| snp33568-scaffold394-1880131 | 5 | 44450304 | G | A | 0.57 | 0.68 | 0.76 | 0.67 | 0.50 | 0.74 |
| snp33569-scaffold394-1918346 | 5 | 44489192 | A | G | 0.48 | 0.91 | 0.68 | 0.89 | 0.57 | 0.77 |
| snp33570-scaffold394-1954159 | 5 | 44525279 | A | G | 0.95 | 0.94 | 1.00 | 0.94 | 0.86 | 0.61 |
| snp33571-scaffold394-1990500 | 5 | 44561354 | A | G | 0.41 | 0.68 | 0.76 | 0.57 | 0.49 | 0.72 |
| snp33572-scaffold394-2026538 | 5 | 44597364 | G | A | 0.64 | 0.68 | 0.79 | 0.61 | 0.45 | 0.67 |
| snp33573-scaffold394-2069623 | 5 | 44640719 | A | G | 0.38 | 0.56 | 0.50 | 0.59 | 0.47 | 0.66 |

| | | | | | | | | | | |
|---|---|---|---|---|---|---|---|---|---|---|
| snp33574-scaffold394-2105639 | 5 | 44677030 | A | G | 0.47 | 0.59 | 0.50 | 0.81 | 0.62 | 0.40 |
| snp33575-scaffold394-2136356 | 5 | 44707874 | A | C | 0.47 | 0.62 | 0.66 | 0.61 | 0.64 | 0.44 |
| snp33576-scaffold394-2186249 | 5 | 44757700 | A | G | 0.64 | 0.32 | 0.39 | 0.59 | 0.68 | 0.37 |
| snp33577-scaffold394-2231546 | 5 | 44802335 | A | G | 0.38 | 0.82 | 0.74 | 0.48 | 0.42 | 0.66 |
| snp33578-scaffold394-2273305 | 5 | 44844645 | A | G | 0.69 | 0.62 | 0.79 | 0.74 | 0.43 | 0.60 |
| snp33579-scaffold394-2311866 | 5 | 44882293 | A | G | 0.52 | 0.71 | 0.53 | 0.48 | 0.45 | 0.86 |
| snp33580-scaffold394-2361728 | 5 | 44932677 | G | A | 0.73 | 0.41 | 0.66 | 0.56 | 0.36 | 0.44 |
| snp33581-scaffold394-2393158 | 5 | 44964554 | G | A | 0.57 | 0.53 | 0.58 | 0.61 | 0.36 | 0.63 |
| snp33582-scaffold394-2428482 | 5 | 45000129 | G | A | 0.48 | 0.65 | 0.21 | 0.39 | 0.67 | 0.57 |
| snp33583-scaffold394-2473852 | 5 | 45046543 | G | A | 0.47 | 0.41 | 0.18 | 0.83 | 0.66 | 0.67 |
| snp33584-scaffold394-2504593 | 5 | 45077120 | G | A | 0.55 | 0.62 | 0.26 | 0.83 | 0.66 | 0.87 |
| snp33585-scaffold394-2534529 | 5 | 45106560 | A | G | 0.57 | 0.41 | 0.50 | 0.52 | 0.43 | 0.46 |
| snp33586-scaffold394-2591423 | 5 | 45163607 | G | A | 0.62 | 0.71 | 0.71 | 0.65 | 0.57 | 0.68 |
| snp33587-scaffold394-2648663 | 5 | 45221370 | A | G | 0.79 | 0.65 | 0.71 | 0.59 | 0.42 | 0.69 |
| snp33590-scaffold394-2779676 | 5 | 45351364 | G | A | 0.76 | 0.21 | 0.47 | 0.54 | 0.26 | 0.74 |
| snp33591-scaffold394-2817260 | 5 | 45388715 | A | G | 0.76 | 0.88 | 0.53 | 0.50 | 0.64 | 0.71 |
| snp33592-scaffold394-2858062 | 5 | 45430264 | A | G | 0.52 | 0.56 | 0.34 | 0.54 | 0.75 | 0.41 |
| snp33594-scaffold394-2924580 | 5 | 45496968 | G | A | 0.59 | 0.76 | 0.71 | 0.80 | 0.47 | 0.76 |
| snp33595-scaffold394-2955482 | 5 | 45527741 | A | G | 0.26 | 0.38 | 0.47 | 0.44 | 0.86 | 0.50 |
| snp33597-scaffold394-3041485 | 5 | 45613940 | A | G | 0.69 | 0.62 | 0.32 | 0.35 | 0.62 | 0.58 |
| snp33599-scaffold394-3104563 | 5 | 45677635 | A | G | 0.62 | 0.82 | 0.97 | 0.83 | 0.75 | 0.56 |
| snp33600-scaffold394-3156465 | 5 | 45728657 | A | G | 0.71 | 0.56 | 0.84 | 0.83 | 0.70 | 0.70 |
| snp33602-scaffold394-3249727 | 5 | 45822377 | C | A | 0.86 | 0.97 | 0.97 | 0.48 | 0.72 | 0.74 |
| snp33603-scaffold394-3280465 | 5 | 45853478 | C | A | 0.97 | 0.97 | 0.82 | 1.00 | 1.00 | 0.94 |
| snp33605-scaffold394-3354784 | 5 | 45928164 | A | G | 0.59 | 0.76 | 0.74 | 0.69 | 0.45 | 0.86 |
| snp33606-scaffold394-3398939 | 5 | 45972655 | A | G | 0.90 | 0.91 | 0.79 | 0.80 | 0.55 | 0.87 |
| snp33607-scaffold394-3428515 | 5 | 46001931 | A | C | 0.69 | 0.68 | 0.34 | 0.61 | 0.57 | 0.68 |
| snp33609-scaffold394-3489400 | 5 | 46062685 | A | C | 0.95 | 0.97 | 0.82 | 0.93 | 0.88 | 0.59 |
| snp33610-scaffold394-3519484 | 5 | 46093066 | C | A | 0.79 | 0.56 | 0.47 | 0.44 | 0.50 | 0.70 |
| snp33611-scaffold394-3559760 | 5 | 46133349 | A | G | 0.64 | 0.41 | 0.39 | 0.31 | 0.45 | 0.67 |
| snp33613-scaffold394-3650425 | 5 | 46224110 | G | A | 0.67 | 0.62 | 0.79 | 0.81 | 0.37 | 0.62 |
| snp33614-scaffold394-3679756 | 5 | 46253354 | A | G | 0.84 | 0.62 | 0.50 | 0.65 | 0.49 | 0.68 |
| snp23603-scaffold2380-11002 | 5 | 46269956 | A | G | 0.57 | 0.53 | 0.82 | 0.54 | 0.32 | 0.50 |
| snp23604-scaffold2380-56406 | 5 | 46315078 | G | A | 0.79 | 0.44 | 0.32 | 0.39 | 0.45 | 0.68 |
| snp23605-scaffold2380-91112 | 5 | 46349638 | G | A | 0.64 | 0.38 | 0.42 | 0.72 | 0.50 | 0.73 |
| snp23606-scaffold2380-136017 | 5 | 46394756 | G | A | 0.69 | 0.82 | 0.34 | 0.52 | 0.78 | 0.70 |
| snp23607-scaffold2380-185632 | 5 | 46443907 | A | G | 0.43 | 0.29 | 0.37 | 0.87 | 0.36 | 0.69 |
| snp23608-scaffold2380-223058 | 5 | 46480581 | A | C | 0.69 | 0.79 | 0.82 | 0.54 | 0.63 | 0.46 |
| snp19619-scaffold1980-809484 | 5 | 46605830 | A | G | 0.79 | 0.62 | 0.55 | 0.63 | 0.76 | 0.50 |
| snp19618-scaffold1980-777113 | 5 | 46638381 | G | A | 0.53 | 0.32 | 0.47 | 0.63 | 0.59 | 0.67 |
| snp19617-scaffold1980-729700 | 5 | 46685578 | A | G | 0.52 | 0.32 | 0.68 | 0.43 | 0.66 | 0.31 |
| snp19616-scaffold1980-679320 | 5 | 46736109 | A | G | 0.53 | 0.47 | 0.37 | 0.56 | 0.74 | 0.73 |
| snp19615-scaffold1980-634136 | 5 | 46781744 | G | A | 0.67 | 0.65 | 0.89 | 0.54 | 0.82 | 0.68 |

| | | | | | | | | | | |
|---|---|---|---|---|---|---|---|---|---|---|
| snp19613-scaffold1980-566705 | 5 | 46849479 | A | G | 0.69 | 0.94 | 0.58 | 0.65 | 0.93 | 0.78 |
| snp19612-scaffold1980-517252 | 5 | 46899620 | G | A | 0.72 | 0.91 | 0.92 | 0.78 | 0.53 | 0.91 |
| snp19611-scaffold1980-487633 | 5 | 46928745 | A | G | 0.57 | 0.65 | 0.58 | 0.61 | 0.80 | 0.88 |
| snp19610-scaffold1980-453812 | 5 | 46962568 | A | G | 0.97 | 1.00 | 0.95 | 0.98 | 0.64 | 0.98 |
| snp19609-scaffold1980-395706 | 5 | 47020710 | G | A | 0.64 | 0.59 | 0.61 | 0.69 | 0.89 | 0.80 |
| snp19608-scaffold1980-359119 | 5 | 47057328 | G | A | 0.48 | 0.53 | 0.68 | 0.30 | 0.66 | 0.31 |
| snp19607-scaffold1980-295327 | 5 | 47121418 | G | A | 0.66 | 0.97 | 0.87 | 0.59 | 0.92 | 0.90 |
| snp19606-scaffold1980-264859 | 5 | 47150732 | G | A | 0.57 | 0.79 | 0.61 | 0.72 | 0.54 | 0.83 |
| snp19605-scaffold1980-225263 | 5 | 47190231 | G | A | 0.93 | 0.97 | 1.00 | 0.96 | 1.00 | 0.90 |
| snp19604-scaffold1980-177701 | 5 | 47237641 | G | A | 0.34 | 0.76 | 0.29 | 0.80 | 0.43 | 0.33 |
| snp19603-scaffold1980-142196 | 5 | 47273128 | A | G | 0.66 | 0.44 | 0.71 | 0.17 | 0.62 | 0.80 |
| snp19602-scaffold1980-96480 | 5 | 47318932 | G | A | 0.81 | 0.91 | 0.89 | 1.00 | 0.46 | 0.49 |
| snp19601-scaffold1980-36455 | 5 | 47379142 | G | A | 0.86 | 0.71 | 0.83 | 0.85 | 0.97 | 0.83 |
| snp37629-scaffold463-5194 | 5 | 47420818 | G | A | 0.90 | 0.79 | 0.87 | 0.85 | 0.99 | 0.88 |
| snp37630-scaffold463-64670 | 5 | 47480356 | G | A | 0.53 | 0.88 | 0.58 | 0.65 | 0.78 | 0.34 |
| snp37631-scaffold463-103795 | 5 | 47519367 | C | A | 0.60 | 0.74 | 0.74 | 0.83 | 0.82 | 0.72 |
| snp37632-scaffold463-182773 | 5 | 47598473 | A | G | 0.67 | 0.76 | 0.71 | 0.67 | 0.61 | 0.73 |
| snp37633-scaffold463-212308 | 5 | 47628373 | G | A | 0.34 | 0.62 | 0.63 | 0.91 | 0.63 | 0.82 |
| snp37635-scaffold463-291493 | 5 | 47708081 | A | G | 0.53 | 0.29 | 0.42 | 0.63 | 0.87 | 0.64 |
| snp37636-scaffold463-349673 | 5 | 47768396 | G | A | 0.78 | 0.91 | 0.34 | 0.91 | 0.78 | 0.87 |
| snp37637-scaffold463-397179 | 5 | 47814935 | G | A | 0.40 | 0.50 | 0.21 | 0.87 | 0.79 | 0.87 |
| snp37639-scaffold463-478192 | 5 | 47892605 | G | A | 0.93 | 0.91 | 0.84 | 0.78 | 0.78 | 1.00 |
| snp37640-scaffold463-531792 | 5 | 47946377 | A | G | 0.57 | 0.47 | 0.63 | 0.57 | 0.76 | 0.63 |
| snp37641-scaffold463-576769 | 5 | 47991210 | G | A | 0.67 | 0.62 | 0.66 | 0.63 | 0.51 | 0.43 |
| snp37642-scaffold463-608712 | 5 | 48023083 | A | C | 0.62 | 0.68 | 0.53 | 0.15 | 0.50 | 0.36 |
| snp37643-scaffold463-637924 | 5 | 48051150 | A | C | 0.43 | 0.94 | 0.68 | 0.78 | 0.64 | 0.56 |
| snp37644-scaffold463-676381 | 5 | 48087791 | A | G | 0.59 | 0.88 | 0.58 | 0.80 | 0.75 | 0.62 |
| snp37645-scaffold463-746757 | 5 | 48158501 | G | A | 0.95 | 0.47 | 0.61 | 0.85 | 0.72 | 0.89 |
| snp37646-scaffold463-791102 | 5 | 48203122 | A | G | 0.62 | 0.56 | 0.50 | 0.80 | 0.49 | 0.52 |
| snp37647-scaffold463-835694 | 5 | 48247742 | G | A | 0.78 | 0.71 | 0.66 | 0.26 | 0.74 | 0.41 |
| snp37648-scaffold463-888124 | 5 | 48300056 | A | C | 0.36 | 0.15 | 0.68 | 0.61 | 0.55 | 0.69 |
| snp37649-scaffold463-925662 | 5 | 48337603 | A | G | 0.48 | 0.79 | 0.39 | 0.43 | 0.57 | 0.32 |
| snp40996-scaffold528-920051 | 5 | 48351889 | G | A | 0.48 | 0.82 | 0.42 | 0.63 | 0.83 | 0.41 |
| snp40995-scaffold528-876435 | 5 | 48395304 | G | A | 0.60 | 0.74 | 0.97 | 0.70 | 0.88 | 0.83 |
| snp40994-scaffold528-832270 | 5 | 48439210 | C | A | 0.81 | 0.97 | 0.74 | 0.67 | 0.57 | 0.83 |
| snp40992-scaffold528-736723 | 5 | 48535037 | A | G | 0.91 | 0.38 | 0.89 | 0.70 | 0.57 | 0.50 |
| snp40990-scaffold528-613480 | 5 | 48658165 | A | G | 0.67 | 0.85 | 0.92 | 0.94 | 0.64 | 0.71 |
| snp40989-scaffold528-565003 | 5 | 48707115 | A | G | 0.72 | 0.82 | 0.55 | 0.89 | 0.29 | 0.67 |
| snp40988-scaffold528-515415 | 5 | 48757052 | G | A | 0.91 | 0.85 | 0.97 | 0.98 | 0.51 | 0.91 |
| snp40987-scaffold528-477537 | 5 | 48795092 | A | G | 0.41 | 0.56 | 0.16 | 0.61 | 0.80 | 0.74 |
| snp40986-scaffold528-422468 | 5 | 48849206 | C | A | 0.67 | 0.41 | 0.61 | 0.74 | 0.92 | 0.87 |
| snp40985-scaffold528-351341 | 5 | 48920373 | A | C | 0.62 | 0.47 | 0.76 | 0.50 | 0.55 | 0.63 |
| snp40984-scaffold528-311242 | 5 | 48960455 | A | G | 0.86 | 0.82 | 0.76 | 0.87 | 0.62 | 0.88 |

| | | | | | | | | | | |
|---|---|---|---|---|---|---|---|---|---|---|
| snp40983-scaffold528-261319 | 5 | 49010452 | G | A | 0.69 | 0.56 | 0.74 | 0.37 | 0.64 | 0.53 |
| snp40981-scaffold528-192048 | 5 | 49080567 | A | G | 0.55 | 0.41 | 0.92 | 0.52 | 0.89 | 0.32 |
| snp40980-scaffold528-138654 | 5 | 49134274 | A | G | 0.41 | 0.79 | 0.55 | 0.48 | 0.82 | 0.36 |
| snp40979-scaffold528-108837 | 5 | 49163873 | G | A | 0.52 | 0.79 | 0.74 | 0.33 | 0.36 | 0.50 |
| snp40978-scaffold528-56922 | 5 | 49216066 | A | G | 0.76 | 0.35 | 0.53 | 0.35 | 0.88 | 0.47 |
| snp40977-scaffold528-25893 | 5 | 49246964 | A | C | 0.50 | 0.71 | 0.53 | 0.87 | 0.16 | 0.62 |
| snp21784-scaffold2136-24732 | 5 | 49297947 | G | A | 0.83 | 0.76 | 0.61 | 0.57 | 0.74 | 0.67 |
| snp52256-scaffold775-15664 | 5 | 49338723 | A | G | 0.69 | 0.68 | 0.26 | 0.58 | 0.74 | 0.43 |
| snp52257-scaffold775-50905 | 5 | 49374478 | G | A | 0.69 | 0.85 | 0.61 | 0.94 | 0.61 | 0.53 |
| snp52258-scaffold775-101304 | 5 | 49425364 | G | A | 0.62 | 0.26 | 0.55 | 0.70 | 0.80 | 0.82 |
| snp52259-scaffold775-162850 | 5 | 49487776 | A | G | 0.40 | 0.59 | 0.58 | 0.56 | 0.66 | 0.49 |
| snp52260-scaffold775-197224 | 5 | 49522372 | A | G | 0.69 | 0.50 | 0.37 | 0.72 | 0.53 | 0.59 |
| snp52261-scaffold775-229181 | 5 | 49555197 | G | A | 0.57 | 0.41 | 0.24 | 0.72 | 0.34 | 0.59 |
| snp52262-scaffold775-259323 | 5 | 49585474 | A | C | 0.62 | 0.44 | 0.89 | 0.43 | 0.79 | 0.44 |
| snp52263-scaffold775-293013 | 5 | 49621986 | G | A | 0.53 | 0.56 | 0.18 | 0.59 | 0.47 | 0.59 |
| snp24495-scaffold248-3261177 | 6 | 79080590 | G | A | 0.60 | 0.88 | 0.74 | 0.74 | 0.49 | 0.31 |
| snp24496-scaffold248-3307835 | 6 | 79128433 | G | A | 0.45 | 0.56 | 0.69 | 0.67 | 0.84 | 0.54 |
| snp24497-scaffold248-3388614 | 6 | 79209758 | A | G | 0.79 | 0.47 | 0.63 | 0.46 | 0.42 | 0.53 |
| snp24500-scaffold248-3560265 | 6 | 79383570 | G | A | 0.40 | 0.32 | 0.42 | 0.85 | 0.49 | 0.54 |
| snp24502-scaffold248-3650093 | 6 | 79474269 | G | A | 0.54 | 0.18 | 0.66 | 0.73 | 0.38 | 0.59 |
| snp24503-scaffold248-3686298 | 6 | 79510543 | C | A | 0.62 | 0.50 | 0.76 | 0.74 | 0.59 | 0.84 |
| snp27941-scaffold30-18900 | 6 | 79560230 | A | G | 0.48 | 0.21 | 0.50 | 0.48 | 0.58 | 0.78 |
| snp27942-scaffold30-57342 | 6 | 79599168 | A | G | 0.62 | 0.24 | 0.55 | 0.46 | 0.78 | 0.81 |
| snp27943-scaffold30-96873 | 6 | 79638999 | G | A | 0.60 | 0.85 | 0.50 | 0.57 | 0.50 | 0.21 |
| snp27947-scaffold30-313949 | 6 | 79902117 | G | A | 0.93 | 0.47 | 0.87 | 0.98 | 0.76 | 0.57 |
| snp27949-scaffold30-396475 | 6 | 79986113 | A | G | 0.60 | 0.35 | 0.71 | 0.78 | 0.93 | 0.26 |
| snp27950-scaffold30-433050 | 6 | 80013178 | G | A | 0.57 | 0.88 | 0.39 | 0.57 | 0.36 | 0.92 |
| snp27951-scaffold30-467775 | 6 | 80047974 | A | G | 0.55 | 0.38 | 0.50 | 0.37 | 0.59 | 0.62 |
| snp27952-scaffold30-513690 | 6 | 80078614 | A | G | 0.67 | 0.56 | 0.53 | 0.61 | 0.47 | 0.87 |
| snp27954-scaffold30-579575 | 6 | 80144119 | A | G | 0.88 | 1.00 | 0.76 | 0.87 | 0.71 | 0.98 |
| snp27955-scaffold30-611495 | 6 | 80176656 | G | A | 0.43 | 0.74 | 0.58 | 0.41 | 0.30 | 0.87 |
| snp27956-scaffold30-656520 | 6 | 80221671 | A | G | 0.88 | 0.91 | 1.00 | 1.00 | 0.86 | 0.63 |
| snp27958-scaffold30-770690 | 6 | 80336372 | G | A | 0.69 | 0.74 | 0.66 | 0.61 | 0.74 | 0.82 |
| snp27959-scaffold30-800185 | 6 | 80364302 | A | G | 0.67 | 0.82 | 0.76 | 0.87 | 0.58 | 0.61 |
| snp27960-scaffold30-829941 | 6 | 80394124 | G | A | 0.64 | 1.00 | 0.95 | 0.67 | 0.87 | 0.59 |
| snp27961-scaffold30-875758 | 6 | 80439696 | A | G | 0.74 | 0.85 | 0.82 | 0.70 | 0.58 | 0.89 |
| snp27962-scaffold30-922676 | 6 | 80487138 | G | A | 0.66 | 0.56 | 0.84 | 0.76 | 0.72 | 0.73 |
| snp27963-scaffold30-964505 | 6 | 80529707 | G | A | 0.88 | 0.53 | 0.76 | 0.78 | 0.78 | 0.64 |
| snp27964-scaffold30-997291 | 6 | 80562522 | G | A | 0.79 | 0.91 | 0.87 | 0.94 | 0.88 | 0.47 |
| snp27965-scaffold30-1052995 | 6 | 80618495 | A | G | 0.55 | 0.88 | 0.68 | 0.81 | 0.55 | 0.82 |
| snp27966-scaffold30-1100925 | 6 | 80666888 | C | A | 0.43 | 0.79 | 0.61 | 0.44 | 0.80 | 0.77 |
| snp27967-scaffold30-1155149 | 6 | 80722426 | G | A | 0.72 | 0.24 | 0.84 | 0.76 | 0.78 | 0.67 |
| snp27968-scaffold30-1186994 | 6 | 80754509 | G | A | 0.91 | 0.91 | 0.84 | 0.78 | 0.74 | 0.42 |

| | | | | | | | | | | |
|---|---|---|---|---|---|---|---|---|---|---|
| snp27970-scaffold30-1302518 | 6 | 80871189 | A | C | 0.84 | 0.91 | 0.45 | 0.74 | 0.76 | 0.30 |
| snp27971-scaffold30-1358708 | 6 | 80928192 | G | A | 0.48 | 0.32 | 0.26 | 0.33 | 0.49 | 0.68 |
| snp27972-scaffold30-1391368 | 6 | 80961227 | G | A | 1.00 | 1.00 | 1.00 | 1.00 | 0.88 | 0.96 |
| snp27973-scaffold30-1429636 | 6 | 80999485 | G | A | 0.84 | 0.79 | 0.45 | 0.65 | 0.49 | 0.34 |
| snp27974-scaffold30-1486609 | 6 | 81048778 | A | G | 0.69 | 0.62 | 0.58 | 0.80 | 0.41 | 0.70 |
| snp27975-scaffold30-1520030 | 6 | 81082870 | G | A | 0.76 | 0.91 | 0.58 | 0.63 | 0.42 | 0.82 |
| snp27976-scaffold30-1556817 | 6 | 81119618 | A | G | 0.97 | 0.88 | 0.92 | 1.00 | 0.78 | 0.63 |
| snp27977-scaffold30-1631310 | 6 | 81184205 | A | G | 0.53 | 0.79 | 0.61 | 0.63 | 0.59 | 0.76 |
| snp27978-scaffold30-1681648 | 6 | 81234324 | G | A | 0.50 | 0.26 | 0.74 | 0.37 | 0.79 | 0.54 |
| snp27979-scaffold30-1742142 | 6 | 81296263 | A | G | 0.76 | 0.50 | 0.53 | 0.69 | 0.59 | 0.90 |
| snp27980-scaffold30-1774761 | 6 | 81329069 | A | G | 0.83 | 0.82 | 0.82 | 0.74 | 0.67 | 0.62 |
| snp27981-scaffold30-1816357 | 6 | 81371444 | G | A | 0.67 | 0.59 | 0.89 | 0.74 | 0.76 | 0.47 |
| snp27983-scaffold30-1894213 | 6 | 81446938 | A | G | 0.47 | 0.62 | 0.58 | 0.70 | 0.42 | 0.42 |
| snp27984-scaffold30-1931659 | 6 | 81484447 | A | G | 0.78 | 0.88 | 1.00 | 1.00 | 0.87 | 0.73 |
| snp27985-scaffold30-1983092 | 6 | 81536933 | G | A | 0.74 | 0.91 | 0.53 | 0.94 | 0.58 | 0.31 |
| snp27986-scaffold30-2051903 | 6 | 81605977 | A | G | 0.67 | 0.68 | 0.55 | 0.83 | 0.63 | 0.86 |
| snp27987-scaffold30-2090811 | 6 | 81644997 | A | G | 0.28 | 0.50 | 0.71 | 0.37 | 0.70 | 0.29 |
| snp27989-scaffold30-2188717 | 6 | 81745070 | A | C | 0.47 | 0.79 | 0.66 | 0.19 | 0.47 | 0.51 |
| snp27990-scaffold30-2241619 | 6 | 81804590 | G | A | 0.48 | 0.62 | 0.21 | 0.67 | 0.66 | 0.52 |
| snp27991-scaffold30-2299743 | 6 | 81862750 | A | C | 0.50 | 0.53 | 0.34 | 0.67 | 0.59 | 0.66 |
| snp27993-scaffold30-2392105 | 6 | 81955680 | C | A | 0.76 | 0.76 | 0.47 | 0.48 | 0.62 | 0.41 |
| snp27994-scaffold30-2430461 | 6 | 81996225 | G | A | 0.71 | 0.44 | 0.84 | 0.98 | 0.95 | 0.82 |
| snp27995-scaffold30-2502391 | 6 | 82067011 | C | A | 0.41 | 0.76 | 0.61 | 0.57 | 0.53 | 0.67 |
| snp27996-scaffold30-2547438 | 6 | 82113901 | G | A | 0.47 | 0.71 | 0.68 | 0.54 | 0.36 | 0.76 |
| snp27997-scaffold30-2622131 | 6 | 82190587 | G | A | 0.74 | 1.00 | 0.66 | 0.48 | 0.55 | 0.69 |
| snp27998-scaffold30-2651120 | 6 | 82219509 | A | G | 0.69 | 0.97 | 0.66 | 0.85 | 0.58 | 0.89 |
| snp36190-scaffold433-12605 | 6 | 82237825 | G | A | 0.67 | 0.97 | 0.66 | 0.85 | 0.46 | 0.89 |
| snp36191-scaffold433-68322 | 6 | 82294734 | G | A | 0.64 | 0.71 | 0.53 | 0.19 | 0.55 | 0.26 |
| snp36192-scaffold433-97993 | 6 | 82324828 | G | A | 0.90 | 1.00 | 0.83 | 0.98 | 0.88 | 0.98 |
| snp36193-scaffold433-137297 | 6 | 82364116 | A | G | 0.81 | 0.65 | 0.58 | 0.76 | 0.70 | 0.61 |
| snp36196-scaffold433-277237 | 6 | 82505477 | G | A | 0.62 | 0.91 | 0.66 | 0.69 | 0.82 | 0.83 |
| snp36197-scaffold433-344971 | 6 | 82574419 | G | A | 0.41 | 0.74 | 0.83 | 0.67 | 0.59 | 0.36 |
| snp36198-scaffold433-398052 | 6 | 82628596 | G | A | 0.64 | 0.97 | 0.84 | 0.89 | 0.75 | 0.86 |
| snp36199-scaffold433-428497 | 6 | 82659501 | A | G | 0.78 | 0.85 | 0.97 | 0.78 | 0.84 | 0.50 |
| snp36200-scaffold433-467266 | 6 | 82698776 | A | G | 0.76 | 0.47 | 0.97 | 0.78 | 0.83 | 0.50 |
| snp36202-scaffold433-560512 | 6 | 82794001 | G | A | 0.74 | 0.85 | 0.82 | 0.81 | 0.79 | 0.52 |
| snp36204-scaffold433-656078 | 6 | 82889676 | A | G | 0.52 | 0.29 | 0.66 | 0.67 | 0.57 | 0.73 |
| snp36205-scaffold433-688242 | 6 | 82922328 | A | G | 0.69 | 0.74 | 0.61 | 0.52 | 0.51 | 0.40 |
| snp36206-scaffold433-748240 | 6 | 82981387 | C | A | 0.78 | 0.76 | 0.71 | 0.63 | 0.57 | 0.77 |
| snp36207-scaffold433-787868 | 6 | 83022253 | A | G | 0.72 | 0.76 | 0.63 | 0.56 | 0.47 | 0.73 |
| snp36208-scaffold433-819601 | 6 | 83054771 | A | G | 0.66 | 0.74 | 0.61 | 0.76 | 0.58 | 0.26 |
| snp36209-scaffold433-860043 | 6 | 83096191 | G | A | 0.84 | 0.97 | 0.76 | 0.72 | 0.53 | 0.90 |
| snp36210-scaffold433-891877 | 6 | 83127701 | C | A | 0.48 | 0.18 | 0.42 | 0.67 | 0.43 | 0.70 |

| | | | | | | | | | | |
|---|---|---|---|---|---|---|---|---|---|---|
| snp36211-scaffold433-971139 | 6 | 83207760 | A | G | 0.69 | 0.44 | 0.47 | 0.48 | 0.88 | 0.11 |
| snp36212-scaffold433-1024144 | 6 | 83262034 | A | G | 0.43 | 0.74 | 0.37 | 0.50 | 0.63 | 0.61 |
| snp36213-scaffold433-1053757 | 6 | 83291790 | A | G | 0.66 | 0.44 | 0.66 | 0.65 | 0.86 | 0.33 |
| snp36214-scaffold433-1103329 | 6 | 83341158 | G | A | 0.50 | 0.44 | 0.42 | 0.59 | 0.59 | 0.66 |
| snp36215-scaffold433-1162426 | 6 | 83400683 | C | A | 0.90 | 0.53 | 0.79 | 0.78 | 0.78 | 0.66 |
| snp36216-scaffold433-1192931 | 6 | 83432886 | A | G | 0.95 | 0.56 | 0.61 | 0.78 | 0.88 | 0.64 |
| snp36217-scaffold433-1250993 | 6 | 83490914 | A | G | 0.88 | 1.00 | 0.97 | 0.93 | 0.96 | 0.99 |
| snp36218-scaffold433-1280540 | 6 | 83520224 | A | G | 0.60 | 0.24 | 0.61 | 0.59 | 0.78 | 0.52 |
| snp36219-scaffold433-1310547 | 6 | 83550656 | C | A | 0.67 | 0.74 | 0.53 | 0.59 | 0.62 | 0.44 |
| snp36220-scaffold433-1340607 | 6 | 83580837 | C | A | 0.78 | 0.88 | 0.66 | 0.69 | 0.86 | 0.59 |
| snp36221-scaffold433-1374323 | 6 | 83615137 | A | C | 0.84 | 0.97 | 0.61 | 0.91 | 0.58 | 0.88 |
| snp36222-scaffold433-1408607 | 6 | 83649674 | A | G | 0.48 | 0.59 | 0.37 | 0.56 | 0.09 | 0.82 |
| snp36223-scaffold433-1442030 | 6 | 83683404 | A | G | 0.47 | 0.53 | 0.66 | 0.80 | 0.43 | 0.54 |
| snp36224-scaffold433-1472222 | 6 | 83714029 | G | A | 0.66 | 0.91 | 0.71 | 0.96 | 0.75 | 0.56 |
| snp36225-scaffold433-1504984 | 6 | 83747591 | G | A | 0.50 | 0.71 | 0.79 | 0.59 | 0.53 | 0.79 |
| snp36226-scaffold433-1563136 | 6 | 83805443 | A | G | 0.86 | 0.91 | 0.89 | 0.94 | 0.88 | 0.61 |
| snp36227-scaffold433-1593977 | 6 | 83836718 | A | G | 0.47 | 0.15 | 0.58 | 0.63 | 0.51 | 0.39 |
| snp36228-scaffold433-1673264 | 6 | 83917146 | G | A | 0.95 | 0.88 | 0.87 | 0.98 | 0.62 | 0.91 |
| snp36229-scaffold433-1724202 | 6 | 83969244 | A | G | 0.90 | 0.74 | 0.87 | 0.72 | 0.88 | 0.59 |
| snp36231-scaffold433-1813258 | 6 | 84058441 | G | A | 0.84 | 0.41 | 0.42 | 0.61 | 0.59 | 0.42 |
| snp36232-scaffold433-1851751 | 6 | 84097782 | G | A | 0.88 | 0.41 | 0.61 | 0.61 | 0.66 | 0.50 |
| snp36233-scaffold433-1880537 | 6 | 84126889 | C | A | 0.86 | 0.82 | 0.61 | 0.61 | 0.64 | 0.46 |
| snp36234-scaffold433-1944012 | 6 | 84191204 | A | G | 0.88 | 0.41 | 0.92 | 0.74 | 0.61 | 0.60 |
| snp36235-scaffold433-1980454 | 6 | 84227636 | A | G | 0.64 | 0.59 | 0.74 | 0.83 | 0.78 | 0.84 |
| snp36236-scaffold433-2014363 | 6 | 84261953 | A | C | 0.83 | 0.82 | 0.82 | 0.72 | 0.76 | 0.96 |
| snp36239-scaffold433-2114932 | 6 | 84363630 | G | A | 0.72 | 0.50 | 0.82 | 0.80 | 0.71 | 0.90 |
| snp36240-scaffold433-2150890 | 6 | 84399875 | G | A | 0.60 | 0.88 | 0.87 | 0.78 | 0.70 | 0.28 |
| snp36242-scaffold433-2243946 | 6 | 84494693 | A | G | 0.48 | 0.12 | 0.45 | 0.81 | 0.59 | 0.80 |
| snp36243-scaffold433-2290839 | 6 | 84550394 | G | A | 0.88 | 0.94 | 0.95 | 0.87 | 0.91 | 0.60 |
| snp36244-scaffold433-2327090 | 6 | 84587037 | A | C | 0.66 | 1.00 | 0.58 | 0.80 | 0.78 | 0.91 |
| snp36245-scaffold433-2369729 | 6 | 84630269 | G | A | 0.74 | 0.41 | 0.68 | 0.57 | 0.68 | 0.59 |
| snp36246-scaffold433-2410081 | 6 | 84670863 | A | G | 0.43 | 0.74 | 0.66 | 0.63 | 0.49 | 0.54 |
| snp36247-scaffold433-2458008 | 6 | 84718290 | A | G | 0.91 | 0.74 | 0.74 | 0.44 | 0.87 | 0.67 |
| snp36248-scaffold433-2496408 | 6 | 84756877 | A | G | 0.83 | 0.76 | 0.82 | 0.83 | 0.71 | 0.33 |
| snp36249-scaffold433-2541429 | 6 | 84803192 | A | G | 0.98 | 0.91 | 0.95 | 0.91 | 0.95 | 0.72 |
| snp36250-scaffold433-2598226 | 6 | 84859242 | A | G | 0.69 | 0.74 | 0.53 | 0.76 | 0.54 | 0.47 |
| snp36251-scaffold433-2643408 | 6 | 84905194 | A | C | 0.41 | 0.85 | 0.45 | 0.20 | 0.55 | 0.30 |
| snp59410-scaffold980-39769 | 6 | 85723625 | G | A | 0.71 | 0.97 | 0.74 | 0.59 | 0.64 | 0.53 |
| snp59413-scaffold980-176278 | 6 | 85861188 | G | A | 0.90 | 0.44 | 0.82 | 0.87 | 0.76 | 0.38 |
| snp59414-scaffold980-228963 | 6 | 85913488 | G | A | 0.81 | 0.44 | 0.84 | 0.78 | 0.80 | 0.56 |
| snp59416-scaffold980-293987 | 6 | 85978469 | A | G | 0.88 | 0.24 | 0.58 | 0.59 | 0.41 | 0.91 |
| snp59417-scaffold980-295173 | 6 | 85979655 | A | G | 0.88 | 0.85 | 0.76 | 0.61 | 0.68 | 0.22 |
| snp59426-scaffold980-307288 | 6 | 85991683 | A | C | 0.24 | 0.88 | 0.66 | 0.93 | 0.59 | 0.24 |

| | | | | | | | | | | |
|---|---|---|---|---|---|---|---|---|---|---|
| snp59427-scaffold980-309132 | 6 | 85994156 | G | A | 0.24 | 0.88 | 0.66 | 0.93 | 0.59 | 0.24 |
| snp59428-scaffold980-310410 | 6 | 85995436 | G | A | 0.24 | 0.88 | 0.66 | 0.93 | 0.59 | 0.24 |
| snp59429-scaffold980-310523 | 6 | 85995549 | A | G | 0.24 | 0.88 | 0.66 | 0.93 | 0.59 | 0.24 |
| snp59430-scaffold980-311053 | 6 | 85996079 | A | G | 0.24 | 0.88 | 0.66 | 0.93 | 0.59 | 0.24 |
| snp59431-scaffold980-311510 | 6 | 85996534 | A | G | 0.95 | 1.00 | 0.79 | 1.00 | 0.70 | 0.62 |
| snp59432-scaffold980-311547 | 6 | 85996571 | A | G | 0.26 | 0.88 | 0.66 | 0.93 | 0.59 | 0.24 |
| snp59434-scaffold980-322977 | 6 | 86007956 | A | G | 0.71 | 0.12 | 0.32 | 0.07 | 0.41 | 0.79 |
| snp59435-scaffold980-323008 | 6 | 86007987 | G | A | 1.00 | 1.00 | 1.00 | 1.00 | 1.00 | 1.00 |
| snp59438-scaffold980-323773 | 6 | 86008752 | G | A | 0.71 | 0.12 | 0.32 | 0.07 | 0.41 | 0.79 |
| snp59439-scaffold980-324311 | 6 | 86009290 | A | G | 0.71 | 0.12 | 0.32 | 0.07 | 0.41 | 0.79 |
| snp59441-scaffold980-325353 | 6 | 86010332 | A | G | 0.71 | 0.12 | 0.32 | 0.07 | 0.41 | 0.79 |
| snp59442-scaffold980-364892 | 6 | 86050088 | A | G | 0.72 | 0.44 | 0.53 | 0.78 | 0.54 | 0.44 |
| snp59444-scaffold980-395682 | 6 | 86080909 | C | A | 0.50 | 0.94 | 0.82 | 0.80 | 0.96 | 0.94 |
| snp59445-scaffold980-395848 | 6 | 86081075 | C | A | 0.71 | 0.41 | 0.68 | 0.80 | 0.62 | 0.62 |
| snp59447-scaffold980-398647 | 6 | 86083874 | C | A | 0.38 | 0.44 | 0.61 | 0.81 | 0.88 | 0.63 |
| snp59448-scaffold980-399351 | 6 | 86084578 | G | A | 1.00 | 1.00 | 0.97 | 1.00 | 0.95 | 0.91 |
| snp59450-scaffold980-400667 | 6 | 86085897 | A | G | 0.86 | 0.65 | 0.61 | 0.24 | 0.46 | 0.52 |
| snp59451-scaffold980-402598 | 6 | 86087828 | G | A | 0.90 | 0.65 | 0.61 | 0.43 | 0.47 | 0.57 |
| snp59454-scaffold980-403305 | 6 | 86088536 | A | G | 0.90 | 0.65 | 0.61 | 0.43 | 0.47 | 0.57 |
| snp59455-scaffold980-407817 | 6 | 86093124 | G | A | 0.86 | 0.65 | 0.61 | 0.43 | 0.47 | 0.57 |
| snp59458-scaffold980-434555 | 6 | 86118732 | C | A | 0.84 | 0.65 | 0.63 | 0.44 | 0.47 | 0.57 |
| snp59459-scaffold980-469488 | 6 | 86155374 | A | G | 0.90 | 0.62 | 0.58 | 0.44 | 0.47 | 0.57 |
| snp59460-scaffold980-512583 | 6 | 86198470 | G | A | 0.33 | 0.74 | 0.53 | 0.78 | 0.75 | 0.63 |
| snp59463-scaffold980-517776 | 6 | 86203664 | A | G | 0.22 | 0.65 | 0.53 | 0.74 | 0.75 | 0.67 |
| snp59465-scaffold980-522477 | 6 | 86208823 | G | A | 1.00 | 0.88 | 0.95 | 1.00 | 0.99 | 0.99 |
| snp59466-scaffold980-522552 | 6 | 86208898 | A | G | 0.98 | 1.00 | 0.95 | 0.93 | 0.89 | 1.00 |
| snp59467-scaffold980-522554 | 6 | 86208900 | A | G | 1.00 | 1.00 | 1.00 | 0.94 | 1.00 | 1.00 |
| snp59468-scaffold980-522581 | 6 | 86208927 | A | G | 1.00 | 1.00 | 1.00 | 1.00 | 1.00 | 1.00 |
| snp59469-scaffold980-522616 | 6 | 86208962 | G | A | 0.98 | 1.00 | 0.95 | 0.93 | 0.89 | 1.00 |
| snp59471-scaffold980-522691 | 6 | 86209037 | G | A | 1.00 | 1.00 | 1.00 | 1.00 | 1.00 | 1.00 |
| snp59472-scaffold980-522778 | 6 | 86209124 | A | G | 0.93 | 0.94 | 0.95 | 0.78 | 0.79 | 0.57 |
| snp59473-scaffold980-523359 | 6 | 86209708 | A | G | 0.86 | 0.41 | 0.58 | 0.48 | 0.47 | 0.79 |
| snp59476-scaffold980-611628 | 6 | 86296898 | A | G | 0.83 | 0.91 | 0.74 | 0.80 | 0.67 | 0.88 |
| snp59477-scaffold980-650487 | 6 | 86335395 | G | A | 0.43 | 0.88 | 0.61 | 0.52 | 0.59 | 0.51 |
| snp59478-scaffold980-719185 | 6 | 86403884 | A | C | 0.69 | 0.53 | 0.50 | 0.65 | 0.45 | 0.60 |
| snp59479-scaffold980-756733 | 6 | 86441636 | A | G | 0.55 | 0.32 | 0.66 | 0.89 | 0.41 | 0.58 |
| snp59480-scaffold980-805769 | 6 | 86490735 | G | A | 0.78 | 0.94 | 0.76 | 0.81 | 0.76 | 0.44 |
| snp59481-scaffold980-845143 | 6 | 86530389 | G | A | 0.81 | 0.71 | 0.74 | 0.52 | 0.67 | 0.81 |
| snp59482-scaffold980-897515 | 6 | 86582982 | A | G | 0.60 | 0.26 | 0.71 | 0.44 | 0.62 | 0.58 |
| snp59483-scaffold980-932164 | 6 | 86617643 | A | G | 0.53 | 0.85 | 0.63 | 0.67 | 0.55 | 0.86 |
| snp59485-scaffold980-1037986 | 6 | 86723526 | A | C | 0.88 | 0.71 | 0.87 | 0.85 | 0.99 | 0.89 |
| snp59486-scaffold980-1068168 | 6 | 86753823 | G | A | 0.71 | 1.00 | 0.97 | 0.72 | 0.75 | 0.67 |
| snp59488-scaffold980-1135359 | 6 | 86822367 | A | G | 0.53 | 0.24 | 0.53 | 0.33 | 0.63 | 0.41 |

| | | | | | | | | | | |
|---|---|---|---|---|---|---|---|---|---|---|
| snp59489-scaffold980-1170267 | 6 | 86858026 | A | G | 0.66 | 0.97 | 0.79 | 0.81 | 0.63 | 0.77 |
| snp59490-scaffold980-1217770 | 6 | 86905655 | G | A | 0.46 | 0.85 | 0.76 | 0.87 | 0.63 | 0.68 |
| snp59491-scaffold980-1264886 | 6 | 86946839 | A | G | 0.86 | 1.00 | 0.84 | 0.89 | 0.87 | 0.56 |
| snp59492-scaffold980-1308491 | 6 | 86990418 | G | A | 0.62 | 0.68 | 0.66 | 0.59 | 0.61 | 0.32 |
| snp59493-scaffold980-1344268 | 6 | 87026431 | G | A | 0.83 | 0.79 | 0.82 | 1.00 | 0.88 | 0.50 |
| snp59494-scaffold980-1377724 | 6 | 87059305 | G | A | 0.93 | 0.79 | 0.53 | 0.26 | 0.80 | 0.73 |
| snp59495-scaffold980-1409219 | 6 | 87091375 | A | G | 0.48 | 0.44 | 0.53 | 0.44 | 0.78 | 0.37 |
| snp59498-scaffold980-1542962 | 6 | 87224552 | A | G | 0.91 | 0.82 | 0.74 | 0.67 | 0.67 | 0.87 |
| snp59499-scaffold980-1597986 | 6 | 87277318 | A | G | 0.41 | 0.24 | 0.61 | 0.76 | 0.49 | 0.39 |
| snp59500-scaffold980-1653956 | 6 | 87333420 | G | A | 0.83 | 0.88 | 0.50 | 0.35 | 0.51 | 0.63 |
| snp59501-scaffold980-1696492 | 6 | 87375279 | A | G | 0.48 | 0.68 | 0.79 | 0.59 | 0.66 | 0.47 |
| snp59502-scaffold980-1754315 | 6 | 87432568 | A | G | 0.78 | 0.71 | 0.45 | 0.87 | 0.61 | 0.43 |
| snp59503-scaffold980-1809268 | 6 | 87486180 | G | A | 0.48 | 0.09 | 0.45 | 0.52 | 0.74 | 0.40 |
| snp59504-scaffold980-1844220 | 6 | 87520913 | G | A | 0.69 | 0.71 | 0.47 | 0.81 | 0.59 | 0.64 |
| snp59505-scaffold980-1885171 | 6 | 87562136 | G | A | 0.86 | 0.97 | 0.92 | 0.87 | 0.86 | 0.63 |
| snp59506-scaffold980-1929596 | 6 | 87607376 | A | C | 0.24 | 0.68 | 0.79 | 0.69 | 0.45 | 0.83 |
| snp59507-scaffold980-1963541 | 6 | 87642965 | A | G | 0.72 | 0.53 | 0.53 | 0.52 | 0.36 | 0.61 |
| snp59508-scaffold980-1996928 | 6 | 87676461 | A | G | 0.79 | 0.76 | 0.82 | 0.78 | 0.74 | 0.90 |
| snp59509-scaffold980-2038725 | 6 | 87717680 | A | G | 0.84 | 0.76 | 0.66 | 0.61 | 0.67 | 0.82 |
| snp59510-scaffold980-2081926 | 6 | 87763495 | A | G | 0.64 | 0.91 | 0.89 | 0.81 | 0.79 | 0.94 |
| snp59511-scaffold980-2118157 | 6 | 87799781 | A | G | 0.79 | 0.82 | 0.74 | 0.74 | 0.57 | 0.52 |
| snp59512-scaffold980-2175698 | 6 | 87856632 | A | C | 0.60 | 0.79 | 0.42 | 0.54 | 0.39 | 0.62 |
| snp59514-scaffold980-2238150 | 6 | 87918820 | G | A | 0.83 | 0.82 | 0.87 | 0.93 | 0.91 | 0.57 |
| snp59881-scaffold996-25451 | 6 | 87947666 | A | G | 0.83 | 0.68 | 0.92 | 0.85 | 0.74 | 0.90 |
| snp59882-scaffold996-56860 | 6 | 87977437 | G | A | 0.67 | 0.71 | 0.66 | 0.48 | 0.45 | 0.68 |
| snp59883-scaffold996-96450 | 6 | 88017011 | G | A | 0.59 | 0.71 | 0.89 | 0.89 | 0.78 | 0.73 |
| snp59884-scaffold996-137936 | 6 | 88058887 | A | G | 0.69 | 0.65 | 0.74 | 0.56 | 0.54 | 0.21 |
| snp59885-scaffold996-174893 | 6 | 88098751 | A | G | 0.53 | 0.41 | 0.47 | 0.44 | 0.64 | 0.44 |
| snp59886-scaffold996-206707 | 6 | 88129137 | A | C | 0.48 | 0.82 | 0.63 | 0.96 | 0.47 | 0.31 |
| snp59887-scaffold996-240805 | 6 | 88163548 | G | A | 0.48 | 0.41 | 0.53 | 0.06 | 0.59 | 0.67 |
| snp59888-scaffold996-270759 | 6 | 88193909 | G | A | 0.97 | 0.85 | 0.87 | 1.00 | 0.76 | 0.90 |
| snp59889-scaffold996-319198 | 6 | 88242608 | G | A | 0.76 | 0.71 | 0.66 | 0.39 | 0.39 | 0.30 |
| snp59890-scaffold996-373156 | 6 | 88296849 | C | A | 0.55 | 0.62 | 0.47 | 0.52 | 0.53 | 0.48 |
| snp59892-scaffold996-439249 | 6 | 88364177 | A | G | 0.52 | 0.65 | 0.53 | 0.76 | 0.63 | 0.69 |
| snp7871-scaffold1281-3251602 | 6 | 88369601 | A | C | 0.69 | 0.44 | 0.68 | 0.57 | 0.80 | 0.53 |
| snp7870-scaffold1281-3209251 | 6 | 88413999 | G | A | 0.79 | 0.62 | 0.61 | 0.59 | 0.75 | 0.59 |
| snp7869-scaffold1281-3146516 | 6 | 88477919 | A | G | 0.62 | 0.94 | 0.89 | 0.78 | 0.78 | 0.82 |
| snp7867-scaffold1281-3069811 | 6 | 88548044 | A | G | 0.91 | 0.94 | 0.97 | 0.89 | 0.76 | 0.91 |
| snp7865-scaffold1281-2993308 | 6 | 88625379 | G | A | 0.84 | 0.62 | 0.92 | 0.81 | 0.79 | 0.86 |
| snp7864-scaffold1281-2946675 | 6 | 88672088 | A | G | 0.95 | 1.00 | 0.97 | 0.89 | 0.83 | 0.82 |
| snp7863-scaffold1281-2905738 | 6 | 88713081 | A | G | 0.69 | 0.88 | 0.63 | 0.81 | 0.68 | 0.52 |
| snp7862-scaffold1281-2867994 | 6 | 88751476 | G | A | 0.67 | 0.76 | 0.61 | 0.80 | 0.62 | 0.37 |
| snp7859-scaffold1281-2744288 | 6 | 88875483 | G | A | 0.69 | 0.82 | 0.61 | 0.89 | 0.71 | 0.24 |

| | | | | | | | | | | |
|---|---|---|---|---|---|---|---|---|---|---|
| snp7858-scaffold1281-2709785 | 6 | 88910244 | A | G | 0.60 | 0.74 | 0.50 | 0.87 | 0.66 | 0.22 |
| snp7857-scaffold1281-2669753 | 6 | 88950303 | A | C | 0.41 | 0.79 | 0.66 | 0.70 | 0.80 | 0.82 |
| snp7856-scaffold1281-2633459 | 6 | 88987240 | G | A | 0.84 | 1.00 | 0.71 | 0.70 | 0.86 | 0.96 |
| snp7854-scaffold1281-2573893 | 6 | 89046486 | G | A | 0.64 | 0.56 | 0.45 | 0.78 | 0.62 | 0.30 |
| snp7853-scaffold1281-2539045 | 6 | 89081427 | G | A | 0.74 | 0.26 | 0.55 | 0.28 | 0.63 | 0.82 |
| snp7851-scaffold1281-2453837 | 6 | 89164178 | A | G | 0.93 | 0.88 | 1.00 | 0.89 | 0.91 | 0.92 |
| snp7850-scaffold1281-2389999 | 6 | 89228405 | G | A | 0.80 | 0.71 | 0.55 | 0.85 | 0.66 | 0.84 |
| snp7849-scaffold1281-2347312 | 6 | 89271447 | G | A | 0.83 | 0.82 | 0.79 | 0.96 | 0.45 | 0.82 |
| snp7848-scaffold1281-2308363 | 6 | 89311000 | G | A | 0.74 | 0.76 | 0.74 | 0.83 | 0.68 | 0.84 |
| snp7846-scaffold1281-2224915 | 6 | 89392374 | G | A | 0.76 | 0.76 | 0.34 | 0.65 | 0.53 | 0.60 |
| snp7845-scaffold1281-2182425 | 6 | 89433782 | A | G | 0.83 | 0.79 | 0.61 | 1.00 | 0.58 | 0.86 |
| snp7844-scaffold1281-2147026 | 6 | 89469205 | A | G | 0.76 | 0.74 | 0.39 | 0.87 | 0.76 | 0.67 |
| snp7843-scaffold1281-2095611 | 6 | 89521067 | G | A | 0.72 | 0.68 | 0.58 | 0.76 | 0.68 | 0.29 |
| snp7842-scaffold1281-2032198 | 6 | 89585687 | A | G | 0.72 | 0.59 | 0.74 | 0.96 | 0.62 | 0.37 |
| snp7841-scaffold1281-1981418 | 6 | 89636526 | A | G | 0.62 | 0.79 | 0.50 | 0.83 | 0.71 | 0.59 |
| snp7840-scaffold1281-1944793 | 6 | 89673335 | A | G | 0.53 | 0.68 | 0.47 | 0.72 | 0.71 | 0.51 |
| snp7839-scaffold1281-1909013 | 6 | 89709498 | C | A | 0.55 | 0.79 | 0.76 | 0.78 | 0.61 | 0.77 |
| snp7838-scaffold1281-1858089 | 6 | 89760275 | G | A | 0.57 | 0.65 | 0.61 | 0.59 | 0.61 | 0.61 |
| snp7835-scaffold1281-1746790 | 6 | 89871220 | G | A | 0.67 | 0.50 | 0.79 | 0.85 | 0.54 | 0.27 |
| snp7834-scaffold1281-1711923 | 6 | 89906464 | G | A | 0.78 | 0.62 | 0.68 | 0.74 | 0.51 | 0.73 |
| snp7833-scaffold1281-1682312 | 6 | 89936218 | A | G | 0.76 | 0.76 | 0.84 | 0.65 | 0.76 | 0.58 |
| snp7832-scaffold1281-1651749 | 6 | 89966767 | G | A | 0.66 | 0.50 | 0.71 | 0.57 | 0.66 | 0.96 |
| snp7831-scaffold1281-1622621 | 6 | 89996685 | A | G | 0.66 | 0.56 | 0.87 | 0.69 | 0.72 | 0.68 |
| snp7829-scaffold1281-1546878 | 6 | 90073626 | G | A | 0.33 | 0.79 | 0.63 | 0.65 | 0.54 | 0.58 |
| snp7828-scaffold1281-1487138 | 6 | 90133650 | A | G | 0.74 | 0.56 | 0.84 | 0.89 | 0.67 | 0.47 |
| snp7827-scaffold1281-1452740 | 6 | 90168010 | G | A | 0.78 | 0.82 | 0.79 | 0.81 | 0.80 | 0.94 |
| snp7826-scaffold1281-1419043 | 6 | 90201761 | A | C | 0.52 | 0.74 | 0.45 | 0.54 | 0.61 | 0.60 |
| snp7825-scaffold1281-1387041 | 6 | 90233727 | G | A | 0.36 | 0.62 | 0.66 | 0.72 | 0.46 | 0.41 |
| snp24359-scaffold247-7445771 | 11 | 44937429 | A | G | 0.83 | 0.62 | 0.18 | 0.70 | 0.38 | 0.64 |
| snp24362-scaffold247-7612447 | 11 | 45106300 | G | A | 0.50 | 0.74 | 0.50 | 0.50 | 0.41 | 0.63 |
| snp24363-scaffold247-7671529 | 11 | 45165248 | G | A | 0.81 | 0.88 | 0.97 | 0.98 | 0.80 | 0.94 |
| snp24364-scaffold247-7700241 | 11 | 45193954 | G | A | 0.71 | 0.38 | 0.53 | 0.65 | 0.61 | 0.83 |
| snp24365-scaffold247-7737011 | 11 | 45230573 | G | A | 0.74 | 0.47 | 0.68 | 0.76 | 0.55 | 0.68 |
| snp24366-scaffold247-7788661 | 11 | 45283938 | A | G | 0.71 | 0.59 | 0.84 | 0.61 | 0.21 | 0.59 |
| snp24367-scaffold247-7817474 | 11 | 45312792 | A | C | 0.93 | 0.71 | 0.95 | 0.72 | 0.45 | 0.64 |
| snp24368-scaffold247-7849544 | 11 | 45344976 | G | A | 0.46 | 0.62 | 0.74 | 0.50 | 0.86 | 0.44 |
| snp24369-scaffold247-7885248 | 11 | 45380355 | G | A | 0.43 | 0.62 | 0.66 | 0.57 | 0.83 | 0.58 |
| snp24370-scaffold247-7918087 | 11 | 45413563 | C | A | 0.38 | 0.41 | 0.53 | 0.59 | 0.87 | 0.68 |
| snp24372-scaffold247-7985455 | 11 | 45481199 | G | A | 0.47 | 0.65 | 0.29 | 0.61 | 0.51 | 0.46 |
| snp24373-scaffold247-8014995 | 11 | 45510693 | G | A | 0.67 | 0.65 | 0.34 | 0.76 | 0.72 | 0.43 |
| snp24375-scaffold247-8126221 | 11 | 45620617 | A | G | 0.79 | 0.09 | 0.66 | 0.43 | 0.58 | 0.50 |
| snp24376-scaffold247-8163187 | 11 | 45658154 | G | A | 0.45 | 0.76 | 0.89 | 0.63 | 0.70 | 0.41 |
| snp24377-scaffold247-8195661 | 11 | 45690410 | G | A | 0.74 | 0.56 | 0.92 | 0.74 | 0.50 | 0.69 |

| | | | | | | | | | | |
|---|---|---|---|---|---|---|---|---|---|---|
| snp24378-scaffold247-8237399 | 11 | 45733434 | C | A | 0.38 | 0.56 | 0.84 | 0.57 | 0.36 | 0.37 |
| snp24379-scaffold247-8271176 | 11 | 45767199 | G | A | 0.86 | 0.88 | 0.95 | 0.98 | 0.70 | 0.91 |
| snp24380-scaffold247-8312050 | 11 | 45807874 | G | A | 0.71 | 0.85 | 0.39 | 0.96 | 0.64 | 0.89 |
| snp24381-scaffold247-8348343 | 11 | 45834714 | A | G | 0.50 | 0.62 | 0.34 | 0.87 | 0.61 | 0.76 |
| snp24382-scaffold247-8397562 | 11 | 45883991 | G | A | 0.78 | 0.85 | 0.71 | 0.94 | 0.63 | 0.83 |
| snp24383-scaffold247-8432697 | 11 | 45919329 | C | A | 0.53 | 0.88 | 0.89 | 0.67 | 0.70 | 0.76 |
| snp24384-scaffold247-8476501 | 11 | 45963596 | G | A | 0.66 | 0.47 | 0.71 | 0.72 | 0.64 | 0.70 |
| snp24385-scaffold247-8511046 | 11 | 45998519 | A | G | 0.41 | 0.56 | 0.34 | 0.52 | 0.88 | 0.47 |
| snp24386-scaffold247-8549140 | 11 | 46037044 | A | G | 0.72 | 0.91 | 0.66 | 0.35 | 0.76 | 0.53 |
| snp24388-scaffold247-8639566 | 11 | 46126917 | A | G | 0.84 | 0.79 | 0.97 | 0.65 | 0.74 | 0.88 |
| snp24390-scaffold247-8701572 | 11 | 46188430 | C | A | 0.74 | 0.85 | 0.92 | 0.83 | 0.71 | 0.53 |
| snp24391-scaffold247-8735760 | 11 | 46222740 | A | G | 0.84 | 0.53 | 0.68 | 0.62 | 0.87 | 0.73 |
| snp24393-scaffold247-8807369 | 11 | 46293120 | A | G | 0.69 | 0.53 | 0.82 | 0.41 | 0.39 | 0.63 |
| snp24394-scaffold247-8844300 | 11 | 46330321 | G | A | 0.74 | 0.41 | 0.61 | 0.26 | 0.55 | 0.52 |
| snp24395-scaffold247-8900755 | 11 | 46387211 | C | A | 0.86 | 0.65 | 0.89 | 0.78 | 0.96 | 0.70 |
| snp24396-scaffold247-8934763 | 11 | 46421230 | A | G | 0.55 | 0.44 | 0.68 | 0.46 | 0.37 | 0.78 |
| snp24397-scaffold247-8994694 | 11 | 46482074 | A | G | 0.62 | 0.29 | 0.66 | 0.69 | 0.54 | 0.57 |
| snp24398-scaffold247-9043653 | 11 | 46531087 | A | G | 0.81 | 0.74 | 0.45 | 0.76 | 0.50 | 0.88 |
| snp24399-scaffold247-9097794 | 11 | 46585437 | G | A | 0.59 | 0.74 | 0.89 | 0.89 | 0.46 | 0.57 |
| snp24400-scaffold247-9134050 | 11 | 46621817 | C | A | 0.45 | 0.50 | 0.58 | 0.76 | 0.55 | 0.66 |
| snp24401-scaffold247-9172226 | 11 | 46659855 | A | G | 0.57 | 0.62 | 0.68 | 0.69 | 0.36 | 0.51 |
| snp24402-scaffold247-9229849 | 11 | 46718038 | A | G | 0.57 | 0.68 | 0.82 | 0.54 | 0.72 | 0.40 |
| snp24403-scaffold247-9268934 | 11 | 46757106 | G | A | 0.66 | 0.41 | 0.89 | 0.54 | 0.57 | 0.50 |
| snp24404-scaffold247-9300158 | 11 | 46788463 | C | A | 0.67 | 0.26 | 0.76 | 0.80 | 0.67 | 0.58 |
| snp52890-scaffold793-1539305 | 11 | 46855479 | A | G | 0.78 | 0.56 | 0.53 | 0.39 | 0.89 | 0.46 |
| snp52887-scaffold793-1411479 | 11 | 46983525 | G | A | 0.29 | 0.59 | 0.37 | 0.61 | 0.55 | 0.71 |
| snp52886-scaffold793-1359234 | 11 | 47036182 | C | A | 0.79 | 0.32 | 0.68 | 0.63 | 0.63 | 0.64 |
| snp52885-scaffold793-1311040 | 11 | 47084441 | A | G | 0.33 | 0.56 | 0.74 | 0.63 | 0.71 | 0.68 |
| snp52884-scaffold793-1281501 | 11 | 47113940 | C | A | 0.38 | 0.38 | 0.56 | 0.65 | 0.57 | 0.52 |
| snp52883-scaffold793-1222376 | 11 | 47173627 | G | A | 0.81 | 0.85 | 0.61 | 0.65 | 0.78 | 0.76 |
| snp52882-scaffold793-1164646 | 11 | 47231511 | A | G | 0.84 | 1.00 | 0.89 | 0.81 | 0.62 | 0.86 |
| snp52881-scaffold793-1094831 | 11 | 47301569 | G | A | 0.38 | 0.82 | 0.11 | 0.56 | 0.75 | 0.34 |
| snp52880-scaffold793-1060593 | 11 | 47335810 | A | G | 0.67 | 0.59 | 0.29 | 0.26 | 0.63 | 0.37 |
| snp52877-scaffold793-931038 | 11 | 47465558 | G | A | 0.81 | 0.82 | 0.84 | 0.96 | 0.80 | 1.00 |
| snp52875-scaffold793-852206 | 11 | 47544671 | A | G | 0.67 | 0.71 | 0.11 | 0.56 | 0.49 | 0.36 |
| snp52874-scaffold793-813615 | 11 | 47583238 | A | G | 0.78 | 0.53 | 0.68 | 0.48 | 0.58 | 0.67 |
| snp52873-scaffold793-768148 | 11 | 47624079 | G | A | 0.53 | 0.53 | 0.24 | 0.24 | 0.55 | 0.63 |
| snp52872-scaffold793-735741 | 11 | 47657460 | A | G | 0.52 | 0.53 | 0.71 | 0.57 | 0.79 | 0.73 |
| snp52870-scaffold793-645929 | 11 | 47747561 | A | G | 0.78 | 0.65 | 0.92 | 0.81 | 0.45 | 0.93 |
| snp52869-scaffold793-615567 | 11 | 47778061 | G | A | 0.59 | 0.68 | 0.61 | 0.56 | 0.84 | 0.44 |
| snp52868-scaffold793-580969 | 11 | 47812727 | A | G | 0.71 | 0.91 | 0.63 | 0.81 | 0.67 | 0.79 |
| snp52867-scaffold793-538503 | 11 | 47854992 | A | G | 0.83 | 0.71 | 0.18 | 0.48 | 0.45 | 0.54 |
| snp52866-scaffold793-484637 | 11 | 47909803 | G | A | 0.90 | 0.88 | 1.00 | 1.00 | 0.72 | 0.98 |

| | | | | | | | | | | |
|---|---|---|---|---|---|---|---|---|---|---|
| snp52865-scaffold793-422288 | 11 | 47972151 | A | G | 0.62 | 0.62 | 0.92 | 0.57 | 0.75 | 0.80 |
| snp52864-scaffold793-390417 | 11 | 48004035 | G | A | 0.69 | 0.94 | 0.63 | 0.93 | 0.83 | 0.81 |
| snp52862-scaffold793-266577 | 11 | 48128154 | G | A | 0.88 | 0.44 | 0.63 | 0.61 | 0.93 | 0.53 |
| snp52861-scaffold793-226779 | 11 | 48168440 | A | G | 0.79 | 0.68 | 0.61 | 0.74 | 0.84 | 0.86 |
| snp52860-scaffold793-172964 | 11 | 48222099 | G | A | 0.66 | 0.71 | 0.42 | 0.87 | 0.58 | 0.62 |
| snp52859-scaffold793-142628 | 11 | 48252222 | G | A | 0.64 | 0.74 | 0.68 | 0.61 | 0.46 | 0.17 |
| snp52858-scaffold793-109796 | 11 | 48285062 | A | G | 0.81 | 0.74 | 0.97 | 0.85 | 0.74 | 0.86 |
| snp52857-scaffold793-77949 | 11 | 48316904 | A | G | 0.52 | 0.68 | 0.63 | 0.78 | 0.57 | 0.63 |
| snp52856-scaffold793-42405 | 11 | 48351916 | G | A | 0.60 | 0.44 | 0.74 | 0.78 | 0.62 | 0.61 |
| snp52855-scaffold793-6368 | 11 | 48387716 | G | A | 0.71 | 0.79 | 0.76 | 0.63 | 0.59 | 0.66 |
| snp3581-scaffold1111-40020 | 11 | 48433549 | A | G | 0.83 | 0.79 | 0.97 | 0.91 | 0.93 | 0.73 |
| snp3582-scaffold1111-88000 | 11 | 48479319 | A | G | 0.64 | 0.41 | 0.37 | 0.56 | 0.42 | 0.59 |
| snp3584-scaffold1111-191133 | 11 | 48582501 | G | A | 0.83 | 0.44 | 0.18 | 0.24 | 0.55 | 0.73 |
| snp3585-scaffold1111-242003 | 11 | 48634130 | G | A | 0.66 | 0.29 | 0.92 | 0.28 | 0.72 | 0.81 |
| snp3586-scaffold1111-276061 | 11 | 48667927 | G | A | 0.43 | 0.47 | 0.84 | 0.41 | 0.46 | 0.79 |
| snp3587-scaffold1111-306302 | 11 | 48697880 | G | A | 0.71 | 0.74 | 0.97 | 0.78 | 0.87 | 0.72 |
| snp3588-scaffold1111-352606 | 11 | 48744005 | A | C | 0.71 | 0.76 | 0.87 | 0.69 | 0.63 | 0.59 |
| snp3589-scaffold1111-383039 | 11 | 48774326 | G | A | 0.43 | 0.38 | 0.42 | 0.63 | 0.76 | 0.59 |
| snp3590-scaffold1111-418306 | 11 | 48809542 | A | G | 0.57 | 0.82 | 0.63 | 0.81 | 0.75 | 0.80 |
| snp3591-scaffold1111-454455 | 11 | 48845878 | G | A | 0.57 | 0.56 | 0.74 | 0.94 | 0.78 | 0.90 |
| snp3592-scaffold1111-496847 | 11 | 48888368 | C | A | 0.59 | 0.53 | 0.61 | 0.85 | 0.88 | 0.89 |
| snp3593-scaffold1111-542260 | 11 | 48933899 | C | A | 0.72 | 0.84 | 0.71 | 0.85 | 0.46 | 0.82 |
| snp3594-scaffold1111-599861 | 11 | 48991424 | A | G | 0.83 | 0.59 | 0.61 | 0.96 | 0.92 | 0.93 |
| snp3595-scaffold1111-644798 | 11 | 49036557 | G | A | 0.93 | 0.44 | 0.89 | 0.94 | 0.93 | 0.93 |
| snp3596-scaffold1111-739858 | 11 | 49131546 | A | G | 0.83 | 0.85 | 0.76 | 0.50 | 0.80 | 0.52 |
| snp3597-scaffold1111-792154 | 11 | 49183449 | G | A | 0.47 | 0.76 | 0.79 | 0.31 | 0.88 | 0.60 |
| snp3598-scaffold1111-824439 | 11 | 49216140 | C | A | 0.50 | 0.50 | 0.50 | 0.65 | 0.67 | 0.49 |
| snp3599-scaffold1111-857855 | 11 | 49249374 | G | A | 0.69 | 0.56 | 0.68 | 0.63 | 0.53 | 0.44 |
| snp3601-scaffold1111-923713 | 11 | 49314966 | A | G | 0.67 | 0.88 | 0.87 | 0.80 | 0.70 | 0.81 |
| snp13020-scaffold150-1792026 | 13 | 48951107 | G | A | 0.55 | 0.47 | 0.32 | 0.85 | 0.59 | 0.60 |
| snp13021-scaffold150-1826857 | 13 | 48986366 | G | A | 0.67 | 0.62 | 0.63 | 0.93 | 0.86 | 0.58 |
| snp13022-scaffold150-1858408 | 13 | 49018452 | G | A | 0.69 | 0.59 | 0.76 | 0.96 | 0.59 | 0.89 |
| snp13023-scaffold150-1887308 | 13 | 49047497 | C | A | 0.60 | 0.32 | 0.58 | 0.85 | 0.96 | 0.60 |
| snp13024-scaffold150-1957010 | 13 | 49118295 | C | A | 0.47 | 0.26 | 0.50 | 0.57 | 0.43 | 0.60 |
| snp13026-scaffold150-2046045 | 13 | 49202152 | A | G | 0.55 | 0.71 | 0.53 | 0.67 | 0.57 | 0.80 |
| snp13027-scaffold150-2077281 | 13 | 49233293 | A | G | 0.76 | 0.79 | 0.87 | 0.54 | 0.78 | 0.84 |
| snp13028-scaffold150-2135081 | 13 | 49293957 | G | A | 0.59 | 0.71 | 1.00 | 0.81 | 0.57 | 0.67 |
| snp13030-scaffold150-2216985 | 13 | 49376927 | A | G | 1.00 | 0.88 | 1.00 | 1.00 | 0.92 | 0.93 |
| snp13031-scaffold150-2247207 | 13 | 49406518 | A | G | 0.79 | 0.97 | 1.00 | 0.89 | 0.59 | 0.87 |
| snp13032-scaffold150-2345286 | 13 | 49505570 | G | A | 0.55 | 0.71 | 0.42 | 0.56 | 0.57 | 0.53 |
| snp13033-scaffold150-2378517 | 13 | 49539282 | C | A | 0.57 | 0.44 | 0.84 | 0.52 | 0.84 | 0.61 |
| snp13034-scaffold150-2419465 | 13 | 49580468 | G | A | 0.76 | 0.44 | 0.68 | 0.43 | 0.78 | 0.70 |
| snp13035-scaffold150-2488901 | 13 | 49652002 | A | G | 0.41 | 0.41 | 0.82 | 0.20 | 0.80 | 0.48 |

| | | | | | | | | | | |
|---|---|---|---|---|---|---|---|---|---|---|
| snp13036-scaffold150-2537469 | 13 | 49701618 | G | A | 0.90 | 0.47 | 0.92 | 0.65 | 0.79 | 0.77 |
| snp13038-scaffold150-2617014 | 13 | 49781549 | A | G | 0.64 | 0.56 | 0.87 | 0.78 | 0.72 | 0.82 |
| snp13039-scaffold150-2652402 | 13 | 49817428 | A | C | 0.86 | 0.91 | 0.89 | 0.85 | 0.88 | 0.87 |
| snp13040-scaffold150-2688016 | 13 | 49846097 | G | A | 0.41 | 0.74 | 0.74 | 0.80 | 0.70 | 0.54 |
| snp13041-scaffold150-2723930 | 13 | 49882015 | A | G | 0.48 | 0.35 | 0.38 | 0.52 | 0.53 | 0.36 |
| snp13042-scaffold150-2772062 | 13 | 49930534 | A | G | 0.72 | 0.65 | 0.32 | 0.59 | 0.51 | 0.73 |
| snp13047-scaffold150-2998877 | 13 | 49975695 | G | A | 0.57 | 0.56 | 0.58 | 0.46 | 0.54 | 0.78 |
| snp13048-scaffold150-3055872 | 13 | 50031156 | A | G | 0.69 | 0.74 | 0.79 | 0.70 | 0.47 | 0.72 |
| snp13049-scaffold150-3085519 | 13 | 50061032 | G | A | 0.71 | 0.26 | 0.79 | 0.69 | 0.63 | 0.69 |
| snp13051-scaffold150-3186404 | 13 | 50164018 | G | A | 0.60 | 0.82 | 0.47 | 0.69 | 0.83 | 0.64 |
| snp13052-scaffold150-3240111 | 13 | 50218479 | G | A | 0.91 | 0.97 | 0.95 | 0.98 | 0.93 | 0.81 |
| snp13053-scaffold150-3268945 | 13 | 50247575 | A | G | 0.59 | 0.62 | 0.71 | 0.09 | 0.79 | 0.67 |
| snp13055-scaffold150-3341797 | 13 | 50319963 | G | A | 0.60 | 0.50 | 0.66 | 0.61 | 0.82 | 0.56 |
| snp13056-scaffold150-3390278 | 13 | 50368850 | G | A | 0.72 | 0.47 | 0.84 | 0.70 | 0.21 | 0.49 |
| snp13057-scaffold150-3423510 | 13 | 50402269 | A | C | 0.72 | 0.91 | 0.45 | 0.78 | 0.91 | 0.84 |
| snp13058-scaffold150-3456786 | 13 | 50435691 | A | G | 0.45 | 0.62 | 0.34 | 0.85 | 0.34 | 0.57 |
| snp13060-scaffold150-3557141 | 13 | 50537367 | A | G | 0.84 | 0.97 | 0.87 | 0.83 | 0.21 | 0.76 |
| snp13061-scaffold150-3602836 | 13 | 50583481 | G | A | 0.79 | 0.82 | 0.32 | 0.78 | 0.22 | 0.73 |
| snp13062-scaffold150-3671333 | 13 | 50652626 | G | A | 0.74 | 0.65 | 0.92 | 0.78 | 0.92 | 0.67 |
| snp13063-scaffold150-3710652 | 13 | 50692019 | A | C | 0.53 | 0.50 | 0.66 | 0.78 | 0.88 | 0.61 |
| snp13064-scaffold150-3771102 | 13 | 50752869 | A | G | 0.88 | 0.76 | 0.39 | 0.85 | 0.22 | 0.69 |
| snp13065-scaffold150-3854094 | 13 | 50835654 | A | G | 0.69 | 0.97 | 0.58 | 0.74 | 0.42 | 0.66 |
| snp13066-scaffold150-3913775 | 13 | 50895013 | G | A | 0.31 | 0.53 | 0.89 | 0.41 | 0.32 | 0.66 |
| snp13068-scaffold150-4002526 | 13 | 50984377 | G | A | 0.38 | 0.47 | 0.24 | 0.57 | 0.75 | 0.66 |
| snp13069-scaffold150-4040893 | 13 | 51022622 | A | G | 0.78 | 0.47 | 0.29 | 0.80 | 0.80 | 0.64 |
| snp13070-scaffold150-4109148 | 13 | 51090982 | A | G | 0.69 | 0.62 | 0.29 | 0.54 | 0.79 | 0.59 |
| snp13071-scaffold150-4161862 | 13 | 51144910 | A | G | 0.71 | 0.88 | 0.42 | 0.85 | 0.86 | 0.60 |
| snp13072-scaffold150-4198124 | 13 | 51181225 | C | A | 0.55 | 0.47 | 0.76 | 0.85 | 0.86 | 0.76 |
| snp13073-scaffold150-4265303 | 13 | 51248444 | A | G | 0.55 | 0.50 | 0.34 | 0.72 | 0.68 | 0.69 |
| snp13074-scaffold150-4295197 | 13 | 51278339 | G | A | 0.90 | 0.97 | 0.95 | 0.96 | 0.72 | 0.77 |
| snp13077-scaffold150-4411928 | 13 | 51394969 | G | A | 0.64 | 0.38 | 0.18 | 0.37 | 0.72 | 0.59 |
| snp13079-scaffold150-4516025 | 13 | 51495702 | G | A | 0.88 | 0.71 | 0.34 | 0.63 | 0.92 | 0.63 |
| snp13080-scaffold150-4562367 | 13 | 51541972 | G | A | 0.81 | 0.44 | 0.71 | 0.70 | 0.61 | 0.47 |
| snp13083-scaffold150-4670453 | 13 | 51650456 | A | G | 0.88 | 0.97 | 0.47 | 0.78 | 0.87 | 0.67 |
| snp13084-scaffold150-4752164 | 13 | 51732085 | A | G | 0.95 | 1.00 | 0.92 | 0.63 | 0.58 | 0.83 |
| snp13086-scaffold150-4840688 | 13 | 51821206 | A | G | 0.36 | 0.79 | 0.24 | 0.56 | 0.87 | 0.48 |
| snp13087-scaffold150-4881772 | 13 | 51862548 | A | G | 0.55 | 0.47 | 0.76 | 0.69 | 1.00 | 0.60 |
| snp13090-scaffold150-4982136 | 13 | 51962712 | A | G | 0.67 | 0.74 | 0.76 | 0.69 | 0.66 | 0.70 |
| snp13091-scaffold150-5011275 | 13 | 51992005 | G | A | 0.62 | 0.41 | 0.24 | 0.85 | 0.61 | 0.71 |
| snp13092-scaffold150-5061100 | 13 | 52042210 | A | G | 0.47 | 0.74 | 0.66 | 0.50 | 0.86 | 0.72 |
| snp13093-scaffold150-5103276 | 13 | 52084278 | G | A | 0.43 | 0.62 | 0.79 | 0.81 | 0.45 | 0.77 |
| snp13094-scaffold150-5171386 | 13 | 52152350 | G | A | 0.36 | 0.71 | 0.45 | 0.91 | 0.49 | 0.80 |
| snp13095-scaffold150-5234067 | 13 | 52215406 | G | A | 0.81 | 1.00 | 0.47 | 0.76 | 0.92 | 0.87 |

| | | | | | | | | | | |
|---|---|---|---|---|---|---|---|---|---|---|
| snp13096-scaffold150-5283349 | 13 | 52265157 | A | C | 0.79 | 0.76 | 0.68 | 0.70 | 0.88 | 0.53 |
| snp13097-scaffold150-5318958 | 13 | 52300613 | G | A | 0.97 | 0.82 | 1.00 | 0.89 | 1.00 | 0.86 |
| snp13098-scaffold150-5377130 | 13 | 52358539 | G | A | 0.36 | 0.74 | 0.84 | 1.00 | 0.84 | 0.72 |
| snp13099-scaffold150-5409187 | 13 | 52390960 | G | A | 0.79 | 0.97 | 0.92 | 1.00 | 0.88 | 0.60 |
| snp13100-scaffold150-5437865 | 13 | 52420154 | G | A | 0.71 | 0.47 | 0.87 | 0.46 | 0.74 | 0.86 |
| snp13101-scaffold150-5532904 | 13 | 52515170 | G | A | 0.90 | 0.97 | 1.00 | 0.81 | 0.62 | 0.73 |
| snp13102-scaffold150-5576981 | 13 | 52559153 | G | A | 0.88 | 0.97 | 0.50 | 0.57 | 0.68 | 0.74 |
| snp13103-scaffold150-5652311 | 13 | 52634493 | A | G | 0.74 | 0.97 | 0.84 | 0.94 | 0.99 | 0.84 |
| snp13104-scaffold150-5704786 | 13 | 52686956 | G | A | 0.67 | 0.50 | 0.50 | 0.74 | 0.47 | 0.53 |
| snp13106-scaffold150-5816268 | 13 | 52799493 | A | G | 0.34 | 0.53 | 0.50 | 0.72 | 0.51 | 0.82 |
| snp57653-scaffold924-31639 | 13 | 52871150 | A | G | 0.76 | 0.94 | 0.74 | 1.00 | 0.88 | 0.94 |
| snp57656-scaffold924-127281 | 13 | 52967054 | G | A | 0.62 | 0.15 | 0.29 | 0.67 | 0.83 | 0.41 |
| snp57657-scaffold924-156245 | 13 | 52996046 | A | G | 0.41 | 0.53 | 0.58 | 0.80 | 0.41 | 0.63 |
| snp1640-scaffold10469-208327 | 13 | 53160922 | G | A | 0.78 | 0.74 | 0.42 | 0.54 | 0.55 | 0.30 |
| snp1639-scaffold10469-54125 | 13 | 53305564 | A | G | 0.53 | 0.62 | 0.63 | 0.85 | 0.50 | 0.86 |
| snp3082-scaffold10942-56333 | 13 | 53415534 | A | G | 0.45 | 0.79 | 0.39 | 0.65 | 0.91 | 0.68 |
| snp3083-scaffold10942-139774 | 13 | 53499132 | A | G | 0.52 | 0.56 | 0.66 | 0.65 | 0.93 | 0.68 |
| snp3084-scaffold10942-184058 | 13 | 53543335 | A | G | 0.52 | 0.94 | 0.53 | 0.52 | 0.89 | 0.60 |
| snp3086-scaffold10942-256040 | 13 | 53615633 | G | A | 0.45 | 0.53 | 0.63 | 0.48 | 0.91 | 0.56 |
| snp47987-scaffold677-32710 | 13 | 53941826 | C | A | 0.53 | 0.68 | 0.53 | 0.74 | 0.70 | 0.36 |
| snp47988-scaffold677-71484 | 13 | 53980581 | G | A | 0.74 | 0.29 | 0.47 | 0.22 | 0.11 | 0.74 |
| snp47989-scaffold677-111621 | 13 | 54020763 | A | G | 0.66 | 0.91 | 0.63 | 0.83 | 0.58 | 0.47 |
| snp47991-scaffold677-242551 | 13 | 54148578 | A | G | 0.48 | 0.79 | 0.55 | 0.43 | 0.51 | 0.37 |
| snp47992-scaffold677-303503 | 13 | 54234723 | A | C | 0.52 | 0.29 | 0.42 | 0.46 | 0.87 | 0.82 |
| snp47993-scaffold677-382501 | 13 | 54283307 | A | G | 0.76 | 0.26 | 0.37 | 0.48 | 0.84 | 0.87 |
| snp59143-scaffold97-1334327 | 13 | 54343916 | A | G | 0.43 | 0.79 | 0.55 | 0.57 | 0.55 | 0.59 |
| snp59142-scaffold97-1302272 | 13 | 54375905 | A | G | 0.50 | 0.62 | 0.76 | 0.48 | 0.36 | 0.46 |
| snp59140-scaffold97-1234161 | 13 | 54443908 | G | A | 0.69 | 0.82 | 0.87 | 0.83 | 0.39 | 0.46 |
| snp59139-scaffold97-1161128 | 13 | 54517150 | A | G | 0.69 | 0.35 | 0.63 | 0.72 | 0.83 | 0.78 |
| snp59138-scaffold97-1130980 | 13 | 54547712 | G | A | 0.60 | 0.44 | 0.84 | 0.70 | 0.80 | 0.73 |
| snp59137-scaffold97-1070618 | 13 | 54608080 | A | G | 0.69 | 0.62 | 0.76 | 0.72 | 0.84 | 0.73 |
| snp59136-scaffold97-1039608 | 13 | 54639178 | G | A | 0.69 | 0.56 | 0.82 | 0.63 | 0.96 | 0.67 |
| snp59134-scaffold97-945914 | 13 | 54732654 | A | G | 0.93 | 0.76 | 0.68 | 1.00 | 0.63 | 0.78 |
| snp59133-scaffold97-914274 | 13 | 54764299 | A | G | 0.78 | 0.91 | 0.63 | 0.57 | 0.62 | 0.77 |
| snp59132-scaffold97-881836 | 13 | 54796713 | A | G | 0.57 | 0.56 | 0.55 | 0.41 | 0.42 | 0.40 |
| snp59130-scaffold97-798683 | 13 | 54879725 | G | A | 0.53 | 0.53 | 0.66 | 0.48 | 0.53 | 0.57 |
| snp59129-scaffold97-750673 | 13 | 54927643 | A | G | 0.69 | 0.91 | 1.00 | 0.93 | 0.57 | 0.92 |
| snp59126-scaffold97-602516 | 13 | 55074522 | A | G | 0.93 | 0.79 | 0.89 | 0.91 | 0.62 | 0.84 |
| snp59125-scaffold97-555694 | 13 | 55121476 | A | G | 0.33 | 0.38 | 0.74 | 0.70 | 0.67 | 0.74 |
| snp59124-scaffold97-514510 | 13 | 55162546 | A | G | 0.36 | 0.62 | 0.42 | 0.81 | 0.61 | 0.58 |
| snp59123-scaffold97-481812 | 13 | 55195097 | G | A | 0.90 | 0.85 | 0.76 | 0.56 | 0.76 | 0.77 |
| snp59122-scaffold97-446740 | 13 | 55230170 | A | G | 0.71 | 0.74 | 0.42 | 0.44 | 0.70 | 0.34 |
| snp59121-scaffold97-396456 | 13 | 55279634 | A | G | 0.78 | 0.88 | 0.74 | 0.70 | 0.55 | 0.83 |

| | | | | | | | | | | |
|---|---|---|---|---|---|---|---|---|---|---|
| snp59120-scaffold97-338545 | 13 | 55337155 | A | G | 0.50 | 0.59 | 0.61 | 0.61 | 0.18 | 0.53 |
| snp59119-scaffold97-300805 | 13 | 55373792 | A | G | 0.64 | 0.53 | 0.55 | 0.56 | 0.46 | 0.88 |
| snp59118-scaffold97-249935 | 13 | 55424701 | A | G | 0.57 | 0.71 | 0.71 | 0.89 | 0.62 | 0.60 |
| snp59116-scaffold97-145693 | 13 | 55529385 | G | A | 0.88 | 1.00 | 1.00 | 1.00 | 0.93 | 0.80 |
| snp59115-scaffold97-112867 | 13 | 55562284 | A | G | 0.71 | 0.97 | 0.82 | 0.81 | 0.67 | 0.70 |
| snp59114-scaffold97-79343 | 13 | 55595704 | G | A | 0.48 | 0.47 | 0.34 | 0.78 | 0.55 | 0.39 |
| snp59113-scaffold97-16178 | 13 | 55659262 | A | G | 0.48 | 0.76 | 0.89 | 0.39 | 0.87 | 0.96 |
| snp51639-scaffold758-2053604 | 13 | 55726046 | G | A | 0.74 | 0.44 | 0.76 | 0.72 | 0.70 | 0.62 |
| snp51637-scaffold758-1972385 | 13 | 55807622 | A | G | 0.59 | 0.71 | 0.63 | 0.58 | 0.20 | 0.91 |
| snp51636-scaffold758-1938854 | 13 | 55839931 | G | A | 0.84 | 0.32 | 0.53 | 0.83 | 0.45 | 0.62 |
| snp51635-scaffold758-1904594 | 13 | 55874198 | A | C | 0.74 | 0.79 | 0.95 | 0.89 | 0.82 | 0.67 |
| snp51633-scaffold758-1784016 | 13 | 55995273 | C | A | 0.64 | 0.29 | 0.50 | 0.76 | 0.76 | 0.79 |
| snp51631-scaffold758-1680193 | 13 | 56099350 | G | A | 0.64 | 0.53 | 0.74 | 0.72 | 0.63 | 0.28 |
| snp51630-scaffold758-1648572 | 13 | 56130867 | A | G | 0.43 | 0.38 | 0.71 | 0.69 | 0.62 | 0.70 |
| snp51629-scaffold758-1596469 | 13 | 56182537 | G | A | 0.83 | 1.00 | 0.71 | 0.96 | 0.54 | 0.77 |
| snp51628-scaffold758-1538532 | 13 | 56240654 | A | G | 0.74 | 0.82 | 0.74 | 0.37 | 0.70 | 0.46 |
| snp51627-scaffold758-1501836 | 13 | 56277331 | A | G | 0.79 | 0.26 | 0.79 | 0.59 | 0.43 | 0.67 |
| snp51626-scaffold758-1464555 | 13 | 56314647 | A | G | 0.69 | 0.79 | 0.74 | 0.63 | 0.74 | 0.84 |
| snp51625-scaffold758-1433001 | 13 | 56346258 | G | A | 0.79 | 0.62 | 0.84 | 0.59 | 0.54 | 0.44 |
| snp51624-scaffold758-1349045 | 13 | 56429888 | G | A | 0.93 | 0.97 | 0.84 | 0.67 | 0.97 | 0.94 |
| snp51623-scaffold758-1317085 | 13 | 56462200 | A | C | 0.83 | 0.85 | 0.84 | 0.46 | 0.97 | 0.94 |
| snp51621-scaffold758-1246638 | 13 | 56532819 | G | A | 0.98 | 1.00 | 0.97 | 0.93 | 0.99 | 0.92 |
| snp51620-scaffold758-1185419 | 13 | 56593905 | A | G | 0.59 | 0.32 | 0.86 | 0.57 | 0.45 | 0.32 |
| snp51619-scaffold758-1137940 | 13 | 56641339 | A | G | 0.81 | 1.00 | 0.89 | 0.89 | 0.91 | 0.94 |
| snp51618-scaffold758-1076685 | 13 | 56703165 | G | A | 0.60 | 0.97 | 0.92 | 0.94 | 0.61 | 0.67 |
| snp51617-scaffold758-1046091 | 13 | 56734036 | G | A | 0.74 | 0.38 | 0.89 | 0.70 | 0.83 | 0.43 |
| snp51616-scaffold758-987448 | 13 | 56792225 | A | G | 0.81 | 0.94 | 0.92 | 0.94 | 0.96 | 0.87 |
| snp51615-scaffold758-951832 | 13 | 56827986 | A | G | 0.83 | 0.74 | 0.87 | 0.78 | 0.75 | 0.68 |
| snp51614-scaffold758-921766 | 13 | 56857987 | A | G | 0.69 | 0.59 | 0.95 | 0.72 | 0.74 | 0.64 |
| snp51611-scaffold758-759914 | 13 | 57019624 | G | A | 0.71 | 0.38 | 0.84 | 0.48 | 0.70 | 0.58 |
| snp51609-scaffold758-682359 | 13 | 57097091 | G | A | 0.81 | 0.76 | 0.26 | 0.83 | 0.86 | 0.74 |
| snp51608-scaffold758-623630 | 13 | 57155515 | C | A | 0.33 | 0.38 | 0.45 | 0.72 | 0.61 | 0.80 |
| snp51607-scaffold758-576864 | 13 | 57201918 | G | A | 0.47 | 0.56 | 0.39 | 0.39 | 0.76 | 0.39 |
| snp51606-scaffold758-537192 | 13 | 57241444 | G | A | 0.28 | 0.47 | 0.68 | 0.43 | 0.34 | 0.57 |
| snp51605-scaffold758-507235 | 13 | 57271315 | G | A | 0.90 | 0.85 | 0.76 | 0.56 | 0.83 | 0.39 |
| snp51604-scaffold758-468102 | 13 | 57310819 | G | A | 0.43 | 0.35 | 0.66 | 0.54 | 0.61 | 0.60 |
| snp51603-scaffold758-435287 | 13 | 57344078 | A | G | 0.90 | 0.85 | 1.00 | 0.96 | 0.99 | 0.56 |
| snp51602-scaffold758-401439 | 13 | 57378109 | C | A | 0.55 | 0.85 | 0.26 | 0.67 | 0.71 | 0.77 |
| snp51601-scaffold758-348533 | 13 | 57430935 | G | A | 0.97 | 0.97 | 0.63 | 0.96 | 0.91 | 0.96 |
| snp51599-scaffold758-231150 | 13 | 57548364 | A | G | 0.64 | 0.85 | 0.42 | 0.81 | 0.58 | 0.48 |
| snp51598-scaffold758-200850 | 13 | 57578599 | A | G | 0.69 | 0.59 | 0.76 | 0.57 | 0.78 | 0.62 |
| snp51597-scaffold758-148867 | 13 | 57630450 | A | G | 0.53 | 0.59 | 0.63 | 0.59 | 0.39 | 0.62 |
| snp51595-scaffold758-81029 | 13 | 57697180 | G | A | 0.36 | 0.76 | 0.18 | 0.52 | 0.86 | 0.38 |

| | | | | | | | | | | |
|---|---|---|---|---|---|---|---|---|---|---|
| snp51594-scaffold758-41618 | 13 | 57736476 | G | A | 0.53 | 0.32 | 0.82 | 0.57 | 0.45 | 0.68 |
| snp51593-scaffold758-4807 | 13 | 57773651 | G | A | 0.47 | 0.29 | 0.68 | 0.87 | 0.54 | 0.30 |
| snp38035-scaffold4735-20143 | 13 | 57799650 | A | G | 0.65 | 0.82 | 0.76 | 0.83 | 0.66 | 0.76 |
| snp48732-scaffold691-4056627 | 13 | 57835346 | G | A | 0.86 | 0.88 | 0.97 | 0.81 | 0.97 | 0.94 |
| snp48731-scaffold691-4007331 | 13 | 57885043 | A | C | 0.55 | 0.85 | 0.50 | 0.57 | 0.76 | 0.52 |
| snp48730-scaffold691-3968776 | 13 | 57923892 | A | G | 0.66 | 0.79 | 0.71 | 0.43 | 0.62 | 0.33 |
| snp48728-scaffold691-3891907 | 13 | 58000655 | G | A | 0.88 | 0.59 | 0.76 | 0.78 | 0.64 | 0.52 |
| snp48727-scaffold691-3844983 | 13 | 58047381 | C | A | 0.66 | 0.74 | 0.53 | 0.89 | 0.47 | 0.86 |
| snp48726-scaffold691-3812400 | 13 | 58079901 | G | A | 0.97 | 0.91 | 0.76 | 0.78 | 0.59 | 0.56 |
| snp48725-scaffold691-3771222 | 13 | 58121171 | G | A | 0.60 | 0.35 | 0.82 | 0.41 | 0.57 | 0.52 |
| snp48724-scaffold691-3724638 | 13 | 58167847 | G | A | 0.81 | 0.94 | 0.97 | 0.96 | 0.71 | 0.97 |
| snp48723-scaffold691-3676681 | 13 | 58215991 | G | A | 0.60 | 0.47 | 0.29 | 0.33 | 0.68 | 0.64 |
| snp48722-scaffold691-3642650 | 13 | 58250129 | G | A | 0.64 | 0.82 | 0.29 | 0.72 | 0.88 | 0.74 |
| snp48721-scaffold691-3606611 | 13 | 58286133 | G | A | 0.57 | 0.53 | 0.42 | 0.37 | 0.84 | 0.77 |
| snp48720-scaffold691-3577941 | 13 | 58315126 | A | G | 0.90 | 0.50 | 0.97 | 0.80 | 0.89 | 0.81 |
| snp48719-scaffold691-3522528 | 13 | 58370939 | A | C | 0.41 | 0.76 | 0.74 | 0.37 | 0.57 | 0.73 |
| snp48718-scaffold691-3491671 | 13 | 58401733 | G | A | 0.64 | 0.91 | 0.92 | 0.33 | 0.84 | 0.76 |
| snp48717-scaffold691-3449579 | 13 | 58443484 | A | G | 0.53 | 0.32 | 0.37 | 0.78 | 0.71 | 0.80 |
| snp48716-scaffold691-3419004 | 13 | 58473970 | A | G | 0.74 | 0.82 | 0.79 | 0.69 | 0.84 | 0.81 |
| snp48715-scaffold691-3389021 | 13 | 58504312 | G | A | 0.50 | 0.15 | 0.26 | 0.35 | 0.68 | 0.84 |
| snp48714-scaffold691-3339003 | 13 | 58554268 | G | A | 0.57 | 0.79 | 0.79 | 0.69 | 0.37 | 0.39 |
| snp48713-scaffold691-3275295 | 13 | 58617614 | C | A | 0.98 | 0.88 | 0.92 | 1.00 | 0.89 | 1.00 |
| snp48712-scaffold691-3242076 | 13 | 58650956 | G | A | 0.76 | 0.53 | 0.87 | 0.48 | 0.76 | 0.78 |
| snp48711-scaffold691-3212981 | 13 | 58677046 | G | A | 0.76 | 0.62 | 0.87 | 0.93 | 0.46 | 0.43 |
| snp48710-scaffold691-3182114 | 13 | 58708491 | C | A | 0.71 | 0.68 | 0.42 | 0.50 | 0.61 | 0.73 |
| snp48709-scaffold691-3151884 | 13 | 58737467 | G | A | 0.66 | 0.79 | 0.82 | 0.65 | 0.54 | 0.54 |
| snp48708-scaffold691-3121607 | 13 | 58768300 | A | G | 0.53 | 0.71 | 0.68 | 0.87 | 0.78 | 0.76 |
| snp48705-scaffold691-2985461 | 13 | 58903680 | A | G | 0.95 | 0.88 | 1.00 | 0.98 | 0.91 | 0.94 |
| snp48704-scaffold691-2931706 | 13 | 58957839 | A | G | 0.41 | 0.56 | 0.82 | 0.31 | 0.34 | 0.49 |
| snp48703-scaffold691-2873716 | 13 | 59016648 | A | G | 0.52 | 0.53 | 0.89 | 0.46 | 0.59 | 0.63 |
| snp48701-scaffold691-2797883 | 13 | 59092877 | G | A | 0.76 | 0.94 | 0.87 | 0.91 | 0.83 | 0.73 |
| snp48699-scaffold691-2724642 | 13 | 59166204 | A | G | 0.60 | 0.38 | 0.71 | 0.44 | 0.57 | 0.42 |
| snp48697-scaffold691-2652764 | 13 | 59238655 | A | G | 0.70 | 0.32 | 0.42 | 0.67 | 0.64 | 0.72 |
| snp48696-scaffold691-2617271 | 13 | 59273709 | C | A | 0.59 | 0.76 | 0.76 | 0.43 | 0.51 | 0.52 |
| snp48695-scaffold691-2576130 | 13 | 59314956 | A | G | 0.72 | 0.85 | 0.76 | 0.44 | 0.70 | 0.69 |
| snp48694-scaffold691-2541731 | 13 | 59347221 | A | C | 0.67 | 0.65 | 0.24 | 0.81 | 0.45 | 0.53 |
| snp48693-scaffold691-2512532 | 13 | 59377448 | G | A | 0.88 | 0.85 | 0.53 | 0.83 | 0.59 | 0.76 |
| snp48692-scaffold691-2441767 | 13 | 59447744 | A | G | 0.69 | 0.59 | 0.74 | 0.80 | 0.47 | 0.60 |
| snp48690-scaffold691-2355185 | 13 | 59533966 | A | G | 0.71 | 0.35 | 0.47 | 0.26 | 0.83 | 0.69 |
| snp48689-scaffold691-2271551 | 13 | 59617972 | A | G | 0.98 | 0.85 | 1.00 | 0.85 | 0.91 | 1.00 |
| snp48687-scaffold691-2208058 | 13 | 59691809 | G | A | 0.76 | 0.41 | 0.16 | 0.69 | 0.88 | 0.67 |
| snp48686-scaffold691-2175979 | 13 | 59723770 | A | C | 0.93 | 1.00 | 0.97 | 0.94 | 0.55 | 0.91 |
| snp48685-scaffold691-2121763 | 13 | 59777889 | A | G | 0.78 | 0.47 | 0.21 | 0.69 | 0.67 | 0.66 |

| | | | | | | | | | | |
|---|---|---|---|---|---|---|---|---|---|---|
| snp48684-scaffold691-2078262 | 13 | 59821510 | A | G | 0.86 | 0.74 | 0.97 | 0.57 | 0.88 | 0.76 |
| snp48683-scaffold691-2018665 | 13 | 59881287 | A | G | 0.93 | 0.91 | 0.45 | 0.69 | 0.84 | 0.54 |
| snp48682-scaffold691-1951001 | 13 | 59949924 | G | A | 0.38 | 0.38 | 0.16 | 0.46 | 0.63 | 0.48 |
| snp48680-scaffold691-1814336 | 13 | 60086997 | A | C | 0.26 | 0.32 | 0.42 | 0.76 | 0.25 | 0.79 |
| snp48676-scaffold691-1659135 | 13 | 60244049 | A | G | 0.60 | 0.53 | 0.37 | 0.48 | 0.59 | 0.60 |
| snp48675-scaffold691-1611256 | 13 | 60292162 | G | A | 0.66 | 0.82 | 0.95 | 0.76 | 0.86 | 0.51 |
| snp48673-scaffold691-1501695 | 13 | 60401596 | G | A | 0.60 | 0.50 | 0.42 | 0.65 | 0.64 | 0.40 |
| snp48672-scaffold691-1468331 | 13 | 60434676 | A | G | 0.81 | 0.94 | 0.97 | 1.00 | 0.88 | 0.94 |
| snp48671-scaffold691-1413629 | 13 | 60489154 | C | A | 0.64 | 0.62 | 0.92 | 0.41 | 0.58 | 0.41 |
| snp48670-scaffold691-1370255 | 13 | 60532573 | G | A | 0.53 | 0.50 | 0.42 | 0.78 | 0.43 | 0.72 |
| snp48669-scaffold691-1330986 | 13 | 60571904 | A | G | 0.53 | 0.76 | 0.76 | 0.30 | 0.71 | 0.64 |
| snp48668-scaffold691-1301732 | 13 | 60601288 | G | A | 0.48 | 0.56 | 0.68 | 0.59 | 0.80 | 0.50 |
| snp48667-scaffold691-1265329 | 13 | 60637928 | G | A | 0.69 | 0.56 | 0.50 | 0.78 | 0.42 | 0.61 |
| snp48666-scaffold691-1231504 | 13 | 60671725 | G | A | 0.88 | 0.74 | 0.47 | 0.31 | 0.47 | 0.66 |
| snp48664-scaffold691-1156130 | 13 | 60746881 | A | G | 0.61 | 0.71 | 0.47 | 0.70 | 0.74 | 0.60 |
| snp48663-scaffold691-1120968 | 13 | 60781203 | A | G | 0.38 | 0.26 | 0.58 | 0.63 | 0.61 | 0.38 |
| snp48662-scaffold691-1087196 | 13 | 60815005 | A | G | 0.50 | 0.68 | 0.71 | 0.46 | 0.32 | 0.46 |
| snp48660-scaffold691-985507 | 13 | 60915069 | A | G | 0.57 | 0.79 | 0.74 | 0.46 | 0.71 | 0.40 |
| snp48659-scaffold691-944258 | 13 | 60956345 | A | G | 0.86 | 0.74 | 0.89 | 0.78 | 0.45 | 0.76 |
| snp48658-scaffold691-871881 | 13 | 61027609 | A | G | 0.55 | 0.53 | 0.53 | 0.35 | 0.75 | 0.64 |
| snp48657-scaffold691-826662 | 13 | 61072258 | A | G | 0.69 | 0.65 | 0.66 | 0.81 | 0.33 | 0.39 |
| snp48655-scaffold691-737821 | 13 | 61183998 | G | A | 0.59 | 0.85 | 0.87 | 0.80 | 0.37 | 0.58 |
| snp48654-scaffold691-694962 | 13 | 61226592 | A | G | 0.57 | 0.32 | 0.26 | 0.72 | 0.71 | 0.67 |
| snp48653-scaffold691-665303 | 13 | 61255797 | G | A | 0.93 | 0.82 | 0.97 | 0.89 | 0.86 | 0.83 |
| snp48652-scaffold691-636088 | 13 | 61284707 | A | G | 0.83 | 0.71 | 0.92 | 0.44 | 0.92 | 0.64 |
| snp48651-scaffold691-601620 | 13 | 61318983 | G | A | 0.52 | 0.41 | 0.63 | 0.69 | 0.50 | 0.36 |
| snp48650-scaffold691-560168 | 13 | 61360898 | G | A | 0.88 | 0.82 | 0.95 | 0.50 | 0.87 | 0.96 |
| snp48649-scaffold691-515657 | 13 | 61405104 | G | A | 0.66 | 0.82 | 0.82 | 0.39 | 0.82 | 0.74 |
| snp48648-scaffold691-438587 | 13 | 61482403 | G | A | 0.36 | 0.71 | 0.63 | 0.26 | 0.84 | 0.63 |
| snp48647-scaffold691-382618 | 13 | 61538470 | G | A | 0.69 | 0.53 | 0.42 | 0.78 | 0.38 | 0.66 |
| snp48646-scaffold691-352628 | 13 | 61568515 | A | G | 0.86 | 0.88 | 1.00 | 0.91 | 0.91 | 0.72 |
| snp48644-scaffold691-245299 | 13 | 61663216 | A | G | 0.81 | 0.79 | 0.74 | 0.35 | 0.83 | 0.89 |
| snp48642-scaffold691-163083 | 13 | 61744929 | A | G | 0.60 | 0.71 | 1.00 | 0.78 | 0.96 | 0.66 |
| snp48641-scaffold691-99462 | 13 | 61809352 | G | A | 0.57 | 0.68 | 0.84 | 0.43 | 0.91 | 0.88 |
| snp27797-scaffold298-41343 | 13 | 62273520 | A | G | 0.55 | 0.41 | 0.68 | 0.91 | 0.95 | 0.58 |
| snp27799-scaffold298-154643 | 13 | 62386572 | G | A | 0.67 | 0.26 | 0.92 | 0.83 | 0.93 | 0.57 |
| snp6538-scaffold1231-784814 | 13 | 62434555 | A | G | 0.78 | 0.82 | 0.87 | 0.85 | 0.41 | 0.61 |
| snp6537-scaffold1231-750376 | 13 | 62468886 | G | A | 0.95 | 0.44 | 1.00 | 0.76 | 0.62 | 0.78 |
| snp6536-scaffold1231-692487 | 13 | 62527100 | A | G | 0.95 | 0.76 | 0.89 | 0.98 | 0.88 | 0.68 |
| snp6535-scaffold1231-657117 | 13 | 62562766 | A | G | 0.67 | 0.68 | 0.82 | 0.19 | 0.43 | 0.49 |
| snp6533-scaffold1231-581545 | 13 | 62638197 | G | A | 0.33 | 0.29 | 0.24 | 0.81 | 0.61 | 0.53 |
| snp6532-scaffold1231-540159 | 13 | 62679944 | G | A | 0.78 | 0.79 | 0.82 | 0.33 | 0.95 | 0.66 |
| snp6531-scaffold1231-502221 | 13 | 62717706 | G | A | 0.40 | 0.82 | 0.79 | 0.91 | 0.78 | 0.68 |

| | | | | | | | | | | |
|---|---|---|---|---|---|---|---|---|---|---|
| snp6530-scaffold1231-438108 | 13 | 62783588 | G | A | 0.53 | 0.41 | 0.42 | 0.81 | 0.49 | 0.40 |
| snp6529-scaffold1231-400642 | 13 | 62821046 | G | A | 0.67 | 0.50 | 0.55 | 0.78 | 0.57 | 0.70 |
| snp6528-scaffold1231-360622 | 13 | 62860953 | G | A | 0.43 | 0.62 | 0.58 | 0.87 | 0.55 | 0.39 |
| snp6527-scaffold1231-320179 | 13 | 62900048 | A | G | 0.74 | 0.79 | 0.50 | 0.87 | 0.97 | 0.89 |
| snp6526-scaffold1231-267192 | 13 | 62951083 | A | G | 0.17 | 0.59 | 0.58 | 0.07 | 0.96 | 0.44 |
| snp6525-scaffold1231-224623 | 13 | 62993649 | A | G | 0.55 | 0.76 | 0.66 | 0.30 | 0.76 | 0.74 |
| snp6524-scaffold1231-179272 | 13 | 63039600 | G | A | 0.86 | 0.79 | 0.95 | 0.98 | 1.00 | 0.37 |
| snp6522-scaffold1231-103243 | 13 | 63117523 | A | G | 0.34 | 0.65 | 0.68 | 0.31 | 0.76 | 0.66 |
| snp55185-scaffold849-19136 | 13 | 63480879 | A | C | 0.60 | 0.59 | 0.74 | 0.78 | 0.49 | 0.66 |
| snp12122-scaffold1449-23019 | 13 | 63523102 | A | G | 0.28 | 0.41 | 0.47 | 0.78 | 0.47 | 0.31 |
| snp12123-scaffold1449-73636 | 13 | 63573027 | G | A | 0.79 | 0.71 | 0.66 | 0.26 | 0.76 | 0.90 |
| snp12124-scaffold1449-129140 | 13 | 63628575 | G | A | 0.71 | 0.82 | 0.61 | 0.24 | 0.75 | 0.59 |
| snp12125-scaffold1449-187249 | 13 | 63686657 | G | A | 0.84 | 0.82 | 0.68 | 0.28 | 0.95 | 0.73 |
| snp12126-scaffold1449-231513 | 13 | 63730956 | A | G | 0.47 | 0.76 | 0.74 | 0.85 | 0.34 | 0.64 |
| snp12127-scaffold1449-287213 | 13 | 63786338 | G | A | 0.74 | 0.71 | 0.61 | 0.33 | 0.70 | 0.64 |
| snp12128-scaffold1449-337427 | 13 | 63836533 | G | A | 0.83 | 0.79 | 0.84 | 0.89 | 0.70 | 0.61 |
| snp12129-scaffold1449-367636 | 13 | 63866605 | A | G | 0.81 | 0.91 | 0.84 | 0.28 | 0.59 | 0.52 |
| snp12130-scaffold1449-404096 | 13 | 63903011 | G | A | 0.84 | 0.71 | 0.97 | 0.35 | 0.71 | 0.89 |
| snp12131-scaffold1449-486854 | 13 | 63986453 | G | A | 0.79 | 0.74 | 0.97 | 0.35 | 0.72 | 0.92 |
| snp12132-scaffold1449-518405 | 13 | 64018361 | A | G | 0.52 | 0.56 | 0.53 | 0.81 | 0.26 | 0.54 |
| snp5921-scaffold1209-20025 | 13 | 64064460 | A | G | 0.55 | 0.76 | 0.79 | 0.17 | 0.72 | 0.56 |
| snp5925-scaffold1209-222729 | 13 | 64266896 | A | G | 0.86 | 0.65 | 0.68 | 0.13 | 0.78 | 0.76 |
| snp5926-scaffold1209-252270 | 13 | 64296608 | A | G | 0.66 | 0.62 | 0.61 | 0.30 | 0.47 | 0.72 |
| snp5927-scaffold1209-294269 | 13 | 64338053 | G | A | 0.62 | 0.65 | 0.32 | 0.30 | 0.80 | 0.76 |
| snp5928-scaffold1209-336872 | 13 | 64380407 | A | G | 0.81 | 0.79 | 0.84 | 0.87 | 0.61 | 0.84 |
| snp5929-scaffold1209-372636 | 13 | 64416139 | A | G | 0.88 | 0.76 | 0.97 | 0.28 | 0.84 | 0.93 |
| snp5930-scaffold1209-402211 | 13 | 64445688 | A | G | 0.72 | 0.62 | 0.74 | 0.30 | 0.68 | 0.52 |
| snp5931-scaffold1209-464725 | 13 | 64508186 | A | G | 0.29 | 0.53 | 0.79 | 0.79 | 0.55 | 0.79 |
| snp5932-scaffold1209-512212 | 13 | 64555548 | A | G | 0.52 | 0.29 | 0.18 | 0.72 | 0.83 | 0.64 |
| snp5933-scaffold1209-543990 | 13 | 64587641 | A | G | 0.55 | 0.71 | 0.66 | 0.30 | 0.64 | 0.64 |
| snp5934-scaffold1209-599648 | 13 | 64643472 | A | G | 0.55 | 0.79 | 0.89 | 0.30 | 0.70 | 0.64 |
| snp5935-scaffold1209-650225 | 13 | 64694045 | A | G | 0.33 | 0.32 | 0.58 | 0.20 | 0.70 | 0.44 |
| snp5936-scaffold1209-699872 | 13 | 64743830 | G | A | 0.67 | 0.62 | 0.61 | 0.78 | 0.29 | 0.47 |
| snp5938-scaffold1209-790101 | 13 | 64834404 | A | G | 0.90 | 0.76 | 0.95 | 0.26 | 0.92 | 0.74 |
| snp5939-scaffold1209-820717 | 13 | 64864407 | G | A | 0.79 | 0.85 | 1.00 | 0.30 | 0.97 | 0.68 |
| snp5940-scaffold1209-863272 | 13 | 64906931 | G | A | 0.66 | 0.38 | 0.34 | 0.76 | 0.50 | 0.33 |
| snp5941-scaffold1209-913269 | 13 | 64956030 | A | G | 0.57 | 0.62 | 0.53 | 0.22 | 0.66 | 0.78 |
| snp5429-scaffold1188-2879889 | 13 | 64999299 | G | A | 0.76 | 0.91 | 0.55 | 0.91 | 0.32 | 0.76 |
| snp5428-scaffold1188-2846852 | 13 | 65032336 | G | A | 0.69 | 0.88 | 0.87 | 0.85 | 0.32 | 0.57 |
| snp5427-scaffold1188-2815388 | 13 | 65063907 | G | A | 0.88 | 0.79 | 0.66 | 0.96 | 0.49 | 0.76 |
| snp5426-scaffold1188-2785940 | 13 | 65093903 | G | A | 0.60 | 0.62 | 0.50 | 0.94 | 0.58 | 0.79 |
| snp5423-scaffold1188-2614332 | 13 | 65263105 | G | A | 0.29 | 0.85 | 0.74 | 0.22 | 0.75 | 0.78 |
| snp5421-scaffold1188-2522219 | 13 | 65355347 | A | G | 0.91 | 0.94 | 0.79 | 0.96 | 0.80 | 0.94 |

| | | | | | | | | | | |
|---|---|---|---|---|---|---|---|---|---|---|
| snp5417-scaffold1188-2340332 | 13 | 65537674 | G | A | 0.74 | 0.91 | 0.89 | 0.89 | 0.95 | 0.76 |
| snp5416-scaffold1188-2295194 | 13 | 65583170 | A | C | 0.50 | 0.56 | 0.66 | 0.61 | 0.39 | 0.76 |
| snp5415-scaffold1188-2221283 | 13 | 65654686 | G | A | 0.74 | 0.38 | 0.61 | 0.54 | 0.72 | 0.52 |
| snp5414-scaffold1188-2181656 | 13 | 65694441 | C | A | 0.33 | 0.50 | 0.71 | 0.63 | 0.43 | 0.66 |
| snp5412-scaffold1188-2047654 | 13 | 65827525 | A | G | 1.00 | 0.97 | 1.00 | 1.00 | 0.76 | 0.99 |
| snp5411-scaffold1188-1980699 | 13 | 65893885 | A | G | 0.71 | 0.68 | 0.89 | 0.67 | 0.46 | 0.73 |
| snp5410-scaffold1188-1944493 | 13 | 65929808 | G | A | 0.76 | 0.38 | 0.37 | 0.69 | 0.76 | 0.80 |
| snp5409-scaffold1188-1914715 | 13 | 65959346 | G | A | 0.59 | 0.47 | 0.50 | 0.48 | 0.42 | 0.51 |
| snp5408-scaffold1188-1885712 | 13 | 65988705 | G | A | 0.69 | 0.62 | 0.87 | 0.80 | 0.45 | 0.72 |
| snp5407-scaffold1188-1852940 | 13 | 66021511 | G | A | 0.83 | 0.82 | 0.56 | 0.22 | 0.83 | 0.69 |
| snp5405-scaffold1188-1779006 | 13 | 66095996 | G | A | 0.81 | 0.76 | 0.97 | 0.85 | 0.87 | 0.78 |
| snp5404-scaffold1188-1738098 | 13 | 66136130 | A | G | 0.62 | 0.82 | 0.79 | 0.78 | 0.47 | 0.57 |
| snp5403-scaffold1188-1707748 | 13 | 66166425 | A | G | 0.86 | 1.00 | 0.95 | 1.00 | 0.58 | 0.81 |
| snp5401-scaffold1188-1606247 | 13 | 66268297 | A | G | 0.74 | 0.74 | 0.82 | 0.85 | 0.87 | 0.73 |
| snp5400-scaffold1188-1575487 | 13 | 66299017 | G | A | 0.48 | 0.74 | 0.82 | 0.70 | 0.68 | 0.57 |
| snp5399-scaffold1188-1520444 | 13 | 66353850 | A | G | 0.69 | 0.38 | 0.74 | 0.83 | 0.64 | 0.71 |
| snp5395-scaffold1188-1372386 | 13 | 66501097 | G | A | 0.45 | 0.41 | 0.71 | 0.59 | 0.74 | 0.53 |
| snp5394-scaffold1188-1330690 | 13 | 66542955 | G | A | 0.86 | 0.88 | 0.39 | 0.74 | 0.42 | 0.54 |
| snp51098-scaffold742-443300 | 20 | 46495170 | A | G | 0.69 | 0.74 | 0.42 | 0.78 | 0.62 | 0.69 |
| snp51097-scaffold742-396098 | 20 | 46542884 | A | C | 0.47 | 0.68 | 0.79 | 0.83 | 0.62 | 0.68 |
| snp51093-scaffold742-203727 | 20 | 46736021 | A | G | 0.40 | 0.32 | 0.32 | 0.65 | 0.61 | 0.57 |
| snp51091-scaffold742-109218 | 20 | 46828372 | A | G | 0.69 | 0.68 | 0.84 | 0.70 | 0.43 | 0.71 |
| snp51090-scaffold742-61884 | 20 | 46875702 | G | A | 0.95 | 0.85 | 0.97 | 0.96 | 0.54 | 0.94 |
| snp51089-scaffold742-12793 | 20 | 46925747 | A | G | 0.83 | 0.91 | 0.84 | 0.94 | 0.38 | 0.54 |
| snp14428-scaffold1576-16025 | 20 | 46967509 | G | A | 0.64 | 0.88 | 0.79 | 0.94 | 0.24 | 0.52 |
| snp14429-scaffold1576-65789 | 20 | 47015860 | A | G | 0.72 | 0.82 | 0.32 | 0.78 | 0.68 | 0.50 |
| snp14430-scaffold1576-113393 | 20 | 47063742 | G | A | 0.36 | 0.91 | 0.39 | 0.83 | 0.51 | 0.52 |
| snp14431-scaffold1576-143213 | 20 | 47095159 | C | A | 0.78 | 0.47 | 0.89 | 0.43 | 0.66 | 0.70 |
| snp14432-scaffold1576-179983 | 20 | 47132893 | A | C | 0.48 | 0.79 | 0.66 | 0.33 | 0.43 | 0.62 |
| snp14433-scaffold1576-214232 | 20 | 47167387 | A | G | 0.41 | 0.71 | 0.37 | 0.57 | 0.72 | 0.70 |
| snp14434-scaffold1576-243719 | 20 | 47196921 | A | G | 0.55 | 0.97 | 0.97 | 0.76 | 0.58 | 0.96 |
| snp57432-scaffold916-65236 | 20 | 47311491 | A | G | 0.64 | 0.38 | 0.68 | 0.30 | 0.63 | 0.48 |
| snp57434-scaffold916-143906 | 20 | 47393808 | G | A | 0.81 | 0.71 | 0.55 | 0.37 | 0.61 | 0.27 |
| snp57435-scaffold916-184613 | 20 | 47434736 | G | A | 0.71 | 0.38 | 0.21 | 0.70 | 0.64 | 0.44 |
| snp57437-scaffold916-265022 | 20 | 47516402 | G | A | 0.71 | 0.91 | 0.45 | 0.57 | 0.58 | 0.62 |
| snp57438-scaffold916-295036 | 20 | 47546381 | G | A | 0.50 | 0.68 | 0.76 | 0.89 | 0.83 | 0.86 |
| snp57439-scaffold916-324919 | 20 | 47576607 | A | G | 0.72 | 0.79 | 0.45 | 0.35 | 0.34 | 0.56 |
| snp57440-scaffold916-366019 | 20 | 47618821 | A | G | 0.79 | 0.76 | 0.45 | 0.70 | 0.46 | 0.62 |
| snp57441-scaffold916-432419 | 20 | 47685798 | G | A | 0.69 | 0.79 | 0.50 | 0.44 | 0.67 | 0.90 |
| snp57443-scaffold916-509375 | 20 | 47763309 | A | C | 0.33 | 0.68 | 0.55 | 0.57 | 0.50 | 0.50 |
| snp57444-scaffold916-565285 | 20 | 47819874 | A | G | 0.76 | 0.65 | 0.61 | 0.57 | 0.46 | 0.71 |
| snp57445-scaffold916-634822 | 20 | 47890918 | A | G | 0.64 | 0.53 | 0.47 | 0.81 | 0.47 | 0.47 |
| snp34080-scaffold40-5934922 | 20 | 47942190 | A | C | 0.59 | 0.85 | 0.55 | 0.83 | 0.83 | 0.67 |

| | | | | | | | | | | |
|---|---|---|---|---|---|---|---|---|---|---|
| snp34077-scaffold40-5827789 | 20 | 48049951 | G | A | 0.64 | 0.21 | 0.68 | 0.76 | 0.33 | 0.59 |
| snp34075-scaffold40-5751561 | 20 | 48127119 | C | A | 0.60 | 0.06 | 0.47 | 0.61 | 0.25 | 0.52 |
| snp34074-scaffold40-5696142 | 20 | 48183408 | A | G | 0.60 | 0.09 | 0.76 | 0.78 | 0.41 | 0.58 |
| snp34073-scaffold40-5648593 | 20 | 48231250 | C | A | 0.36 | 0.32 | 0.61 | 0.39 | 0.75 | 0.64 |
| snp34072-scaffold40-5599020 | 20 | 48281756 | G | A | 0.52 | 0.26 | 0.39 | 0.65 | 0.42 | 0.56 |
| snp34070-scaffold40-5503844 | 20 | 48377212 | A | G | 0.60 | 0.59 | 0.61 | 0.24 | 0.43 | 0.60 |
| snp34069-scaffold40-5468396 | 20 | 48412677 | C | A | 0.31 | 0.56 | 0.18 | 0.83 | 0.76 | 0.54 |
| snp34068-scaffold40-5436207 | 20 | 48445048 | G | A | 0.84 | 0.85 | 0.39 | 0.17 | 0.38 | 0.64 |
| snp34067-scaffold40-5406654 | 20 | 48474629 | G | A | 0.50 | 0.82 | 0.61 | 0.67 | 0.63 | 0.56 |
| snp34065-scaffold40-5340021 | 20 | 48542170 | G | A | 0.76 | 0.76 | 0.84 | 0.48 | 0.80 | 0.63 |
| snp34063-scaffold40-5264553 | 20 | 48620688 | G | A | 0.71 | 0.74 | 0.92 | 0.72 | 0.64 | 0.79 |
| snp34062-scaffold40-5230608 | 20 | 48654667 | C | A | 0.81 | 0.97 | 0.84 | 0.98 | 0.82 | 0.68 |
| snp34061-scaffold40-5191067 | 20 | 48694292 | G | A | 0.66 | 0.76 | 0.61 | 0.96 | 0.84 | 0.73 |
| snp34059-scaffold40-5127504 | 20 | 48758806 | G | A | 0.43 | 0.38 | 0.37 | 0.74 | 0.64 | 0.47 |
| snp34058-scaffold40-5092328 | 20 | 48794486 | A | G | 0.67 | 0.68 | 0.42 | 0.28 | 0.42 | 0.58 |
| snp34056-scaffold40-5015633 | 20 | 48871142 | G | A | 0.41 | 0.76 | 0.74 | 0.78 | 0.58 | 0.60 |
| snp34054-scaffold40-4944480 | 20 | 48941295 | A | G | 0.52 | 0.24 | 0.68 | 0.70 | 0.41 | 0.71 |
| snp34051-scaffold40-4763094 | 20 | 49123937 | A | G | 0.76 | 0.38 | 0.37 | 0.39 | 0.72 | 0.76 |
| snp34050-scaffold40-4706252 | 20 | 49180942 | A | G | 0.78 | 0.74 | 0.66 | 0.80 | 0.76 | 0.63 |
| snp34046-scaffold40-4552204 | 20 | 49335536 | A | G | 0.52 | 0.47 | 0.71 | 0.31 | 0.53 | 0.79 |
| snp34045-scaffold40-4521116 | 20 | 49367313 | A | C | 0.47 | 0.21 | 0.74 | 0.39 | 0.47 | 0.73 |
| snp34044-scaffold40-4483699 | 20 | 49404860 | A | G | 0.88 | 0.97 | 0.32 | 0.89 | 0.96 | 0.50 |
| snp34041-scaffold40-4319824 | 20 | 49568821 | C | A | 0.71 | 0.82 | 0.79 | 0.74 | 0.66 | 0.84 |
| snp34040-scaffold40-4261985 | 20 | 49629932 | A | G | 0.81 | 0.91 | 0.53 | 0.35 | 0.74 | 0.78 |
| snp34039-scaffold40-4225256 | 20 | 49667564 | G | A | 1.00 | 0.59 | 0.95 | 0.83 | 0.70 | 0.63 |
| snp34037-scaffold40-4148997 | 20 | 49743955 | G | A | 0.53 | 0.71 | 0.82 | 0.83 | 0.74 | 0.69 |
| snp34036-scaffold40-4095297 | 20 | 49798016 | A | G | 0.71 | 0.82 | 0.47 | 0.31 | 0.49 | 0.30 |
| snp34035-scaffold40-4041489 | 20 | 49852306 | G | A | 0.90 | 0.94 | 0.95 | 0.72 | 0.65 | 0.60 |
| snp34033-scaffold40-3961856 | 20 | 49927473 | G | A | 0.72 | 0.94 | 0.92 | 0.93 | 0.47 | 0.60 |
| snp34032-scaffold40-3932831 | 20 | 49956533 | G | A | 0.69 | 0.44 | 0.45 | 0.72 | 0.39 | 0.57 |
| snp34031-scaffold40-3900976 | 20 | 49988401 | A | G | 0.91 | 0.82 | 0.82 | 0.80 | 0.84 | 0.72 |
| snp34029-scaffold40-3825423 | 20 | 50063724 | G | A | 0.86 | 0.97 | 0.82 | 0.89 | 0.50 | 0.83 |
